# A Recursive Stochastic Algorithm for Real-Time Online Parameter Estimation in Item Response Theory: Enhancing Computational Efficiency for Dynamic Educational Assessment

**DOI:** 10.1017/psy.2025.10064

**Published:** 2025-12-23

**Authors:** Sainan Xu, Jing Lu, Jiwei Zhang

**Affiliations:** 1 School of Mathematics and Statistics, Key Laboratory of Applied Statistics of MOE, Key Laboratory of Big Data Analysis of Jilin Province, Northeast Normal University, Changchun, Jilin, China; 2 Faculty of Education, Key Laboratory of Applied Statistics of MOE, Northeast Normal University, Changchun, Jilin, China

**Keywords:** item response theory, large-scale testing, online parameter estimation, real-time response data, stochastic Newton algorithm

## Abstract

Traditional large-scale educational data are typically static and updated periodically, making it difficult to capture the dynamic changes in real time. However, recent technological advancements allow online exam platforms to collect students’ response data in real time. While item response theory (IRT) estimation methods are widely recognized for their accuracy, they are primarily designed for offline environments. When real-time data continuously arrives and online parameter estimation is required, these methods become computationally impractical. To address this challenge, we propose a recursive stochastic algorithm, i.e., truncated average stochastic Newton algorithm (TASNA), for the efficient online parameter estimation within the IRT framework. This algorithm significantly improves computational efficiency compared to the expectation–maximization (EM) algorithm implemented in the mirt package in R. The algorithm offers a powerful alternative to the traditional offline EM method. Furthermore, we investigate the asymptotic properties of the algorithm, proving its almost sure convergence and asymptotic normality. Numerical experiments using both simulated and real data demonstrate the practicality of the proposed method.

## 1 Introduction

In recent years, large-scale educational assessments, such as the Programme for International Student Assessment (PISA; OECD, 2021), the National Assessment of Educational Progress (NAEP; Johnson & Jenkins, 2004), the Trends in International Mathematics and Science Study (TIMSS; Martin & Kelly, 1996), and the Progress in International Reading Literacy Study (PIRLS; Mullis et al., 2003), have become central to global education research. These assessments produce large-scale and multifaceted datasets, providing valuable insights into student knowledge, skills, and learning behaviors. However, traditional large-scale educational data is typically static and updated periodically, making it challenging to capture the dynamic changes in educational environments and the real-time status of students’ learning. With advances in information technology, online testing platforms and educational assessment tools like adaptive testing now allow the collection of real-time data on students’ responses and behaviors, providing new possibilities for dynamically evaluating educational processes and learning outcomes. Real-time data are characterized by its high speed, large volume, and complexity, requiring efficient computational methods to quickly identify patterns, detect learning obstacles, and predict outcomes. In addition, leveraging this real-time data allows educators to adjust teaching strategies promptly and offer personalized learning experiences for students.

The increasing availability and complexity of large-scale educational datasets have highlighted the need for robust analytical frameworks to handle such data efficiently. Item response theory (IRT; van der Linden & Hambleton, 1997) has emerged as a powerful tool for analyzing such large datasets. In IRT, parameter estimation is a core component that directly determines the model’s scientific validity and the interpretation of results. The most commonly used estimation method in IRT is marginal maximum likelihood estimation based on the expectation–maximization algorithm (MMLE-EM; Baker & Kim, 2004; Bock & Aitkin, 1981). MMLE-EM utilizes an iterative optimization approach that is highly efficient in managing missing data or complex item response datasets, making it particularly adaptable to large-scale educational assessments. However, the computational complexity of this method is primarily influenced by the dimensionality of the latent variables. As the dimensionality of the latent variables increases linearly, the number of quadrature nodes required for integration in the E-step grows exponentially, leading to a substantial increase in computational cost and difficulty.

To address these challenges, current research on IRT focuses on improving computational efficiency for high-dimensional numerical integration. Several methods have been proposed, including adaptive Gaussian quadrature EM algorithms (Rabe-Hesketh et al., 2005; Schilling & Bock, 2005), Laplace approximation methods (Huber et al., 2004; Thomas, 1993), Monte Carlo EM algorithms (Meng & Schilling, 1996; Song & Lee, 2005), Markov chain Monte Carlo (MCMC) methods (Albert, 1992; Béguin & Glas, 2001; Jiang & Templin, 2019), stochastic approximation methods (Cai, 2010a,b; Zhang et al., 2020), and variational inference-based approaches (Cho et al., 2021; Urban & Bauer, 2021; Wu et al., 2020). These methods improve the numerical integration steps of traditional MMLE-EM or introduce approximate inference methods, significantly enhancing parameter estimation accuracy and computational efficiency in high-dimensional scenarios. However, the implementation of these techniques typically relies on complete and static dataset. In real-time dynamic assessments, parameters need to be continuously updated as data streams in, and estimation methods that depend on a complete dataset incur high computational costs. As a result, there is a growing need for faster and more efficient online parameter estimation methods that can update in real time without relying on a complete dataset.

Recently, with the rapid growth of computer-based testing, online algorithms have received widespread attention in statistics and machine learning (e.g., Duchi et al., 2011; Godichon-Baggioni, 2019; Godichon-Baggioni & Lu, 2024; Saad, 2009). These algorithms update parameters in real time by processing data incrementally, reducing memory requirements and making them well-suited for dynamic data streams and large-scale datasets. The stochastic Newton algorithm (SNA) has become a prominent tool in online optimization, offering strong performance in accelerating convergence, addressing high-dimensional challenges, and updating parameters efficiently in real time. For example, Bercu et al. (2020) proposed a truncated SNA for logistic regression and established its optimal asymptotic behavior. Boyer and Godichon-Baggioni (2023) introduced a weighted averaged SNA within a general framework, proving its almost sure convergence rate and asymptotic normality. Cénac et al. (2025) developed an online stochastic Gaussian–Newton algorithm and its averaged variant for nonlinear models, demonstrating its asymptotic efficiency. These developments underline the practical and theoretical strengths of SNA in large-scale, real-time optimization tasks.

Online parameter estimation for IRT models has become a significant challenge due to the dynamic nature of assessments. Traditional offline methods often require access to the entire dataset, which is impractical for real-time applications. To address this, Weng and Coad (2018) proposed a real-time parameter estimation method based on deterministic moment-matching, enabling real-time updates of IRT model parameters within a Bayesian framework. Subsequently, Jiang et al. (2023) further extended this method to adaptive learning systems, partially addressing parameter estimation challenges in dynamic learning scenarios. However, this approach is limited to estimating only the mean and variance of the parameters, without directly estimating the specific parameters, which restricts its practical applicability.

To overcome this limitation, this study proposes a recursive SNA—referred to as the truncated averaged SNA (TASNA), in the IRT framework. This method incorporates an incremental update mechanism, enabling real-time estimation of both item and examinee ability parameters as response data arrives, without requiring access to or storage of previous response data. This design achieves dynamic and efficient parameter estimation, tailored for real-time applications in large-scale educational assessment.

The TASNA offers notable advantages in handling the real-time data streams and large-scale data in educational measurement. First, TASNA substantially reduces computational costs and memory requirements. TASNA utilizes an incremental update mechanism, processing only one examinee’s data at a time without revisiting previous data. This method lowers computational load and resource usage while significantly decreasing the real-time feedback time, making it well-suited for large-scale online assessments and real-time feedback systems. Second, this study provides a rigorous theoretical analysis of the asymptotic properties of TASNA, demonstrating the almost sure convergence and asymptotic normality of the estimated parameters. This provides a theoretical foundation that ensures the algorithm’s reliability and stability. Moreover, TASNA achieves superior execution efficiency compared to the EM algorithm available in the R package mirt, particularly in large-scale data scenarios, as verified by simulation studies. Therefore, TASNA is not only suitable for real-time parameter estimation in online data streams, but also serves as an efficient alternative to traditional EM algorithms in offline large-scale educational data environments. In brief, the proposed TASNA combines incremental updating mechanisms, efficient stochastic optimization strategies, and theoretical reliability to establish itself as an innovative solution for real-time IRT parameter estimation in dynamic educational assessment.

This article is organized as follows. Section 2 introduces the two-parameter logistic (2PL) model and the multidimensional 2PL (M2PL) model. In Section 3, the proposed online estimation method, the TASNA, is presented. Section 4 presents the theoretical assumptions and the asymptotic properties of the method. Section 5 provides simulation results to evaluate the performance of the TASNA in both the 2PL and M2PL models. In Section 6, an example is provided to demonstrate the application of the TASNA in IRT models. Finally, Section 7 offers concluding remarks.

## 2 Model presentation

This article focuses on two widely used item response models: the unidimensional 2PL model and the M2PL model. Consider a test with *N* examinees and *J* items. Let 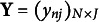 represent the observed response data matrix, where each entry 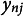 is either 0 or 1. If 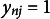 , the *n*th examinee correctly answered the *j*th item, whereas 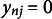 indicates an incorrect response, with 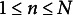 and 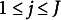 .

In the 2PL model, let 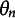 be the ability parameter of the *n*th examinee, and 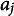 and 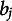 the discrimination and difficulty parameters of the *j*th item, respectively. The correct response probability of the 2PL model is given by 
(1)

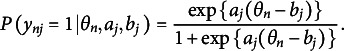



The M2PL model extends the 2PL model by incorporating a multidimensional framework. Here, the unidimensional latent ability 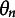 of examinee *n* is replaced by a *Q*-dimensional vector 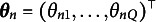 , which represents the ability levels of examinee *n* across multiple latent dimensions. The discrimination parameters for item *j* become *Q*-dimensional vector 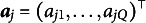 , indicating how item *j* differentiates examinees based on their latent abilities along each dimension of the multidimensional construct 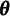 . If 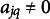 , it indicates that item *j* is associated with the *q*th latent trait, meaning that the item contributes to measuring the ability on the *q*th dimension. The intercept parameter of item *j*, 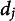 , is a scalar and does not vary across dimensions. The correct response probability of the M2PL model is provided as follows: 
(2)

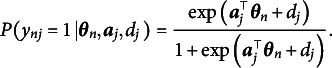

For simplicity in presenting the algorithm, we redefine the parameter notations using the M2PL model as an example. Let the item parameters be 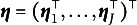 , where 
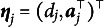
 in the M2PL model (and 
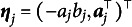
 for the 2PL model). Furthermore, we define 
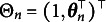
 . Thus, the M2PL model can be rewritten as follows: 
(3)

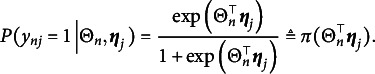

The response data 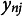 follow a Bernoulli distribution with a success probability 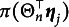 , and it can be expressed as follows: 

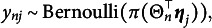

where 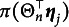 represents the probability of a correct response, determined by the linear predictor 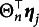 passed through a logistic function. Our objective is to perform online estimation of the parameters for the items, 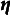 , and the latent ability parameter for each individual, 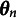 , as the response data for the *n*th individual arrives. The term “online estimation” refers to dynamically updating the estimates of the parameters in real time, rather than batch-processing all data at once, enabling the model to adapt continuously as new response data is received.

## 3 Stochastic Newton online estimation algorithm

Before introducing the stochastic Newton algorithm (SNA), it is necessary to briefly review the EM algorithm in the IRT framework. The EM algorithm primarily consists of two steps: the E-step and the M-step. In the E-step, each iteration requires the computation of expectations for all examinees and items. While the calculation for a single iteration is relatively straightforward, it becomes highly time-consuming when the number of examinees and items is large. Moreover, due to the characteristics of IRT models, the M-step of the EM algorithm often involves using Newton’s iterative method to maximize the parameters. Newton’s method is a deterministic optimization algorithm that operates on the entire dataset. Specifically, each Newton iteration requires calculating the gradient and Hessian matrix of the objective function based on the entire dataset to determine the update direction for the parameters. This results in high computational complexity, particularly when running on large-scale datasets, making the process extremely time-intensive. Therefore, when dealing with large datasets or when response data arrive continuously and real-time parameter estimation is required, the EM algorithm may struggle to perform fast and efficient computations. This limitation highlights the necessity of exploring more efficient algorithms, such as the SNA, which offers significant advantages for large-scale real-time estimation tasks.

The SNA shares a structural similarity with the traditional Newton algorithm, as both rely on the gradient and Hessian matrix of the objective function to determine the parameter update direction. However, the key difference lies in how these quantities are computed. The traditional Newton algorithm requires computing the gradient and Hessian matrix based on the entire dataset, whereas the SNA (Bercu et al., 2020; Boyer & Godichon-Baggioni, 2023; Cénac et al., 2025; Godichon-Baggioni & Lu, 2024) estimates these quantities incrementally through a recursive process. This approach avoids exhaustive computations on the entire dataset, significantly reducing the consumption of computational resources, making it particularly suitable for large-scale data applications and efficient handling of online data streams.

### 3.1 Item parameter updates via the truncated average stochastic Newton algorithm

Taking the M2PL model as an example, we first introduce the recursive SNA for estimating item parameters. To align with commonly accepted conventions, we assume that the latent vector 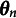 for examinee 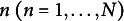 follows a multivariate normal distribution with a mean vector of 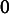 and an identity matrix as its variance–covariance matrix. When the response data 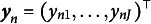 of the *n*th examinee are received, the marginal probability for examinee *n* is defined as follows: 
(4)

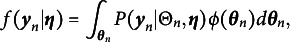

where 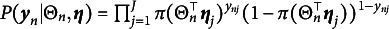 . The integral of 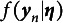 is typically evaluated using an integral approximation procedure. When the dimensionality is five or fewer, the fixed-point Gauss–Hermite quadrature method (Bock & Aitkin, 1981) is commonly employed. For higher-dimensional cases, adaptive quadrature techniques are used (Schilling & Bock, 2005). To simplify the explanation without losing generality, the expression of 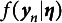 after approximation using the fixed-point Gauss–Hermite quadrature technique is given below: 
(5)

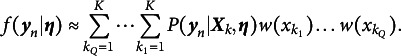

Here, 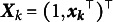 , 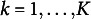 , where 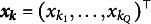 represents the vector of values at the specified quadrature nodes and *K* denotes the number of quadrature nodes in a single dimension. Each 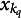 denotes the value of a quadrature node in dimension *q*, and 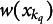 is the weight associated with the quadrature node in dimension *q*. The weight 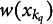 depends on the height of the normal density function at that node and the spacing between adjacent quadrature nodes. The index 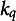 represents the position of the *K* quadrature nodes in dimension *q*. Traditional offline methods typically rely on batch data to compute the global objective function. In contrast, the SNA updates the parameters using only the current single sample data rather than the entire dataset. Consequently, the objective function (defined in this article as the negative log-likelihood function) for the *n*th examinee is 
(6)

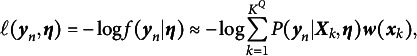

where 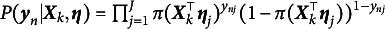 with 
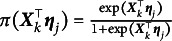
 . The summation 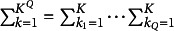 , and 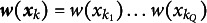 is the product of the weights for the respective quadrature nodes.

The SNA updates the parameters incrementally as data arrive. For each newly arrived response data, the item parameters are updated using the current response data and the parameter estimates from the previous iteration. When the response data of the *n*th examinee arrive, the empirical gradient vector and Hessian matrix (i.e., the first and second derivatives computed with respect to the current sample) for the *j*th item parameter 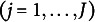 are derived based on the parameters 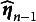 from the previous iteration, as follows (refer to Chapter 6 of Baker and Kim (2004) for detailed computations): 
(7)

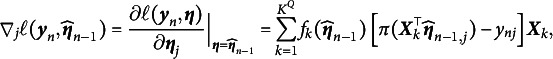



(8)

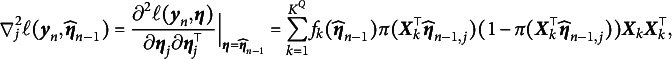

where 
(9)

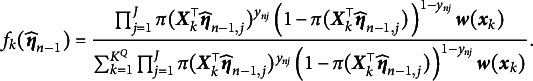

Note that Equation (8) represents the second-order derivative matrix for the newly arrived data and does not approximate the true Hessian matrix. However, for large-scale datasets or real-time data streams, directly computing the true Hessian matrix is computationally expensive. To address this, historical Hessian information is accumulated using a recursive formula, allowing the approximation of the Hessian matrix to be updated incrementally as new data arrive. This approach enables the algorithm to update model parameters efficiently while incorporating new data, without the need to re-process the entire dataset. Leveraging historical information accelerates convergence and enhances the algorithm’s robustness and computational efficiency. The recursive formula for updating the Hessian matrix is provided below for 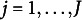 : 
(10)

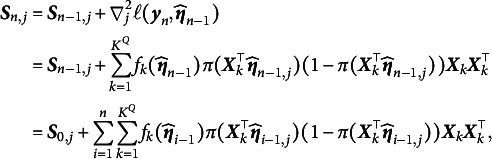

where 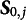 represents the initial value of the Hessian matrix, which is symmetric and positive definite, and 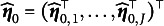 denotes the initial item parameter estimates. By recursively updating the Hessian matrix estimate, the SNA significantly reduces the computational cost of each iteration, making it more suitable for large-scale datasets and online learning context. The updated formula for the SNA within the IRT framework is provided below: 
(11)

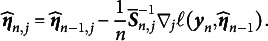

Here, 
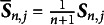
 . Boyer and Godichon-Baggioni (2023) and Cénac et al. (2025) argued that the original SNA decreases at a step rate of 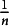 , which may hinder the dynamics of the algorithm and lead to suboptimal results in cases of improper initialization. To address this issue, we introduce a new step size sequence 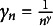 , where 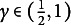 , and incorporate an averaging step to ensure the asymptotic efficiency of the algorithm. However, the convergence properties of the parameters for the original SNA cannot be established. Therefore, following the approach of Bercu et al. (2020), we present a truncated averaged SNA (TASNA). The update process for the *j*th item parameter ( 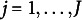 ) is given below: 
(12)

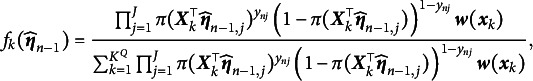



(13)

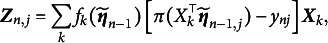



(14)

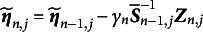



(15)

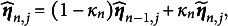



(16)

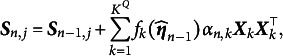

where 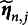 and 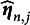 represent the *j*th item parameter estimates before and after applying the averaging step, respectively. The step sequence is defined as 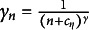 , where the step parameter 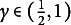 . The averaging step is defined as 

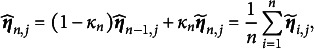

with the averaging sequence 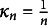 . The term 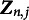 denotes the updated gradient 
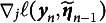
 , and 
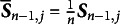
 represents the scaled Hessian matrix from the previous iteration. It is important to note that the update of 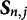 depends on the averaged parameter 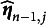 rather than 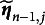 , enabling faster convergence. The value of 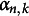 is given by 

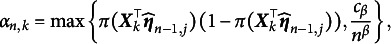

which is used for truncating the Hessian matrix.

Remark 1. The constant 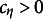 is added to the denominator of 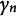 to mitigate the effect of poorer estimates produced by the algorithm during the initial iterations (when *n* is small). As *n* increases, this effect diminishes, and the quality of the iterative results improves.

Remark 2. When 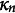 is fixed at 1, the TASNA reduces to the TSNA. TSNA is a special case of TASNA. By performing a weighted average over multiple stochastic gradient calculations, TASNA reduces the impact of random noise. Although TASNA requires slightly more computational time compared to TSNA (due to the averaging step), it achieves smoother convergence and more stable parameter estimation. In contrast, TSNA updates parameters in each iteration solely based on the current batch of samples, without a global averaging step. As a result, its convergence may be slower and exhibit greater fluctuations. In resource-constrained scenarios, TSNA can be used to trade off some convergence accuracy for improved computational efficiency. However, for more stable and accurate parameter estimation, TASNA is recommended.

Remark 3. For some positive constant 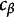 , the sequence 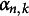 is defined as 
(17)

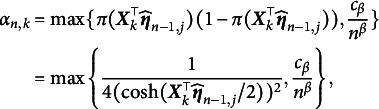

where 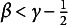 . Since the hyperbolic cosine function 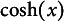 takes values in the range 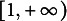 , we assume that 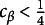 , which ensures 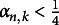 for 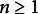 . Under this truncation operation, the convergence of the Hessian matrix estimation can be proven. Please see Section 4 for details.

### 3.2 Updating ability parameters by expected a posteriori estimation

For estimating the examinees’ ability parameters, researchers typically rely on methods, such as maximum likelihood estimation (MLE), maximum a posteriori estimation (MAP), or expected a posteriori estimation (EAP) after the item parameters have been estimated (Bock & Aitkin, 1981; Wang, 2015). It is well known that MLE relies on batch data to compute the global likelihood, which indicates the entire likelihood function must be recalculated whenever new data are added. This makes MLE computationally expensive in online environments where data continuously stream in, rendering it unsuitable for efficient online updating. In contrast, MAP incorporates prior information by maximizing the posterior probability. Although it is more flexible than MLE due to its ability to include prior information, it typically requires a large amount of historical data for global optimization. Furthermore, because of the structure of IRT models, MAP estimation often relies on iterative optimization methods, such as Newton’s method or gradient descent, to maximize the posterior probability. This iterative process further increases computational complexity and time. Detailed implementations of these two methods can be found in Wang (2015) and Bock and Aitkin (1981).

Unlike the two methods mentioned above, EAP estimation computes the posterior expectation of the ability parameter 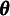 as a point estimate. The process for EAP estimation of 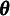 has been outlined by Bock and Aitkin (1981), Muraki and Engelhard Jr. (1985), and Wang (2015). EAP estimation has the advantage of ensuring that the estimated ability parameter derived from item scores, even with short test items, does not diverge toward infinity. This makes it particularly suitable for cases where data are limited or strong prior information is available (Reckase, 2009). The mathematical expression for this estimation is as follows: 
(18)

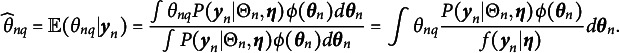

Here, 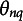 represents the *q*th dimension of the latent trait for the *n*th examinee, where 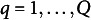 . Given known item parameters 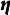 , the integral in Equation (18) can be approximated using the Gauss–Hermite quadrature method. The approximated expression for 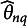 is 
(19)

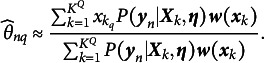

Therefore, 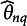 depends not only on the quadrature node vector and its weights but also on the data 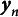 and the item parameters 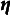 . In other words, when the response data for the *n*th examinee arrive, the examinee’s latent traits can be quickly updated using EAP estimation, provided that the item parameters have already been updated. Given the real-time estimates of the item parameters 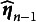 , the formula for updating the ability parameters is given by 
(20)

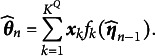



### 3.3 Implementation of stochastic Newton online estimation algorithms

The specific algorithmic flow of the online parameter estimation method in the IRT framework is outlined in Algorithm 1.

**Figure:**
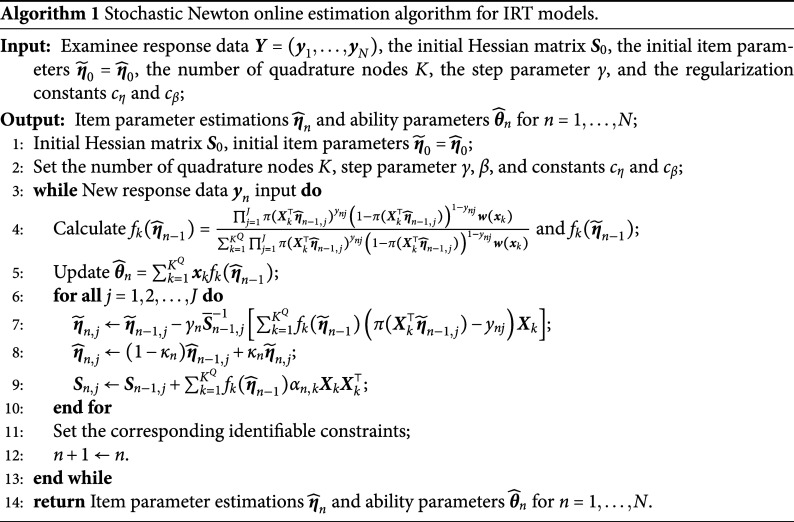

## 4 Theoretical properties

The convergence properties of the recursive SNA are crucial for ensuring the theoretical performance, practical stability, and efficiency of the algorithm. While some algorithms are guaranteed to converge in theory, they may require very small step sizes, leading to long convergence time before reaching the optimal solution. By deriving the convergence properties, we can better quantify these performance aspects, which aids in selecting appropriate parameters and optimization strategies. In this section, we derive the consistency, convergence rate, and asymptotic normality of parameter estimation using the TASNA.

Consider that the arriving response data vector 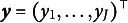 from an examinee consists of independent Bernoulli random variables, where each response 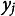 follows 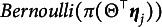 , with 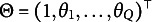 representing the examinee’s latent traits, and 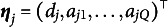 denoting the item parameters for 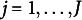 . The objective function, using the Gauss–Hermite quadrature method, is defined as follows: 
(21)

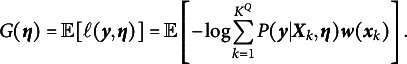

It is important to note that all theoretical results in this study are based on the numerically approximated objective function 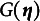 , meaning the algorithm optimizes 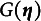 rather than the exact marginal log-likelihood. Under certain standard convexity assumptions on *G*, our goal can equivalently be stated as minimizing 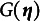 to obtain the item parameter estimates, that is, 
(22)

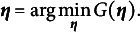

Thus, the gradient and Hessian matrix for the *j*th item parameter 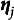 are given by 
(23)

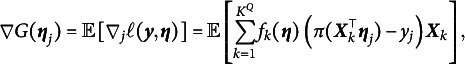



(24)

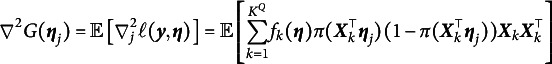



(25)

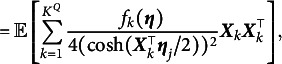

where 
(26)

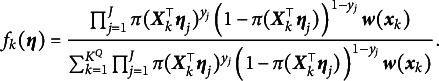



To ensure the effectiveness and convergence of the TASNA, the following regularity assumptions are imposed:

- The item parameters and the latent ability parameters of all examinees are bounded.
- The latent ability parameters 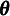 of each examinee are independent and follow the same multivariate normal distribution with a mean vector of 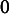 and identity covariance matrix 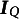 .
- There exists a positive constant 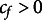 such that, for any item parameter vector 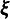 , 

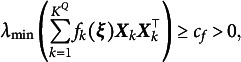

where 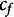 is independent of the sample size *n* and the index *j*.

Assumption (A1) guarantees the boundedness of the parameter space, which ensures that both the gradient and the Hessian matrix of the objective function remain bounded within this space. Combined with Assumption (A2), since the latent abilities satisfy 
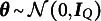
 , the Gauss–Hermite quadrature nodes are symmetrically distributed around the origin and possess finite fourth-order moments. These properties are essential for the stability of the numerical integration process. Assumption (A3) further ensures the positive definiteness of the Hessian matrix 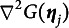 , which is critical for guaranteeing the uniqueness of the solution to the minimization problem. Together, these assumptions ensure that the objective function 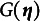 is twice continuously differentiable and that its minimizer is unique. This satisfies the two key regularity conditions proposed by Bercu et al. (2020). Under these assumptions, we first present the consistency of the item parameter estimates 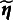 , 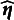 , and the consistency of the Hessian matrix estimates 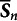 in the following theorem.

Theorem 1 **(Consistency).**

Under Assumptions (A1)–(A3), for all 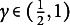 and 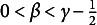 , the following result holds:

(27)

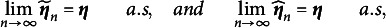

and for 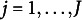 , 
(28)

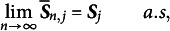

where 
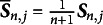
 , 
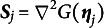
 , 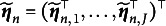 , 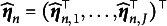 , and 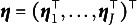 .

The proof of Theorem 1 is provided in Section C.1 of the Supplementary Material. Theorem 1 establishes that, as the sample size *n* tends to infinity, the item parameter estimates obtained using TASNA converge to the true parameter values 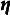 . Additionally, we demonstrate the consistency of the Hessian matrix estimate. We then present the convergence rates for both the item parameter estimates and the Hessian matrix estimates. This analysis of convergence rates offers insight into how rapidly the estimates approach their true values as the sample size *n* increases.

Theorem 2 **(Convergence rate).**

Under Assumptions (A1)–(A3), for all 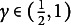 and 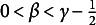 , we have the following results: 
(29)

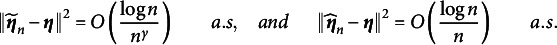

Furthermore, for 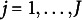 , 
(30)

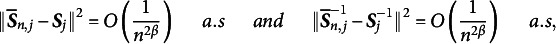

where 
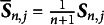
 , 
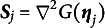
 , 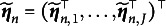 , 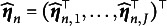 , and 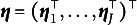 .

The proof of Theorem 2 is provided in Section C.2 of the Supplementary Material. Theorem 2 demonstrates that the convergence rate of the averaged parameter estimates is faster than that of the non-averaged parameter estimates. This implies that averaging the parameters during the iterative process enhances the convergence rate, enabling quicker stabilization of the parameter estimates as the sample size increases.

The convergence rate of the averaged parameter estimates is 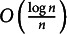 , which indicates that the error in the averaged parameter estimates decreases at a rate proportional to 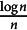 as the sample size *n* increases. This indicates that as the number of samples grows, the averaged estimates will steadily approach the true parameter values. The convergence rate of the Hessian matrix estimate is 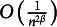 , implying that the error in the Hessian matrix estimate decreases rapidly as the sample size increases. The speed of this reduction depends on the value of 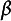 . The closer 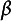 is to 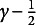 , the faster the convergence rate. This makes the algorithm highly efficient for large sample sizes, as it provides accurate Hessian estimates that improve with *n*. Therefore, in practical applications, 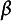 should be chosen to be close to 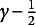 to maximize efficiency.

Finally, to establish a reliable statistical foundation for model estimation under the TASNA and to provide a theoretical basis for subsequent statistical inference, we present the asymptotic normality of the parameter estimates obtained using TASNA, as stated in the following theorem.

Theorem 3 **(Asymptotic normality).**

Under Assumptions (A1)–(A3), for 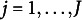 , the following asymptotic normality holds: 
(31)

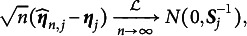

where 
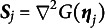
 .

The proof of Theorem 3 is provided in Section C.3 of the Supplementary Material. Theorem 3 implies that, as the sample size *n* increases, the scaled difference between the estimated parameters and the true parameters converges in distribution to a normal distribution with a zero mean and covariance matrix 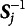 . This result establishes a foundation for conducting statistical inference, such as constructing confidence intervals or performing hypothesis testing, on the estimated parameters.

Remark 1. From Equation (20), the online EAP estimates of the ability parameters depend on the updated item parameters. Theorem 2 establishes the convergence properties of the updated item parameters. Consequently, based on Theorem 2 and Lemma 3 in the Supplementary Material, we can derive the convergence rate of the online EAP estimates of the ability parameters, denoted as 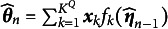 , relative to the EAP estimates of the ability parameters computed using the true item parameter values 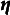 , denoted as 
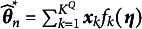
 , as follows: 
(32)

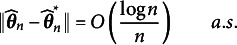

This result demonstrates that the error between 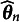 and 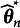 decreases as the sample size *n* increases, and the rate of this decrease is 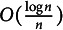 .

Remark 2. Based on Theorem 3 and Equation (28), the standard error of the estimated parameters can be naturally obtained by taking the inverse of the Hessian matrix estimated at the final sample size and extracting the square roots of its diagonal elements: 
(33)

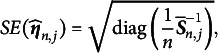

where 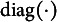 denotes taking the diagonal elements of a matrix. This approach provides both a theoretically sound and practically implementable method for computing standard errors within the TSNA and TASNA frameworks.

## 5 Simulation study

The simulation study aims to evaluate the effectiveness and practicality of the proposed online parameter estimation methods under the 2PL and M2PL models. We compare the performance of the TASNA and TSNA algorithms against the EM algorithm with fixed quadrature, evaluating algorithms in terms of bias, RMSE of parameter estimates, and computational time. The EM algorithm with fixed quadrature is implemented primarily using the mirt package in R (Chalmers, 2012). The mirt package leverages optimization techniques and Rcpp to enhance computational efficiency. Rcpp enables the integration of C++ code into R, allowing computationally intensive tasks to be executed efficiently. To ensure a fair comparison of computational efficiency, the TASNA and TSNA were rewritten in C++ using Rcpp and subsequently called from R to compare with the EM algorithm implemented in the mirt package.

### 5.1 Simulation designs

Five factors were manipulated to vary different simulation conditions: (1) The number of examinees, i.e., 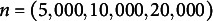 . (2) The number of items, i.e., 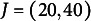 . (3) The dimensions of latent traits, i.e., 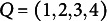 . 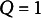 represents the unidimensional 2PL model. (4) The number of Gauss–Hermite quadrature nodes, i.e., 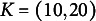 . The number of Gauss–Hermite quadrature nodes significantly affects the accuracy of numerical integration and the convergence of the algorithm. By simulating and comparing model performance at different values of *K*, we aim to identify an appropriate number of nodes that balances accuracy and computational efficiency. (5) Different step sizes, i.e., 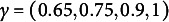 . An appropriate step size can accelerate the algorithm’s convergence while ensuring its stability and enhancing the accuracy of the final solution. By comparing different step size settings, the optimal step size strategy can be determined. Each simulation condition was replicated 50 times.

### 5.2 Data generation and model identifiability conditions

We generated item response data for the 2PL model and M2PL model based on Equation (1) and Equation (2), respectively. To simplify the computation and enhance the interpretability of the model, we assumed that the latent ability dimensions are independent. This assumption also eliminates the indeterminacy of scale, which is a common practice in other research (e.g., Cui et al., 2024). Therefore, the ability parameters 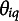 for each examinee *i* were sampled from a standard normal distribution 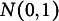 , where 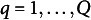 . Following Shang et al. (2023), for each item *j* and dimension *q*, the discrimination parameters 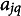 were generated from the uniform distribution 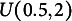 , while the difficulty parameters 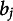 (for the 2PL model) and the intercept parameters 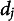 (for the M2PL model) were generated from the standard normal distribution 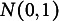 , for 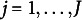 and 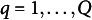 .

To ensure the identifiability of the 2PL model, we imposed constraints on the ability parameters by assuming that the population distribution of abilities follows a standard normal distribution. For the M2PL model, we applied the second constraint method proposed by Béguin and Glas (2001). This method ensures identifiability by constraining the item parameters as follows: the first *Q* items’ intercept parameters 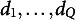 are set to zero, and the discrimination parameter 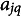 is set to 1 when 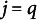 and to 0 when 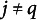 , for each 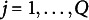 and 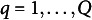 . Meanwhile, the ability parameters are freely estimated.

### 5.3 Parameter settings and initialization values

In the SNA, several parameters require adjustment, including 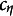 , 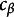 , 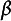 , and the step size parameter 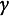 . Among these, the step size parameter is the most critical, as it directly controls the magnitude of each update and significantly influences the algorithm’s convergence speed and stability. A step size that is too large may lead to unstable convergence or even divergence, while a step size that is too small can result in slow convergence or becoming trapped in a local optimum. Clearly, the choice of step size impacts both the update direction and magnitude, thereby affecting the accuracy of the final solution and the speed of convergence. To simplify the parameter tuning process and reduce complexity, most parameters were fixed, allowing the primary focus to remain on step size adjustment, which enhances the efficiency and practicality of simulations. Specifically, following Boyer and Godichon-Baggioni (2023), we set 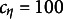 . Based on Bercu et al. (2020), we set 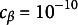 and 
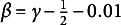
 . Additionally, we conducted simulation studies with different values of the tuning parameters ( 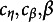 ) to systematically assess their impact on the algorithm’s performance. The results indicate that the current configuration of these parameters performs well in maintaining stable and satisfactory estimation accuracy and convergence speed, thereby validating their rationality and robustness. The detailed simulation results are provided in Section A.2 of the Supplementary Material.

In addition, we initialized all estimates of the discrimination parameter to 1 and the difficulty/intercept parameter to 0. This approach simplifies the algorithm’s initialization process and helps address convergence difficulties caused by excessively high or low initial estimates during iterations. To take advantage of the numerical efficiency of the Gauss–Hermite quadrature method for approximate integration, we set the initial Hessian matrix value as 

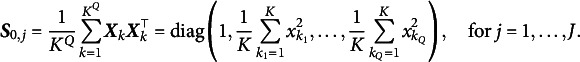

This approach addresses common numerical instability issues and provides a robust foundation for subsequent iterations of the algorithm. While this initialization was chosen, it is equally feasible to initialize the Hessian matrix as the identity matrix, provided it ensures numerical stability and avoids singular matrices, which may lead to computational failures. It is worth noting that, in both the simulation and the subsequent empirical study, the batch size used in our algorithm consistently corresponds to the sample size of a single examinee, with parameter updates occurring after observing each individual’s response data.

### 5.4 Evaluation criteria

To evaluate parameter recovery, the bias and RMSE of the item parameter estimates and ability parameter estimates are calculated. Computational efficiency is assessed based on the average running time of each replication. The following formulas were calculated: 
(34)

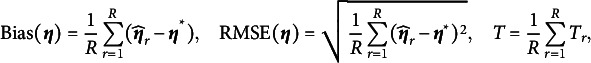

where *R* represents the number of replications, 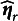 denotes the parameter estimate from the *r*th replication, 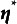 indicates the true value of the parameter, and 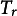 represents the running time of the *r*th replication. The estimation of ability parameters is carried out as follows: first, the item parameters estimated using the mirt package are treated as known values. Then, the EAP method is applied to estimate 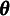 . These estimates are subsequently compared with the real-time ability parameter estimates obtained from the two online algorithms.

### 5.5 Result analysis

#### 5.5.1 Analysis of parameter recovery results

Tables 1–3 present the RMSEs of the model parameter estimates for three different latent dimensions when the number of items is 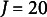 . The results for 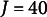 and 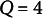 can be found in Tables A1–A4 in the Supplementary Material. From Tables 1–3 and Tables A1–A4 in the Supplementary Material, we can observe the impact of different factors on parameter estimation.

**Table 1:** Average RMSEs for the 2PL model parameters with 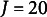 across TSNA, TASNA, and EM algorithm implemented in the mirt package

*J*	*N*	*K*	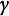	***a***			***b***			**θ**		
				TSNA	TASNA	mirt	TSNA	TASNA	mirt	TSNA	TASNA	mirt
	5,000	10	0.65	0.142	**0.056**	0.051	0.190	**0.067**	0.061	0.422	0.419	0.416
			0.75	**0.093**	**0.055**		0.117	**0.068**		0.419	0.420	
			0.90	**0.069**	0.128		**0.086**	0.110		0.420	0.423	
			1.00	0.130	0.206		0.105	0.122		0.423	0.427	
		20	0.65	0.146	**0.066**		0.188	**0.069**		0.420	0.417	0.415
			0.75	**0.095**	**0.052**		0.117	**0.067**		0.418	0.418	
			0.90	**0.065**	0.124		**0.084**	0.110		0.419	0.422	
			1.00	0.127	0.203		0.105	0.123		0.422	0.426	
20	10,000	10	0.65	0.113	**0.040**	0.037	0.175	**0.049**	0.043	0.422	0.421	0.419
			0.75	**0.073**	**0.039**		0.106	**0.048**		0.421	0.421	
			0.90	**0.052**	0.090		**0.064**	0.078		0.422	0.424	
			1.00	0.104	0.168		0.086	0.107		0.424	0.427	
		20	0.65	0.112	**0.049**		0.173	**0.052**		0.420	0.419	0.417
			0.75	**0.071**	**0.038**		0.104	**0.049**		0.419	0.419	
			0.90	**0.047**	0.086		**0.062**	0.077		0.420	0.423	
			1.00	0.100	0.166		0.085	0.109		0.423	0.426	
	20,000	10	0.65	0.091	**0.028**	0.024	0.146	**0.036**	0.034	0.422	0.421	0.419
			0.75	**0.056**	**0.026**		0.082	**0.037**		0.421	0.421	
			0.90	**0.034**	0.063		**0.049**	0.064		0.421	0.423	
			1.00	0.079	0.137		0.077	0.105		0.424	0.426	
		20	0.65	0.091	**0.038**		0.144	**0.039**		0.420	0.419	0.418
			0.75	**0.056**	**0.026**		0.081	**0.037**		0.419	0.419	
			0.90	**0.029**	0.057		**0.047**	0.062		0.420	0.421	
			1.00	0.075	0.134		0.076	0.106		0.422	0.425	

*Note*: Boldface values indicate estimation results with relatively smaller RMSEs under the same simulation condition. In some settings, more than one tuning parameter value shows comparable performance and is therefore highlighted.

**Table 2:** Average RMSEs for the M2PL model parameters with 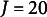 , 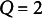 across TSNA, TASNA, and EM algorithm implemented in the mirt package

*K*	*N*	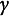	***a***_1_			***a***_2_			***d***			**θ**		
			TSNA	TASNA	mirt	TSNA	TASNA	mirt	TSNA	TASNA	mirt	TSNA	TASNA	mirt
	5,000	0.65	0.157	**0.082**	0.068	0.152	**0.079**	0.071	0.127	**0.057**	0.045	0.588	0.586	0.578
		0.75	0.100	**0.072**		0.099	**0.078**		0.086	**0.083**		0.585	0.589	
		0.90	0.087	0.175		0.108	0.183		0.111	0.174		0.591	0.604	
		1.00	0.199	0.285		0.212	0.280		0.188	0.280		0.608	0.627	
10	10,000	0.65	0.115	**0.064**	0.048	0.116	**0.069**	0.051	0.097	**0.032**	0.032	0.588	0.585	0.580
		0.75	**0.075**	**0.054**		**0.074**	**0.050**		**0.063**	**0.041**		0.585	0.586	
		0.90	**0.059**	0.125		**0.065**	0.124		**0.075**	0.121		0.587	0.595	
		1.00	0.160	0.241		0.159	0.227		0.150	0.225		0.599	0.613	
	20,000	0.65	0.092	**0.046**	0.034	0.093	**0.044**	0.034	0.076	**0.024**	0.024	0.586	0.584	0.581
		0.75	**0.060**	**0.037**		**0.057**	**0.036**		**0.047**	**0.027**		0.584	0.585	
		0.90	**0.038**	0.085		**0.045**	0.093		**0.066**	0.102		0.586	0.591	
		1.00	0.125	0.200		0.133	0.199		0.137	0.192		0.594	0.606	
	5,000	0.65	0.159	**0.085**	0.068	0.153	**0.081**	0.071	0.127	**0.057**	0.045	0.588	0.586	0.577
		0.75	0.100	**0.071**		0.100	**0.077**		0.086	**0.083**		0.585	0.589	
		0.90	0.085	0.173		0.106	0.181		0.112	0.174		0.591	0.604	
		1.00	0.197	0.283		0.210	0.278		0.188	0.280		0.608	0.626	
20	10,000	0.65	0.115	**0.066**	0.048	0.116	**0.071**	0.051	0.096	**0.032**	0.032	0.588	0.585	0.580
		0.75	**0.075**	**0.054**		**0.074**	**0.051**		**0.063**	**0.041**		0.585	0.586	
		0.90	**0.058**	0.124		**0.063**	0.122		**0.075**	0.121		0.587	0.595	
		1.00	0.158	0.240		0.158	0.225		0.150	0.225		0.599	0.613	
	20,000	0.65	0.093	**0.049**	0.034	0.093	**0.045**	0.034	0.076	**0.024**	0.024	0.586	0.584	0.581
		0.75	**0.061**	**0.038**		**0.057**	**0.035**		**0.047**	**0.027**		0.584	0.585	
		0.90	**0.038**	0.084		**0.044**	0.091		**0.065**	0.101		0.585	0.591	
		1.00	0.123	0.198		0.131	0.197		0.137	0.192		0.594	0.605	

*Note*: Boldface values indicate estimation results with relatively smaller RMSEs under the same simulation condition. In some settings, more than one tuning parameter value shows comparable performance and is therefore highlighted.

**Table 3:** Average RMSEs for the M2PL model parameters with 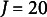 , 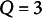 across TSNA, TASNA, and EM algorithm implemented in the mirt package

*K*	*N*	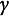	***a***_1_			***a***_2_			***a***_3_			***d***		
			TSNA	TASNA	mirt	TSNA	TASNA	mirt	TSNA	TASNA	mirt	TSNA	TASNA	mirt
	5,000	0.65	0.172	0.095	0.086	0.179	0.117	0.091	0.153	0.080	0.096	0.127	0.063	0.056
		0.75	0.108	0.101		0.126	0.107		0.098	0.111		0.092	0.087	
		0.90	0.132	0.231		0.140	0.213		0.159	0.240		0.102	0.169	
		1.00	0.253	0.332		0.247	0.308		0.255	0.324		0.170	0.266	
10	10,000	0.65	0.125	**0.073**	0.065	0.122	0.101	0.062	0.120	**0.060**	0.059	0.104	**0.049**	0.037
		0.75	**0.082**	**0.069**		**0.089**	**0.081**		**0.091**	**0.088**		**0.070**	**0.063**	
		0.90	**0.098**	0.176		**0.090**	0.150		0.136	0.201		**0.074**	0.130	
		1.00	0.215	0.289		0.196	0.259		0.231	0.295		0.135	0.216	
	20,000	0.65	0.098	**0.054**	0.041	0.105	**0.073**	0.045	0.100	**0.045**	0.042	0.085	**0.029**	0.024
		0.75	**0.064**	**0.048**		**0.080**	**0.066**		**0.067**	**0.065**		**0.053**	**0.038**	
		0.90	**0.070**	0.135		**0.071**	0.119		0.109	0.168		**0.052**	0.095	
		1.00	0.184	0.255		0.172	0.236		0.207	0.269		0.109	0.176	
	5,000	0.65	0.174	0.096	0.086	0.181	0.120	0.091	0.154	0.081	0.096	0.127	0.063	0.056
		0.75	0.109	0.100		0.128	0.107		0.098	0.110		0.091	0.086	
		0.90	0.131	0.229		0.139	0.212		0.157	0.238		0.102	0.169	
		1.00	0.251	0.331		0.246	0.306		0.253	0.322		0.170	0.266	
20	10,000	0.65	0.125	**0.075**	0.065	0.122	0.102	0.062	0.121	**0.060**	0.059	0.104	**0.049**	0.037
		0.75	**0.082**	**0.068**		**0.090**	**0.082**		**0.091**	**0.087**		**0.070**	**0.064**	
		0.90	**0.097**	0.174		**0.089**	0.149		0.134	0.199		**0.075**	0.130	
		1.00	0.213	0.287		0.195	0.257		0.229	0.294		0.135	0.217	
	20,000	0.65	0.099	**0.055**	0.041	0.106	**0.075**	0.045	0.100	**0.045**	0.042	0.085	**0.029**	0.024
		0.75	**0.064**	**0.048**		**0.081**	**0.067**		**0.066**	**0.064**		**0.053**	**0.038**	
		0.90	**0.069**	0.134		**0.072**	0.118		0.108	0.166		**0.052**	0.095	
		1.00	0.182	0.254		0.171	0.234		0.205	0.268		0.109	0.176	

*Note*: Boldface values indicate estimation results with relatively smaller RMSEs under the same simulation condition. In some settings, more than one tuning parameter value shows comparable performance and is therefore highlighted.

First, as the sample size increases, the two proposed online algorithms and the EM algorithm show improved parameter estimation accuracy across different latent dimensions. However, as the latent dimensionality increases, the performance of the EM algorithm deteriorates significantly, while the two online algorithms (TASNA and TSNA) exhibit relatively stable performance under various settings. Specifically, for latent dimensions 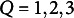 , the EM algorithm with fixed quadrature constraints achieves the best overall accuracy, and both TASNA with 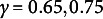 and TSNA with 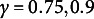 yield comparable estimation results. However, as shown in Tables A3 and A4 in the Supplementary Material, when the latent dimension increases to 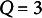 and 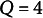 , the EM algorithm’s RMSE for parameter estimation exceeds 0.1 in the case of 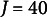 items, while TASNA with 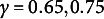 and TSNA with 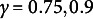 maintain lower RMSE values, significantly outperforming the EM algorithm.

Secondly, for fixed sample sizes and item numbers, the optimal value of 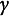 largely depends on the specific online algorithm used. In the TASNA algorithm, 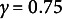 and 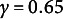 yield better outcomes, with 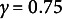 generally outperforming 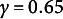 across various conditions. Under these configurations, TASNA not only achieves estimation accuracy comparable to the EM algorithm, but also converges faster and more stably, even with relatively small sample sizes. In contrast, for the TSNA algorithm, 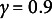 and 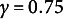 typically lead to better estimation results, with 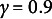 performing best in most scenarios. Overall, TASNA tends to achieve higher estimation accuracy than TSNA. As the sample size increases, TSNA with 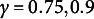 gradually reaches estimation performance comparable to TASNA with 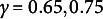 and the EM algorithm. The poorest performance is observed for TASNA when 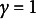 , with noticeably higher estimation errors and slower convergence. This result aligns with the theoretical analysis, which suggests that TASNA performs better in terms of stability and convergence when 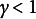 , while setting 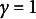 may lead to excessive variance accumulation and degraded estimation accuracy.

Moreover, given a fixed sample size, the effectiveness of the quadrature node setting (*K*) is primarily influenced by the number of items. Simulation results show that when the number of items is small (e.g., 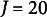 ), the estimation accuracy achieved with 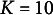 and 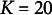 is nearly identical. This suggests that, with a relatively small number of items, increasing the number of quadrature nodes does not significantly improve estimation performance and instead results in additional computational cost. As the number of items increases, the number of parameters to be estimated also grows, and in such cases, increasing *K* can enhance estimation accuracy. However, the improvement from 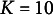 to 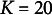 is considerably smaller than the improvement achieved by increasing the sample size. Furthermore, we observed that in most settings, the accuracy obtained with 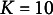 remains within an acceptable range. Therefore, from the perspective of balancing accuracy and computational efficiency, 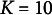 is generally the most cost-effective choice.

In addition, regarding the recovery of the ability parameter 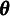 , both stochastic algorithms show very similar estimation results across different 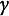 settings, with the estimates close to the EAP estimation of 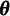 , as presented in Tables 1 and 2 and Tables A1, A2, A6, and A7 in the Supplementary Material. The recovery results for each method improve as the number of items *J* increases. Finally, it is worth noting that, under the four-dimensional ( 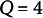 ) setting, due to increased model complexity and the “curse of dimensionality,” the computational burden of numerical integration rises sharply when the number of Gauss–Hermite quadrature nodes is set to 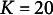 . This results in a substantial increase in total running time, even surpassing that of the mirt package under the same conditions. Considering both simulation efficiency and computational resource constraints, we ultimately decided not to run simulations for 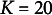 in this setting to avoid unnecessary computational costs. However, from Tables A4 and A5 in the Supplementary Material, we found that with 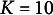 , the proposed algorithms not only demonstrate desirable computational efficiency but also achieve significantly better estimation accuracy compared to mirt. Therefore, even in a four-dimensional setting, the proposed methods remain highly applicable and competitive.

#### 5.5.2 Real-time evaluation of parameter estimation accuracy

**Figure 1:**
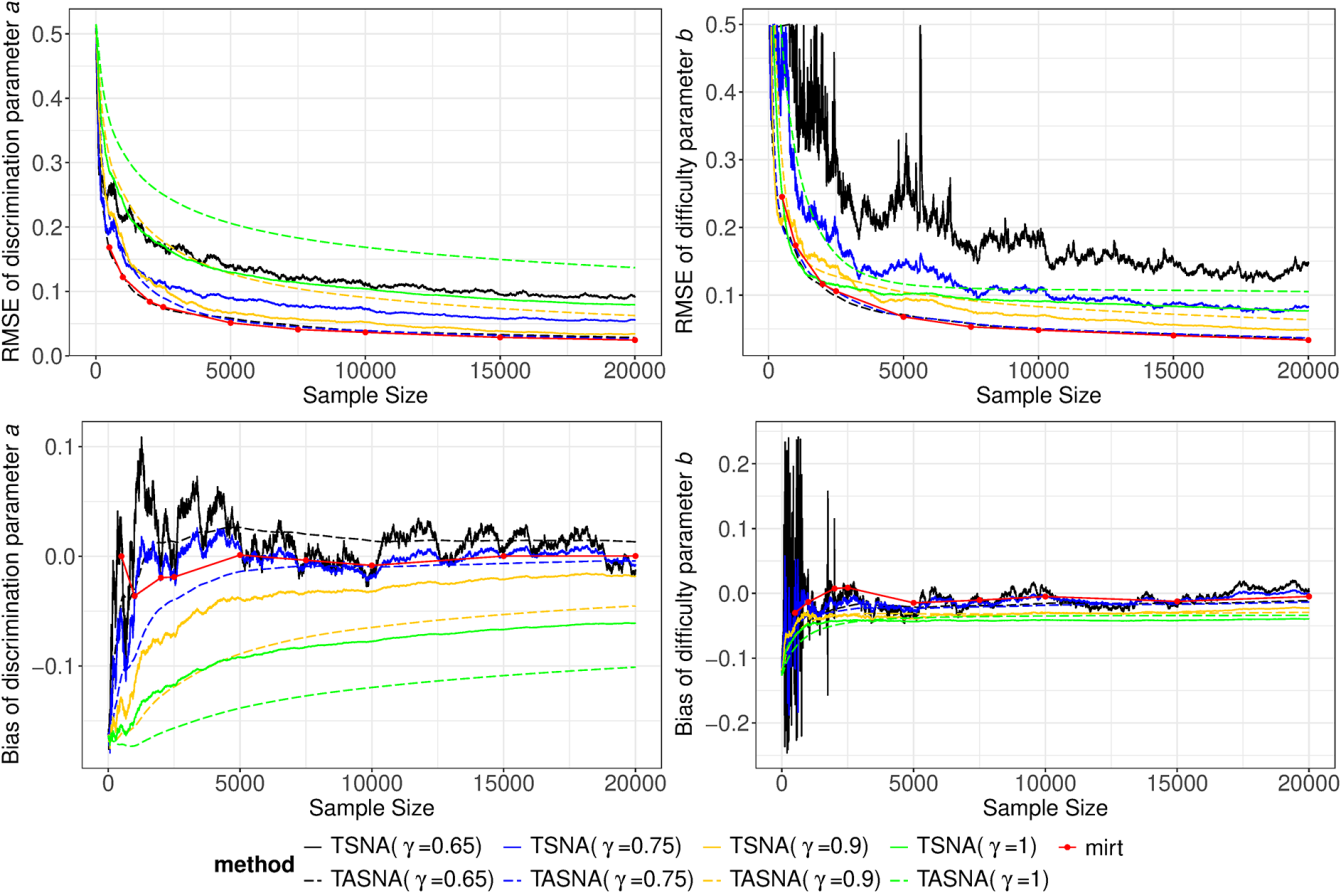
Bias and RMSE of real-time estimates of item parameters in the 2PL model across TSNA, TASNA, and EM algorithm for different step sizes with 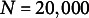 , 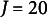 , and 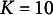 .

**Figure 2:**
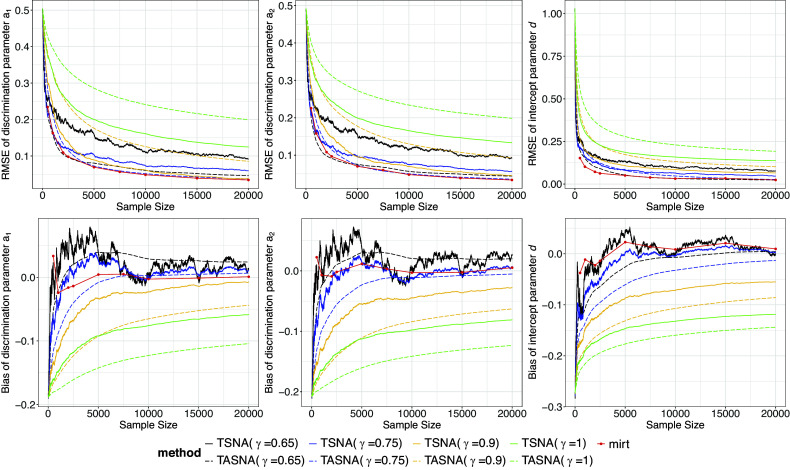
Bias and RMSE of real-time estimates of item parameters in the M2PL model with 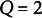 across TSNA, TASNA, and EM algorithm for different step sizes with 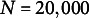 , 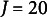 , and 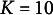 .

Figures 1 and 2, respectively, show the real-time estimation results of item parameters for the 2PL model and the M2PL model with 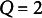 under the conditions of 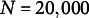 , 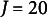 , and 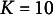 . Considering that the EM algorithm is not an online method, to facilitate comparison between the proposed method and the traditional EM algorithm across various sample sizes, the figure presents the estimation results of the EM algorithm at multiple discrete sample points ( 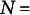 500, 1,000, 2,000, 2,500, 5,000, 7,500, 10,000, 15,000, and 20,000). The top two subplots in Figures 1 and 2 display the RMSEs of the real-time estimates for the item parameters. The plots reveal that as the sample size increases, the RMSEs for all 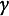 settings decrease and converge rapidly. For example, when the sample size is 2,500, the RMSE of the TASNA with 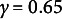 and 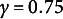 has already decreased to approximately 0.1. When the sample size reaches 5,000, the RMSE further reduces to a range of around 0.050 to 0.075. Additionally, the TASNA demonstrates smoother and more stable performance compared to the TSNA, which lacks an averaging step and exhibits overall higher fluctuations, especially with smaller 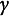 . Furthermore, the TASNA with 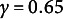 and 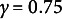 performs the best, closely aligning with those from the EM algorithm, which is consistent with the findings presented in Tables 1–3. This is followed by the TSNA with 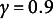 and 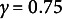 . It is worth noting that, in small sample scenarios (e.g., 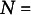 500, 1,000, and 2,000), the TASNA method with 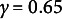 and 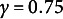 achieves accuracy nearly equivalent to that of the EM algorithm. In contrast, the TASNA with 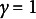 yields the worst performance for the discrimination parameters, while the TSNA with 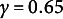 shows the worst performance for the difficulty/intercept parameters, exhibiting substantial instability. The lower subplots in Figures 1 and 2 present the bias of the item parameter estimates, which align with the RMSE results. The bias of TASNA with 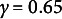 and 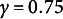 remains very stable and close to zero, whereas the bias of TSNA under the same 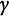 settings fluctuates more significantly. These findings highlight the advantages of TASNA, particularly with 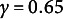 and 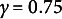 , in achieving accurate and stable parameter estimates under the given conditions.

Additionally, the real-time estimation results for the item parameters under other conditions are provided in Figures A1–A6 in the Supplementary Material. These results are consistent with those in Figures 1 and 2, further confirming the robustness and generalizability of the findings across different conditions.

#### 5.5.3 Comparative analysis of running time

**Figure 3:**
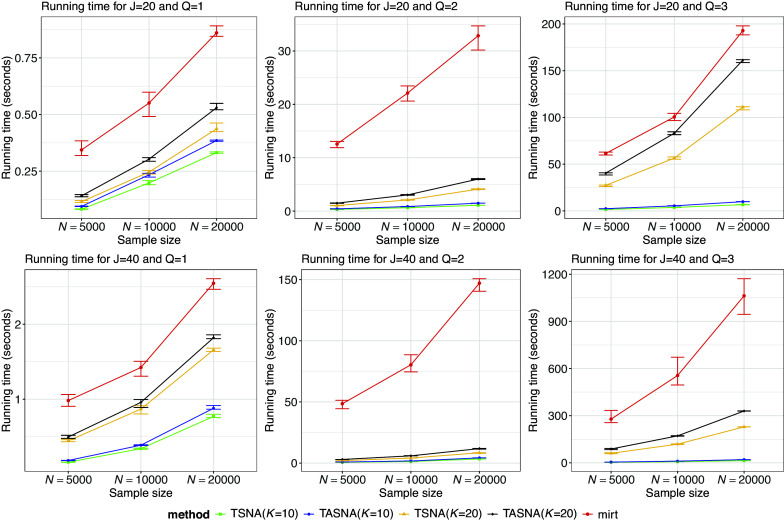
Running time for the TSNA, TASNA, and EM algorithms across 50 simulation replications, displayed as median values with error bars representing the 25th and 75th percentiles.

Figure 3 presents the average running time of the three estimation methods for all conditions with a fixed 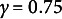 . The results clearly indicate that the computation time of the proposed online algorithms is significantly faster than the EM algorithm implemented in the mirt package. Among the online algorithms, TSNA is slightly faster than TASNA, as it does not include the averaging step, while the EM algorithm is the slowest overall. Furthermore, the number of quadrature nodes (*K*) affects the computation time of the online algorithm: increasing the number of nodes leads to slower computation time, which is particularly noticeable in the M2PL model ( 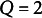 and 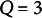 ). However, even with a higher number of nodes, the online algorithm remains faster than the mirt package. Therefore, our method consistently maintains short computation time with acceptable estimation error for small and medium sample sizes, and demonstrates a smoother, more predictable increase in running time. As sample sizes and item numbers grow, and as the latent trait dimension increases to 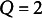 and 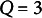 , its advantages in computational efficiency and stability become even more pronounced. In contrast, the computational cost of the mirt method rises significantly with larger sample sizes and higher dimensionality, particularly in high-dimensional contexts.

For instance, under the three-dimensional setting, with 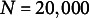 and 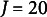 , the computation time for both online algorithms is approximately 10 seconds when 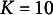 , whereas the mirt package takes nearly 200 seconds under the same conditions. Although running time increases when using 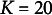 , it remains shorter than that of mirt. Given that estimation accuracy is nearly identical for 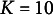 and 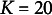 in this setting, using 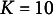 offers a clear advantage in both accuracy and efficiency when 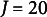 . This trend becomes even more pronounced as the number of items increases to 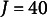 : with 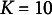 , the online algorithms take about 20 seconds, while the mirt package requires around 1,000 seconds under the same conditions. Even with 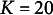 , the online methods take only about 300 seconds—still much shorter than mirt. Therefore, TSNA and TASNA demonstrate superior computational stability and scalability as sample size and model complexity increase. These results highlight the remarkable computational efficiency of the proposed online algorithms, particularly in large-scale applications. Please refer to Table A5 in the Supplementary Material for detailed average running time.

#### 5.5.4 Evaluation of standard errors for parameter estimates

To evaluate the standard errors of the parameter estimates proposed in Remark 2 of Section 4, we conducted a small-scale additional simulation study. The simulation was carried out with a fixed sample size ( 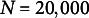 ), item numbers ( 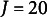 and 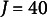 ), and latent dimensions ( 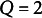 and 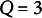 ). The ranges of standard errors for the item parameter estimates were reported across different step-size parameters ( 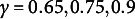 , and 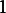 ), as shown in Tables A8 and A9 in the Supplementary Material. All other simulation settings were consistent with those used in the main simulation study. The results show that when the discrimination parameters follow a uniform distribution 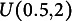 and the intercept parameters are drawn from a standard normal distribution 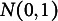 , the standard errors of the item parameters remain stable within the range of 0.015 to 0.045, exhibiting low variability and stable distributions. These findings indicate that the proposed standard error estimates demonstrate good numerical stability and reliability.

Overall, the proposed algorithm demonstrates superior performance in both parameter recovery and computational efficiency. Specifically, when the number of items is small (e.g., 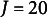 ), choosing a smaller number of quadrature nodes (e.g., 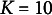 ) combined with TASNA using 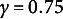 or 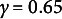 typically achieves a desirable balance between estimation accuracy and computational efficiency. In scenarios with a larger number of items, we recommend tailoring the strategy based on practical needs: if higher estimation accuracy is required and sufficient computational resources are available, increasing the number of quadrature nodes (e.g., 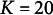 ) will enhance estimation precision, while using stable step-size settings (e.g., TSNA with 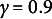 or 0.75, or TASNA with 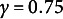 or 0.65); if computational efficiency is the primary concern, combining 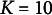 with TASNA ( 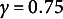 or 0.65) or TSNA ( 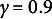 or 0.75) is recommended to achieve a balance between accuracy and running time. Based on the current simulation results, this recommendation has shown good generalizability and robustness across most model complexities and sample sizes.

## 6 Empirical example

### 6.1 Data description

In this section, real data from the TIMSS were analyzed to evaluate the performance of the proposed TASNA. TIMSS (Martin et al., 2020; Mullis & Martin, 2017) is a well-established international assessment of mathematics and science achievement for fourth- and eighth-grade students conducted every four years. The math and science item pools at each grade level are divided into item blocks, which are assembled into 14 distinct student achievement booklets. Each student completes one booklet. We selected items identical to those used by Cui et al. (2024), which include one mathematics block and one science block from the eighth-grade Booklets 5 and 6. These blocks contain a total of 28 items, comprising 13 mathematics items and 15 science items. The specific item codes are provided in Appendix C of Cui et al. (2024). After excluding students with missing responses, the final sample size was 9,874. The mathematics block contained two polytomous items, while the science block included three polytomous items, all with a maximum score of 2. To simplify the analysis, the polytomous items were treated as dichotomous by recoding scores of 2 as correct (i.e., 1) and scores below 2 as incorrect (i.e., 0). This recoding ensures consistency with the scoring framework used for dichotomous items in the analysis.

### 6.2 Analysis of empirical data results fitted with the unidimensional 2PL model

In the operational analysis of TIMSS data, the unidimensional 2PL IRT model was applied separately to the mathematics and science domains. Cui et al. (2024) used their proposed modified information criterion and demonstrated that both domains are essentially unidimensional when analyzed independently. Thus, we first present the results under a unidimensional framework. Consistent with the simulation studies, the estimation methods include the EM algorithm with fixed quadrature (implemented in the mirt package), the TASNA, and the TSNA. Previous simulation results indicate that when the number of items is fewer than 40, TASNA with step size parameters 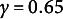 and 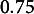 , as well as TSNA with 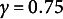 and 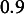 , provides better parameter recovery. While the performance of these methods is comparable when the number of quadrature nodes is 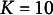 or 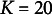 , the computation time is considerably shorter for 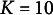 . Therefore, we fixed 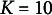 and selected TASNA with 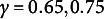 and TSNA with 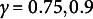 . All other parameter settings are consistent with those used in the simulation studies. Furthermore, to address scale indeterminacy, we assumed that the covariance matrix of the latent traits is an identity matrix, following the approach used by Cui et al. (2024). This assumption standardizes the scale of the latent variables, ensuring consistency across analyses.

**Figure 4:**
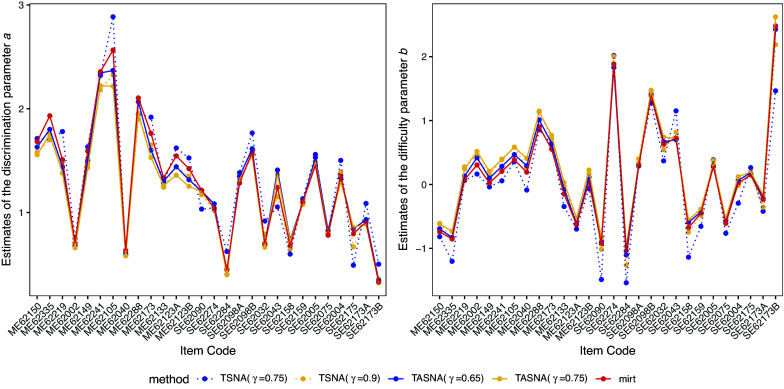
Estimates of item parameters for the 2PL model across TSNA, TASNA, and EM algorithm with corresponding step sizes.

Figure 4 shows the parameter estimates for discrimination ( 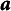 ) and difficulty ( 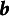 ) obtained using the EM algorithm and the SNAs under the 2PL model. The results indicate that the estimates from both methods are quite similar. However, for some items, the EM algorithm tends to produce slightly higher estimates than the SNAs. Notably, the TSNA method with 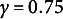 is more sensitive to extreme parameter values. This happens because, as parameter values become very large, the gradient of the objective function can also grow disproportionately. With 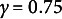 , the step size also becomes relatively large, causing each update to be large magnitude. This, in turn, can lead to “jumps” in the parameter estimates that may overshoot the true optimal point of the objective function, resulting in inflated parameter estimates. In contrast, TASNA reduces the influence of extreme outliers and noise by averaging information across all iterations. This results in more stable updates, helping the algorithm converge more consistently toward the optimal solution, and ultimately providing more accurate and reliable parameter estimates across different conditions.

To demonstrate the real-time estimation performance of the algorithm, we present the real-time trajectory plots of discrimination parameter estimation in Figure D1 in the Supplementary Material and difficulty parameter estimation in Figure D2 in the Supplementary Material. These plots show that the parameter estimates obtained using the TASNA demonstrate more stable convergence, with no extreme values, even in the initial stages of estimation. In contrast, Figure D2 in the Supplementary Material shows that the TSNA method exhibits more noticeable fluctuations, particularly for some items in the difficulty parameter 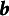 (e.g., ME62002, SE62284, and SE62173B), where unusually large estimates are observed. These findings highlight the necessity of including the averaging step in the algorithm. For large-scale educational testing scenarios that require real-time estimation, the TASNA is notably more stable and accurate compared to the TSNA. Moreover, TASNA effectively avoids extreme parameter estimates, making it a more practical and reliable choice for real-time applications.

### 6.3 Analysis of empirical data results fitted with the multidimensional 2PL model

To investigate the relationship between latent abilities in different domains, we adopted the M2PL model to estimate all 28 items in the TIMSS dataset, assuming two latent dimensions ( 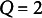 ). The mathematics proficiency and science proficiency are conceptually distinct, each measuring different cognitive skills. Mathematics proficiency primarily involves numerical reasoning, algebraic thinking, and problem-solving skills, while science proficiency focuses on understanding scientific principles, experimental design, and analytical reasoning. These differences suggest that mathematics and science abilities should be treated as separate latent traits. The modified information criterion introduced by Cui et al. (2024) further supports this distinction, showing that a two-dimensional latent structure provides a significantly better fit compared to a unidimensional model. This finding aligns with the inherent design characteristics of the TIMSS data, where the items are specifically developed to assess the distinct domains of mathematics and science proficiency. To ensure model identifiability, we adopt the constraints suggested by Béguin and Glas (2001). For the first item in the math domain, we constrain the discrimination parameters as 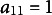 and 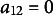 , with the corresponding intercept parameter set to 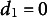 . Similarly, for the first item in the science domain, we constrain the discrimination parameters as 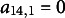 and 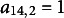 , with the corresponding intercept parameter set to 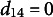 .

**Figure 5:**
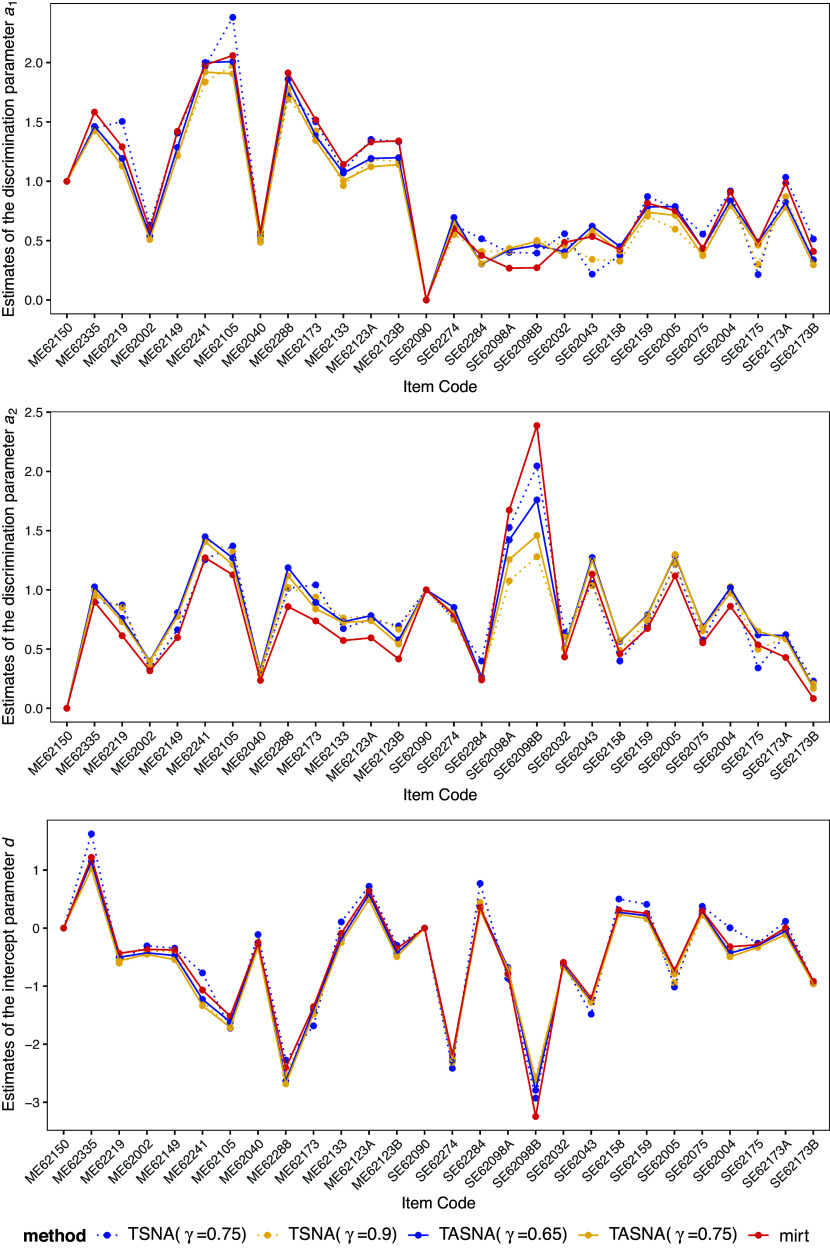
Estimates of item parameters for the M2PL model across TSNA, TASNA, and EM algorithm with corresponding step sizes.

Figure 5 presents the parameter estimation results for both the SNA and the EM algorithm in the M2PL model. As observed in Cui et al. (2024), the estimated parameters reveal a clear bi-factor loading structure. The first plot in Figure 5 illustrates the first dimension of the loadings parameter (i.e., the discrimination parameters 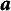 ), which primarily reflects students’ mathematical problem-solving ability. The second plot in Figure 5 depicts the second dimension of the loadings, which predominantly measures students’ science ability. These two dimensions represent distinct but correlated aspects of overall cognitive ability, capturing the unique contributions of mathematics and science proficiencies. The third plot in Figure 5 demonstrates that the intercept parameters 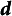 estimated using both the SNA and the EM algorithm are very similar. Overall, the estimated factor structure aligns with existing literature, supporting the validity of the proposed method. These results suggest that the SNA is a reliable and efficient tool for analyzing large-scale assessment data, making it well-suited for real-time or near-real-time educational assessment applications.

## 7 Conclusion

With technological advancements, educational assessment is transforming from static, periodic data analysis toward real-time, dynamic data analysis. The widespread application of real-time data has significantly enhanced the scientific rigor and efficiency of educational management. It also supports personalized learning, dynamic assessments, and promotes educational equity. However, this shift brings new challenges, requiring innovative strategies to fully leverage real-time data for creating more equitable, efficient, and adaptive educational systems.

Existing methods for estimating IRT parameters are primarily designed for offline testing environments and are often difficult to apply directly to real-time or near-real-time assessments. To address this limitation, this article proposes a TASNA to handle large-scale online response data in educational measurement. When examinee response data arrive in real time, the proposed algorithm dynamically updates both item and ability parameters within the IRT model, thereby enhancing the accuracy of personalized assessments and providing robust technical support for intelligent educational systems. In addition to its real-time applications, TASNA also performs well in offline settings, particularly for large-scale datasets involving a substantial number of examinees and test items. As the sample size increases, the accuracy of parameter estimates improves, and TASNA shows significant computational efficiency advantages over traditional EM algorithms, which makes TASNA a powerful alternative to offline EM methods when faster approximation is required. In addition, we investigate the theoretical properties of the TASNA, and the almost sure convergence and asymptotic normality are rigorously proven, ensuring the reliability, efficiency, and accuracy of the results. The effectiveness and practicality of the TASNA are validated through simulation studies and real data analysis. This study not only advances educational measurement by moving beyond traditional offline analysis to real-time analysis, addressing the needs of dynamic and adaptive assessments, but also overcomes the limitations of traditional IRT methods in handling large-scale and real-time data. These contributions promote the modernization and further theoretical development of IRT, broadening its potential for application in intelligent educational systems.

This study has several limitations. First, the SNA performs best with large sample sizes and tends to be less effective with smaller samples. In cases with limited data, the algorithm may struggle to converge and may fall into a local optimum. However, as the sample size increases, the accuracy of the parameter estimates improves significantly, highlighting its suitability for large-scale data. Second, the SNA is highly sensitive to parameterization, particularly the step size. Improper parameter settings can impact convergence, stability, and accuracy, making careful tuning and optimization of these parameters essential for effective application. In this study, we focused on the impact of different 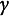 values on the standard errors in the small-scale additional simulation. However, the effects of other tuning constants (e.g., 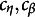 , and 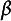 ) will be addressed in future research to further investigate the robustness of standard error estimation.

Additionally, the current version of TASNA has limitation in handling high-dimensional latent traits. Similar to the EM algorithm, TASNA employs Gauss–Hermite quadrature techniques to approximate the integral of ability parameters, which leads to increased computational complexity as the dimensionality of the latent traits grows. Although, in both three- and four-dimensional settings, the proposed TSNA and TASNA algorithms maintain strong estimation accuracy and significant computational advantages, we also acknowledge that in settings with five or more dimensions, the current quadrature-based integration strategy may face more serious efficiency bottlenecks. Therefore, future research could explore alternative integral approximation methods or combine existing approaches, such as variational methods or Monte Carlo-based approximation methods, with the SNA to extend its use to high-dimensional latent structures. Another potential direction for future work is to adapt the recursive SNA to polytomous response models, where the response variables take multiple ordered categories. Extending the algorithm to these models involves working with a more complex parameter space and may require accommodating multiple latent variables. Advancing research in this direction will contribute to more sophisticated educational assessments and support the design of a personalized learning system.

Moreover, since the proposed algorithm optimizes a numerically approximated objective function 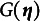 instead of the exact marginal log-likelihood, the numerical integration error introduced by the Gauss–Hermite quadrature is treated as a negligible term in the asymptotic analysis. This assumption is theoretically sound when *K* is sufficiently large, ensuring that the approximation error remains asymptotically small and does not significantly impact the results. However, it is important to recognize that the theoretical results established in this article—such as the consistency and asymptotic normality of the parameter estimates—are derived under the assumption of negligible numerical error. In practice, when *K* is moderate or small, this approximation may introduce bias. Although this limitation is inherent to numerical methods and cannot be entirely avoided, future work could investigate the impact of finite *K* on the asymptotic properties of the estimators and explore other strategies to balance computational efficiency with theoretical precision.

To better demonstrate the practical value and potential applicability of the proposed algorithm, we explored two possible application scenarios within the framework of IRT models, aiming to provide practice-oriented insights for future research. First, the online estimation algorithm proposed in this study can be applied to online item calibration in computerized adaptive testing (CAT). In large-scale testing platforms, traditional offline calibration methods often rely on substantial pretest data, making it difficult to meet the demand for the rapid introduction of new items. In contrast, our proposed algorithm offers significant advantages for online updating: as examinees progressively complete tests and submit response data, the algorithm dynamically adjusts the estimates of item discrimination and difficulty/intercept, enabling efficient, low-latency online calibration. Particularly in scenarios where new items are randomly or adaptively assigned to examinees and responses are collected in real time, the algorithm updates parameter estimates immediately upon receiving each new data point. This eliminates the need for the large sample accumulation required by traditional methods, greatly improving the efficiency and real-time performance of introducing and calibrating new items.

Second, the proposed algorithm can also be considered for use in item-based adaptive learning systems to enable real-time parameter estimation of both item and learner parameters. In recent years, online learning systems have attracted growing attention in both academia and industry due to their advantage of delivering personalized content dynamically according to each learner’s needs. Unlike traditional CAT environments, online learning platforms generally exhibit two characteristics: (1) they often involve large item pools, many of which have not undergone expensive field calibration and (2) learners’ abilities evolve continuously throughout the learning process. Jiang et al. (2023) utilized a moment-matching Bayesian updating algorithm proposed by Weng and Coad (2018) to estimate item and learner parameters in real time, in combination with a modified maximum posterior weighted information (MPWI) criterion to enable personalized item allocation. Building on this, our proposed algorithm may serve as an effective alternative to the moment-matching Bayesian updating approach, offering stronger theoretical guarantees and faster convergence. This, in turn, may enhance the responsiveness and estimation accuracy of adaptive learning systems and further improve the effectiveness of personalized instruction.

Overall, while the applications discussed above are still at the conceptual validation stage and have not yet been widely deployed in large-scale practice, we believe that the proposed online algorithm holds significant research value and broad practical potential within the framework of IRT models. Future research may expand on this work in several ways—for example, by conducting systematic theoretical analyses to strengthen the algorithm’s statistical foundation, performing large-scale simulation studies to assess its robustness and accuracy across diverse testing environments, and carrying out empirical validation using real-world data from online assessment or learning platforms. These efforts will collectively enhance the feasibility and practical utility of the algorithm in IRT applications.

## Supporting information

10.1017/psy.2025.10064.sm001 Xu et al. supplementary material Xu et al. supplementary material

## Data Availability

The real dataset was derived from the following resources available in the public domain: https://timssandpirls.bc.edu/databases-landing.html.

## Supplementary material

The supplementary material for this article can be found at https://doi.org/10.1017/psy.2025.10064.

## References

[r1] Albert, J. H. (1992). Bayesian estimation of normal ogive item response curves using Gibbs sampling. Journal of Educational Statistics, 17(3), 251–269.

[r2] Baker, F. B. , & Kim, S. H. (2004). Item response theory: Parameter estimation techniques. Dekker.

[r3] Béguin, A. A. , & Glas, C. A. (2001). MCMC estimation and some model-fit analysis of multidimensional IRT models. Psychometrika, 66, 541–561.

[r4] Bercu, B. , Godichon, A. , & Portier, B. (2020). An efficient stochastic newton algorithm for parameter estimation in logistic regressions. SIAM Journal on Control and Optimization, 58(1), 348–367.

[r5] Bock, R. D. , & Aitkin, M. (1981). Marginal maximum likelihood estimation of item parameters: Application of an EM algorithm. Psychometrika, 46, 443–459.

[r6] Boyer, C. , & Godichon-Baggioni, A. (2023). On the asymptotic rate of convergence of stochastic newton algorithms and their weighted averaged versions. Computational Optimization and Applications, 84(3), 921–972.

[r7] Cai, L. (2010a). High-dimensional exploratory item factor analysis by a Metropolis–Hastings Robbins–Monro algorithm. Psychometrika, 75(1), 33–57.

[r8] Cai, L. (2010b). Metropolis–Hastings Robbins–Monro algorithm for confirmatory item factor analysis. Journal of Educational and Behavioral Statistics, 35(3), 307–335.

[r9] Cénac, P. , Godichon-Baggioni, A. , & Portier, B. (2025). An efficient averaged stochastic Gauss-Newton algorithm for estimating parameters of nonlinear regressions models. Bernoulli, 31(1), 1–29.

[r10] Chalmers, R. P. (2012). Mirt: A multidimensional item response theory package for the R environment. Journal of Statistical Software, 48, 1–29.

[r11] Cho, A. E. , Wang, C. , Zhang, X. , & Xu, G. (2021). Gaussian variational estimation for multidimensional item response theory. British Journal of Mathematical and Statistical Psychology, 74, 52–85. 33064318 10.1111/bmsp.12219

[r12] Cui, C. , Wang, C. , & Xu, G. (2024). Variational estimation for multidimensional generalized partial credit model. Psychometrika, 89(3), 929–957. 38429494 10.1007/s11336-024-09955-8

[r13] Duchi, J. , Hazan, E. , & Singer, Y. (2011). Adaptive subgradient methods for online learning and stochastic optimization. Journal of Machine Learning Research, 12(61), 2121–2159.

[r14] Godichon-Baggioni, A. (2019). Online estimation of the asymptotic variance for averaged stochastic gradient algorithms. Journal of Statistical Planning and Inference, 203, 1–19.

[r15] Godichon-Baggioni, A. , & Lu, W. (2024). Online stochastic Newton methods for estimating the geometric median and applications. Journal of Multivariate Analysis, 202, 105313.

[r16] Huber, P. , Ronchetti, E. , & Victoria-Feser, M. P. (2004). Estimation of generalized linear latent variable models. Journal of the Royal Statistical Society Series B: Statistical Methodology, 66(4), 893–908.

[r17] Jiang, S. , Xiao, J. , & Wang, C. (2023). On-the-fly parameter estimation based on item response theory in item-based adaptive learning systems. Behavior Research Methods, 55(6), 3260–3280. 36085544 10.3758/s13428-022-01953-x

[r18] Jiang, Z. , & Templin, J. (2019). Gibbs samplers for logistic item response models via the Pólya-Gamma distribution: A computationally efficient data-augmentation strategy. Psychometrika, 84(2), 358–374. 30382548 10.1007/s11336-018-9641-x

[r19] Johnson, M. S. , & Jenkins, F. (2004). A Bayesian hierarchical model for large-scale educational surveys: An application to the National Assessment of Educational Progress. ETS Research Report Series, 2004(2), i-28.

[r20] Martin, M. O. , & Kelly, D. L. (1996). Third international mathematics and science study technical report volume 1: Design and development. Boston College.

[r21] Martin, M. O. , von Davier, M. , & Mullis, I. V. (2020). Methods and procedures: TIMSS 2019 technical report. International Association for the Evaluation of Educational Achievement.

[r22] Meng, X. L. , & Schilling, S. (1996). Fitting full-information item factor models and an empirical investigation of bridge sampling. Journal of the American Statistical Association, 91(435), 1254–1267.

[r23] Mullis, I. V. , & Martin, M. O. (2017). TIMSS 2019 assessment frameworks. ERIC.

[r24] Mullis, I. V. , Martin, M. O. , & Gonzalez, E. J. (2003). PIRLS 2001 international report. International Study Center.

[r25] Muraki, E. , & Engelhard, G., Jr. (1985). Full-information item factor analysis: Applications of EAP scores. Applied Psychological Measurement, 9(4), 417–430.

[r26] OECD. (2021). PISA 2018 technical report. OECD Publishing.

[r27] Rabe-Hesketh, S. , Skrondal, A. , & Pickles, A. (2005). Maximum likelihood estimation of limited and discrete dependent variable models with nested random effects. Journal of Econometrics, 128(2), 301–323.

[r28] Reckase, M. D. (2009). Multidimensional item response theory. Springer.

[r29] Saad, D. (2009). On-line learning in neural networks. Cambridge University Press.

[r30] Schilling, R. , & Bock, D. (2005). High-dimensional maximum marginal likelihood item factor analysis by adaptive quadrature. Psychometrika, 70(3), 533–555.

[r31] Shang, L. , Xu, P. F. , Shan, N. , Tang, M. L. , & Ho, G. T. S . (2023). Accelerating 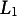 -penalized expectation maximization algorithm for latent variable selection in multidimensional two-parameter logistic models. PLoS One, 18(1), e0279918.36649269 10.1371/journal.pone.0279918PMC9844851

[r32] Song, X. Y. , & Lee, S. Y. (2005). A multivariate probit latent variable model for analyzing dichotomous responses. Statistica Sinica, 15(3), 645–664.

[r33] Thomas, N. (1993). Asymptotic corrections for multivariate posterior moments with factored likelihood functions. Journal of Computational and Graphical Statistics, 2(3), 309–322.

[r34] Urban, C. J. , & Bauer, D. J. (2021). A deep learning algorithm for high-dimensional exploratory item factor analysis. Psychometrika, 86(1), 1–29. 33528784 10.1007/s11336-021-09748-3

[r35] van der Linden, W. J. , & Hambleton, R. K. (1997). Handbook of modern item response theory. Springer-Verlag.

[r36] Wang, C. (2015). On latent trait estimation in multidimensional compensatory item response models. Psychometrika, 80, 428–449. 24604245 10.1007/s11336-013-9399-0

[r37] Weng, C. H. , & Coad, D. S. (2018). Real-time Bayesian parameter estimation for item response models. Bayesian Analysis, 13(1), 115–137.

[r38] Wu, M. , Davis, R. L. , Domingue, B. W. , Piech, C. , & Goodman, N. (2020). *Variational item response theory: Fast, accurate, and expressive*. Preprint. arXiv:2002.00276.

[r39] Zhang, S. , Chen, Y. , & Liu, Y. (2020). An improved stochastic EM algorithm for large-scale full-information item factor analysis. British Journal of Mathematical and Statistical Psychology, 73(1), 44–71. 30511445 10.1111/bmsp.12153

