# Supplementary material for: A Recursive Stochastic Algorithm for Real-Time Online Parameter Estimation in Item Response Theory: Enhancing Computational Efficiency for Dynamic Educational Assessment
Source: Psychometrika. 2025 Dec 23;91(1):3–29. doi: 10.1017/psy.2025.10064 (PMC13121821; doi:10.1017/psy.2025.10064)
Supplement: Xu et al. supplementary material [file S0033312325100641sup001.pdf]

Supplement to "A Recursive Stochastic Algorithm for Real-Time Online Parameter Estimation in Item Response Theory: Enhancing Computational Efficiency for Dynamic Educational Assessment"

A. Additional Figures and Tables from Simulation Study

A.1. Additional Figures and Tables from Simulation Study

Table A1: Average RMSEs for the 2PL model parameter estimates with $J = 40$ across TSNA, TASNA, and EM algorithm implemented in the mirt package.

J	N	K	γ	$\boldsymbol{a}$			$\boldsymbol{b}$			$\boldsymbol{\theta}$		
				TSNA	TASNA	mirt	TSNA	TASNA	mirt	TSNA	TASNA	mirt
40	5,000	10	0.65	0.142	**0.055**	0.051	0.192	**0.060**	0.055	0.328	0.325	0.324
			0.75	0.103	**0.089**		0.121	**0.071**		0.325	0.324	
			0.90	0.112	0.193		0.099	0.112		0.325	0.325	
			1.00	0.184	0.282		0.105	0.107		0.325	0.326	
		20	0.65	0.166	**0.098**		0.174	**0.073**		0.315	0.311	0.308
			0.75	0.100	**0.058**		0.106	**0.056**		0.310	0.310	
			0.90	**0.082**	0.173		**0.081**	0.098		0.312	0.314	
			1.00	0.167	0.271		0.094	0.103		0.314	0.317	
	10,000	10	0.65	0.130	**0.046**	0.036	0.144	**0.041**	0.034	0.326	0.324	0.323
			0.75	**0.099**	**0.073**		0.093	**0.052**		0.324	0.323	
			0.90	**0.096**	0.153		**0.074**	0.089		0.324	0.325	
			1.00	0.153	0.243		0.088	0.105		0.324	0.325	
		20	0.65	0.123	**0.075**		0.125	**0.055**		0.312	0.310	0.307
			0.75	**0.075**	**0.039**		**0.075**	**0.037**		0.309	0.309	
			0.90	**0.057**	0.126		**0.052**	0.071		0.310	0.313	
			1.00	0.130	0.229		0.073	0.097		0.312	0.315	
	20,000	10	0.65	0.109	**0.044**	0.026	0.114	**0.036**	0.025	0.325	0.324	0.324
			0.75	**0.084**	**0.065**		**0.077**	**0.046**		0.324	0.323	
			0.90	**0.082**	0.121		**0.063**	0.078		0.324	0.325	
			1.00	0.127	0.209		0.082	0.109		0.325	0.326	
		20	0.65	0.100	**0.061**		0.099	**0.043**		0.312	0.311	0.308
			0.75	**0.060**	**0.030**		**0.061**	**0.029**		0.310	0.309	
			0.90	**0.038**	0.088		**0.038**	0.055		0.310	0.312	
			1.00	0.099	0.191		0.064	0.097		0.312	0.315	

Table A2: Average RMSEs for the M2PL model parameters with $J = 40$, $Q = 2$ across TSNA, TASNA, and EM algorithms implemented in the mirt package.

K	N	γ	$\boldsymbol{a}_1$			$\boldsymbol{a}_2$			$\boldsymbol{d}$			$\boldsymbol{\theta}$		
			TSNA	TASNA	mirt	TSNA	TASNA	mirt	TSNA	TASNA	mirt	TSNA	TASNA	mirt
10	5,000	0.65	0.156	**0.073**	0.065	0.162	**0.075**	0.063	0.124	**0.061**	0.044	0.524	0.517	0.507
		0.75	0.097	**0.082**		0.101	**0.087**		0.088	**0.071**		0.518	0.519	
		0.90	0.106	0.188		0.123	0.189		0.085	0.123		0.52	0.532	
		1.00	0.197	0.287		0.197	0.275		0.127	0.214		0.532	0.553	
	10,000	0.65	0.154	**0.072**	0.046	0.147	**0.077**	0.045	0.102	**0.051**	0.031	0.521	0.514	0.508
		0.75	**0.089**	**0.062**		**0.086**	**0.063**		**0.073**	**0.067**		0.515	0.515	
		0.90	**0.082**	0.141		0.100	0.146		**0.082**	0.106		0.516	0.523	
		1.00	0.159	0.241		0.158	0.228		0.111	0.175		0.523	0.536	
	20,000	0.65	0.144	**0.081**	0.032	0.139	**0.076**	0.033	0.084	**0.041**	0.024	0.518	0.512	0.508
		0.75	**0.074**	**0.054**		**0.072**	**0.060**		**0.058**	**0.049**		0.513	0.512	
		0.90	**0.066**	0.110		**0.087**	0.124		**0.065**	0.081		0.513	0.517	
		1.00	0.131	0.204		0.139	0.201		0.091	0.135		0.518	0.527	
20	5,000	0.65	0.173	0.099	0.065	0.168	0.091	0.063	0.122	**0.059**	0.044	0.521	0.515	0.505
		0.75	0.100	**0.065**		0.096	**0.068**		0.088	**0.072**		0.516	0.517	
		0.90	0.091	0.178		0.109	0.179		0.086	0.124		0.518	0.530	
		1.00	0.189	0.281		0.188	0.269		0.127	0.214		0.530	0.551	
	10,000	0.65	0.131	**0.088**	0.046	0.126	**0.084**	0.045	0.101	**0.052**	0.031	0.517	0.512	0.506
		0.75	**0.082**	**0.047**		**0.076**	**0.043**		**0.073**	**0.068**		0.513	0.513	
		0.90	**0.063**	0.128		**0.079**	0.131		**0.083**	0.107		0.514	0.521	
		1.00	0.149	0.234		0.146	0.220		0.111	0.175		0.521	0.534	
	20,000	0.65	0.106	**0.066**	0.032	0.107	**0.057**	0.033	0.081	**0.038**	0.024	0.514	0.510	0.506
		0.75	**0.064**	**0.036**		**0.060**	**0.032**		**0.055**	**0.047**		0.510	0.510	
		0.90	**0.042**	0.092		**0.062**	0.106		**0.064**	0.080		0.511	0.515	
		1.00	0.118	0.195		0.125	0.192		0.090	0.134		0.516	0.525	

Table A3: Average RMSEs for the M2PL model parameters with $J = 40$, $Q = 3$ across TSNA, TASNA, and EM algorithm implemented in the mirt package.

K	N	γ	$\boldsymbol{a}_1$ TSNA	$\boldsymbol{a}_1$ TASNA	$\boldsymbol{a}_1$ mirt	$\boldsymbol{a}_2$ TSNA	$\boldsymbol{a}_2$ TASNA	$\boldsymbol{a}_2$ mirt	$\boldsymbol{a}_3$ TSNA	$\boldsymbol{a}_3$ TASNA	$\boldsymbol{a}_3$ mirt	$\boldsymbol{d}$ TSNA	$\boldsymbol{d}$ TASNA	$\boldsymbol{d}$ mirt
10	5,000	0.65	0.172	**0.082**	0.147	0.178	**0.087**	0.171	0.193	**0.109**	0.151	0.137	**0.060**	0.060
		0.75	**0.104**	**0.096**		**0.109**	**0.105**		**0.104**	**0.076**		**0.092**	**0.065**	
		0.90	0.121	0.208		0.140	0.213		**0.098**	0.180		**0.073**	0.138	
		1.00	0.218	0.306		0.223	0.300		0.193	0.275		0.145	0.250	
	10,000	0.65	0.149	**0.075**	0.121	0.161	**0.092**	0.116	0.146	**0.079**	0.149	0.110	**0.043**	0.039
		0.75	**0.091**	**0.069**		**0.091**	**0.067**		**0.086**	**0.057**		**0.072**	**0.044**	
		0.90	**0.097**	0.159		**0.103**	0.158		**0.073**	0.138		**0.057**	0.100	
		1.00	0.181	0.261		0.174	0.249		0.160	0.240		0.113	0.202	
	20,000	0.65	0.126	**0.068**	0.116	0.136	**0.075**	0.126	0.139	**0.075**	0.143	0.088	**0.029**	0.027
		0.75	**0.068**	**0.050**		**0.070**	**0.058**		**0.068**	**0.047**		**0.055**	**0.034**	
		0.90	**0.077**	0.128		**0.089**	0.136		**0.057**	0.107		**0.051**	0.075	
		1.00	0.152	0.227		0.155	0.225		0.131	0.206		0.093	0.159	
20	5,000	0.65	0.181	**0.091**	0.147	0.176	**0.091**	0.171	0.205	**0.127**	0.151	0.134	**0.054**	0.060
		0.75	**0.102**	**0.083**		**0.104**	**0.092**		**0.113**	**0.074**		**0.090**	**0.064**	
		0.90	**0.111**	0.201		**0.130**	0.206		**0.091**	0.174		**0.073**	0.139	
		1.00	0.212	0.302		0.217	0.296		0.188	0.271		0.145	0.251	
	10,000	0.65	0.131	**0.075**	0.121	0.131	**0.079**	0.116	0.137	**0.100**	0.149	0.109	**0.036**	0.039
		0.75	**0.081**	**0.054**		**0.081**	**0.052**		**0.086**	**0.056**		**0.071**	**0.042**	
		0.90	**0.085**	0.150		**0.090**	0.149		**0.063**	0.130		**0.057**	0.100	
		1.00	0.173	0.256		0.167	0.244		0.154	0.236		0.113	0.202	
	20,000	0.65	0.105	**0.059**	0.116	0.106	**0.055**	0.126	0.117	**0.083**	0.143	0.086	**0.025**	0.027
		0.75	**0.062**	**0.036**		**0.061**	**0.041**		**0.071**	**0.047**		**0.054**	**0.033**	
		0.90	**0.063**	0.118		**0.074**	0.125		**0.046**	0.097		**0.052**	0.076	
		1.00	0.144	0.221		0.147	0.219		0.124	0.200		0.094	0.160	

Table A4: Average RMSEs for the M2PL model parameters with $K = 10$, $Q = 4$ across TSNA, TASNA, and EM algorithm implemented in the mirt package.

J	N	γ	$\boldsymbol{a}_1$			$\boldsymbol{a}_2$			$\boldsymbol{a}_3$			$\boldsymbol{a}_4$			$\boldsymbol{d}$		
			TSNA	TASNA	mirt	TSNA	TASNA	mirt	TSNA	TASNA	mirt	TSNA	TASNA	mirt	TSNA	TASNA	mirt
20	5,000	0.65	0.161	**0.093**	0.185	0.183	**0.139**	0.193	0.147	**0.091**	0.238	0.155	**0.102**	0.259	0.129	**0.055**	0.054
		0.75	**0.103**	**0.101**		**0.132**	**0.128**		**0.112**	0.140		**0.100**	**0.104**		**0.085**	**0.076**	
		0.90	0.138	0.232		0.165	0.233		0.190	0.264		0.139	0.219		**0.091**	0.168	
		1.00	0.255	0.331		0.266	0.316		0.274	0.335		0.232	0.300		0.170	0.274	
	10,000	0.65	0.127	**0.081**	0.155	0.131	**0.110**	0.184	0.125	**0.069**	0.197	0.126	**0.077**	0.230	0.110	**0.058**	0.046
		0.75	**0.081**	**0.067**		**0.099**	**0.093**		**0.100**	**0.104**		**0.079**	**0.070**		**0.076**	**0.071**	
		0.90	**0.102**	0.178		**0.114**	0.174		**0.160**	0.225		**0.102**	0.173		**0.079**	0.139	
		1.00	0.221	0.291		0.221	0.273		0.251	0.309		0.198	0.266		0.144	0.229	
	20,000	0.65	0.099	**0.068**	0.156	0.107	**0.087**	0.144	0.098	**0.053**	0.204	0.102	**0.055**	0.207	0.086	**0.035**	0.028
		0.75	**0.066**	**0.052**		**0.086**	**0.078**		**0.080**	**0.087**		**0.065**	**0.052**		**0.051**	**0.043**	
		0.90	**0.075**	0.137		**0.087**	0.138		0.137	0.197		**0.081**	0.142		**0.053**	0.102	
		1.00	0.189	0.255		0.193	0.247		0.231	0.287		0.178	0.242		0.115	0.187	
40	5,000	0.65	0.167	**0.084**	0.259	0.173	**0.092**	0.321	0.172	**0.089**	0.337	0.188	**0.123**	0.322	0.143	**0.064**	0.081
		0.75	**0.108**	**0.104**		**0.117**	**0.124**		**0.105**	**0.093**		**0.111**	**0.082**		**0.097**	**0.073**	
		0.90	0.131	0.218		0.159	0.236		**0.122**	0.202		**0.090**	0.151		**0.088**	0.147	
		1.00	0.227	0.314		0.244	0.317		0.212	0.290		0.167	0.231		0.156	0.255	
	10,000	0.65	0.141	**0.072**	0.245	0.150	**0.082**	0.310	0.145	**0.076**	0.328	0.159	**0.113**	0.330	0.113	**0.044**	0.059
		0.75	**0.088**	**0.070**		**0.094**	**0.085**		**0.087**	**0.068**		**0.094**	**0.069**		**0.072**	**0.047**	
		0.90	**0.101**	0.168		**0.119**	0.180		**0.093**	0.162		**0.060**	0.111		**0.064**	0.109	
		1.00	0.190	0.270		0.195	0.269		0.182	0.260		0.133	0.199		0.125	0.210	
	20,000	0.65	0.127	**0.073**	0.233	0.129	**0.072**	0.302	0.124	**0.061**	0.296	0.121	**0.082**	0.319	0.089	**0.032**	0.038
		0.75	**0.072**	**0.055**		**0.071**	**0.065**		**0.073**	**0.056**		**0.071**	**0.055**		**0.057**	**0.036**	
		0.90	**0.084**	0.137		**0.101**	0.154		**0.078**	0.131		**0.050**	0.087		**0.059**	0.083	
		1.00	0.163	0.238		0.173	0.243		0.153	0.226		0.113	0.172		0.106	0.168	

Table A5: Running time of three methods (in Seconds).

J	N	K	$Q=1$			$Q=2$			$Q=3$			$Q=4$		
			TSNA	TASNA	mirt	TSNA	TASNA	mirt	TSNA	TASNA	mirt	TSNA	TASNA	mirt
20	5,000	10	0.08	0.10	0.35	0.32	0.43	12.32	1.57	2.37	61.12	41.18	60.69	373.88
		20	0.12	0.14		1.04	1.49		26.67	39.43				
	10,000	10	0.20	0.23	0.55	0.64	0.86	22.05	3.65	5.41	100.45	86.83	127.23	722.94
		20	0.26	0.30		2.10	3.04		56.42	82.56				
	20,000	10	0.33	0.39	0.88	1.10	1.50	32.55	6.63	9.85	192.39	163.25	237.95	1091.45
		20	0.47	0.55		4.10	5.96		108.98	158.62				
40	5,000	10	0.16	0.18	0.98	0.66	0.91	47.90	3.12	4.57	299.79	90.27	130.82	1174.31
		20	0.48	0.53		2.03	2.98		60.34	86.61				
	10,000	10	0.34	0.39	1.41	1.27	1.71	81.57	7.03	10.52	623.42	169.42	245.12	2129.13
		20	0.86	0.95		4.12	5.96		117.31	170.01				
	20,000	10	0.78	0.89	2.56	3.34	4.28	146.08	13.53	19.94	1096.46	332.45	480.96	4355.27
		20	1.71	1.87		8.33	11.79		226.09	326.09				

Table A6: Average RMSEs for the $\boldsymbol{\theta}$ parameters with $Q = 3$ across TSNA, TASNA, and EM algorithm implemented in the mirt package.

K	N	γ	$J = 20$			$J = 40$		
			TSNA	TASNA	mirt	TSNA	TASNA	mirt
$K = 10$	$N = 5,000$	0.65	0.686	0.684	0.674	0.609	0.602	0.592
		0.75	0.683	0.687		0.601	0.604	
		0.90	0.690	0.703		0.604	0.617	
		1.00	0.707	0.721		0.618	0.642	
	$N = 10,000$	0.65	0.685	0.682	0.676	0.602	0.596	0.591
		0.75	0.682	0.684		0.596	0.597	
		0.90	0.685	0.695		0.597	0.606	
		1.00	0.700	0.714		0.607	0.625	
	$N = 20,000$	0.65	0.682	0.680	0.676	0.598	0.593	0.591
		0.75	0.680	0.681		0.593	0.594	
		0.90	0.682	0.689		0.594	0.599	
		1.00	0.694	0.706		0.601	0.614	
$K = 20$	$N = 5,000$	0.65	0.686	0.684	0.674	0.608	0.601	0.592
		0.75	0.683	0.687		0.601	0.603	
		0.90	0.689	0.703		0.603	0.617	
		1.00	0.706	0.721		0.617	0.641	
	$N = 10,000$	0.65	0.685	0.682	0.676	0.601	0.595	0.590
		0.75	0.682	0.684		0.595	0.596	
		0.90	0.685	0.695		0.596	0.605	
		1.00	0.700	0.713		0.607	0.625	
	$N = 20,000$	0.65	0.682	0.680	0.676	0.597	0.592	0.591
		0.75	0.680	0.681		0.593	0.593	
		0.90	0.682	0.689		0.593	0.599	
		1.00	0.694	0.706		0.600	0.613	

Table A7: Average RMSEs for the $\boldsymbol{\theta}$ parameters with $K = 10$, $Q = 4$ across TSNA, TASNA, and EM algorithm implemented in the mirt package.

K	N	γ	$J = 20$			$J = 40$		
			TSNA	TASNA	mirt	TSNA	TASNA	mirt
$K = 10$	$N = 5,000$	0.65	0.741	0.739	0.733	0.664	0.657	0.656
		0.75	0.737	0.742		0.656	0.660	
		0.90	0.744	0.755		0.659	0.673	
		1.00	0.758	0.770		0.675	0.697	
	$N = 10,000$	0.65	0.740	0.736	0.734	0.658	0.653	0.656
		0.75	0.736	0.738		0.652	0.654	
		0.90	0.740	0.749		0.653	0.663	
		1.00	0.753	0.764		0.665	0.683	
	$N = 20,000$	0.65	0.735	0.732	0.732	0.651	0.647	0.652
		0.75	0.732	0.733		0.646	0.647	
		0.90	0.735	0.741		0.647	0.654	
		1.00	0.746	0.756		0.656	0.669	

Table A8: Estimated ranges of standard errors for item parameters under a sample size of $N = 20,000$ and latent dimension $Q = 2$.

J	K	γ	TSNA $\boldsymbol{d}$	TSNA $\boldsymbol{a}$	TASNA $\boldsymbol{d}$	TASNA $\boldsymbol{a}$
$J = 20$	$K = 10$	0.65	(0.016,0.039)	(0.017,0.034)	(0.016,0.039)	(0.017,0.034)
		0.75	(0.016,0.038)	(0.017,0.033)	(0.016,0.037)	(0.017,0.032)
		0.90	(0.016,0.036)	(0.017,0.031)	(0.016,0.032)	(0.016,0.028)
		1.00	(0.016,0.032)	(0.016,0.027)	(0.016,0.027)	(0.016,0.026)
	$K = 20$	0.65	(0.016,0.039)	(0.017,0.034)	(0.016,0.039)	(0.017,0.034)
		0.75	(0.016,0.038)	(0.017,0.033)	(0.016,0.037)	(0.017,0.032)
		0.90	(0.016,0.036)	(0.017,0.031)	(0.016,0.032)	(0.016,0.028)
		1.00	(0.016,0.032)	(0.016,0.027)	(0.016,0.027)	(0.016,0.026)
$J = 40$	$K = 10$	0.65	(0.016,0.036)	(0.016,0.035)	(0.016,0.036)	(0.016,0.034)
		0.75	(0.016,0.036)	(0.016,0.033)	(0.016,0.035)	(0.016,0.032)
		0.90	(0.016,0.034)	(0.016,0.032)	(0.016,0.030)	(0.015,0.029)
		1.00	(0.016,0.030)	(0.015,0.028)	(0.016,0.026)	(0.015,0.026)
	$K = 20$	0.65	(0.016,0.036)	(0.017,0.035)	(0.016,0.037)	(0.017,0.035)
		0.75	(0.016,0.036)	(0.016,0.034)	(0.016,0.035)	(0.016,0.033)
		0.90	(0.016,0.034)	(0.016,0.032)	(0.016,0.030)	(0.016,0.029)
		1.00	(0.016,0.030)	(0.015,0.028)	(0.016,0.026)	(0.015,0.026)

Table A9: Estimated ranges of standard errors for item parameters under a sample size of $N = 20,000$ and latent dimension $Q = 3$.

J	K	γ	TSNA $\boldsymbol{d}$	TSNA $\boldsymbol{a}$	TASNA $\boldsymbol{d}$	TASNA $\boldsymbol{a}$
$J = 20$	$K = 10$	0.65	(0.018,0.034)	(0.018,0.034)	(0.018,0.033)	(0.018,0.034)
		0.75	(0.018,0.034)	(0.018,0.034)	(0.018,0.032)	(0.018,0.032)
		0.90	(0.018,0.032)	(0.018,0.031)	(0.018,0.028)	(0.018,0.029)
		1.00	(0.018,0.028)	(0.018,0.028)	(0.018,0.025)	(0.018,0.026)
	$K = 20$	0.65	(0.018,0.034)	(0.018,0.035)	(0.018,0.033)	(0.018,0.034)
		0.75	(0.018,0.034)	(0.018,0.034)	(0.018,0.032)	(0.018,0.033)
		0.90	(0.018,0.032)	(0.018,0.031)	(0.018,0.028)	(0.018,0.029)
		1.00	(0.018,0.028)	(0.018,0.028)	(0.018,0.025)	(0.018,0.026)
$J = 40$	$K = 10$	0.65	(0.018,0.043)	(0.018,0.037)	(0.018,0.042)	(0.018,0.037)
		0.75	(0.018,0.042)	(0.018,0.036)	(0.018,0.040)	(0.018,0.034)
		0.90	(0.018,0.038)	(0.017,0.032)	(0.017,0.033)	(0.017,0.029)
		1.00	(0.018,0.033)	(0.017,0.029)	(0.017,0.028)	(0.017,0.027)
	$K = 20$	0.65	(0.018,0.042)	(0.019,0.037)	(0.018,0.042)	(0.018,0.037)
		0.75	(0.018,0.042)	(0.018,0.036)	(0.018,0.039)	(0.018,0.034)
		0.90	(0.018,0.038)	(0.018,0.032)	(0.017,0.033)	(0.017,0.029)
		1.00	(0.018,0.033)	(0.017,0.029)	(0.017,0.028)	(0.017,0.027)

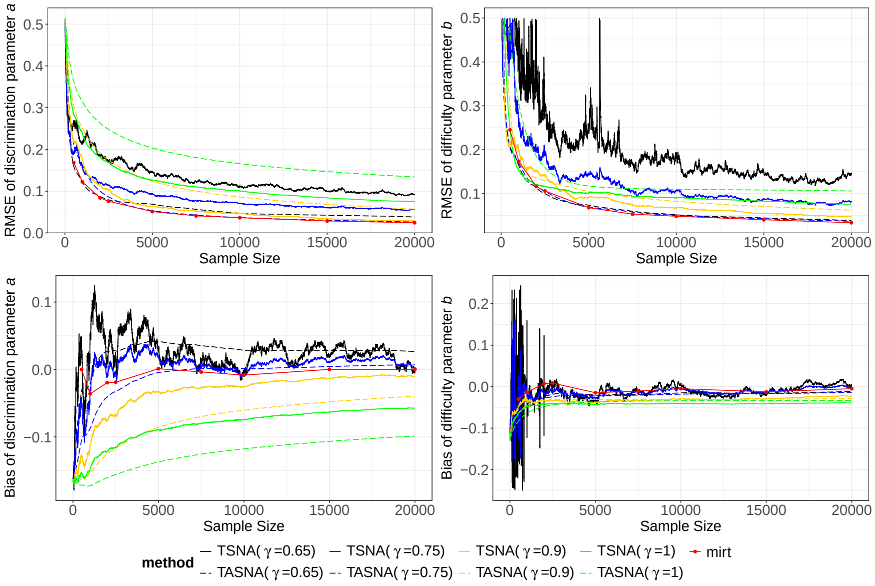

Figure A1: Bias and RMSE of real-time estimates of item parameters in the 2PL model across TSNA, TASNA, and EM algorithm for different step sizes with N=20,000, J=20, and K=20.

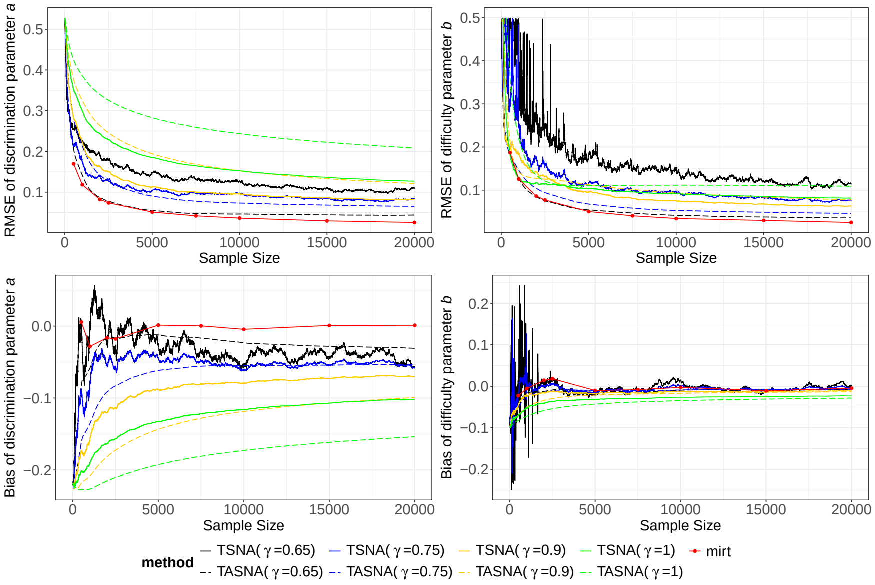

Figure A2: Bias and RMSE of real-time estimates of item parameters in the 2PL model across TSNA, TASNA, and EM algorithm for different step sizes with N=20,000, J=40, and K=10.

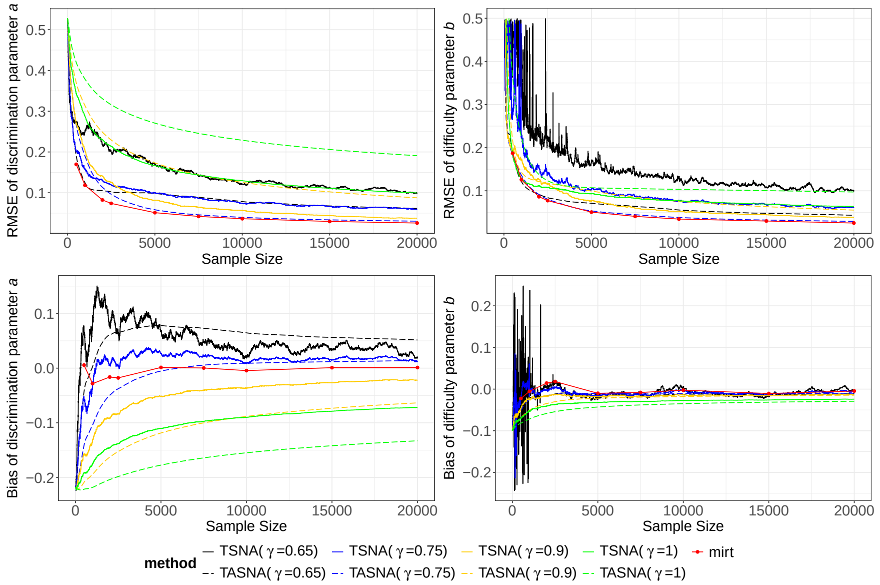

Figure A3: Bias and RMSE of real-time estimates of item parameters in the 2PL model across TSNA, TASNA, and EM algorithm for different step sizes with N=20,000, J=40, and K=20.

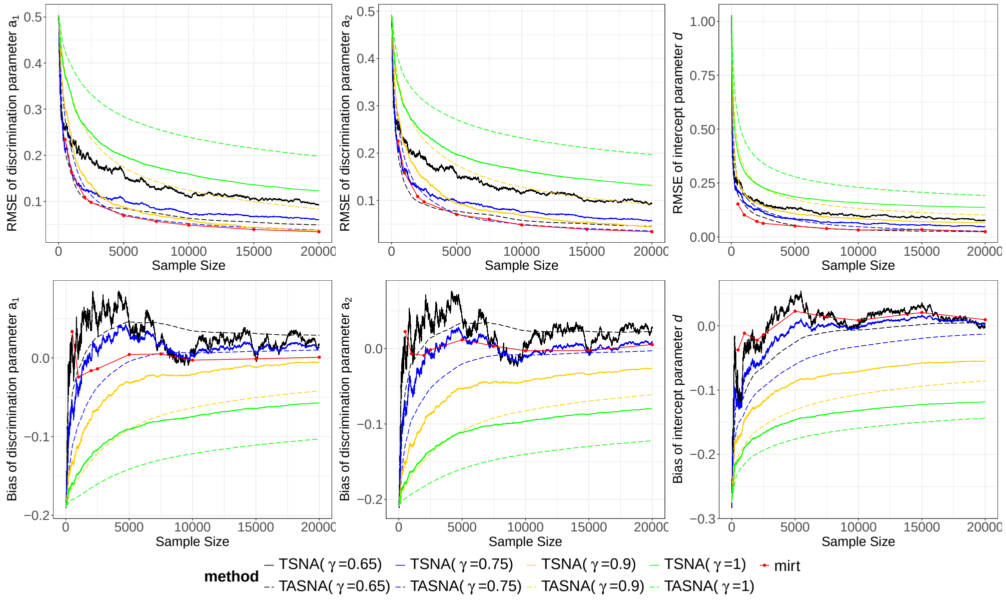

Figure A4: Bias and RMSE of real-time estimates of item parameters in the M2PL model with Q = 2 across TSNA, TASNA, and EM algorithm for different step sizes with N=20,000, J=20, and K=20.

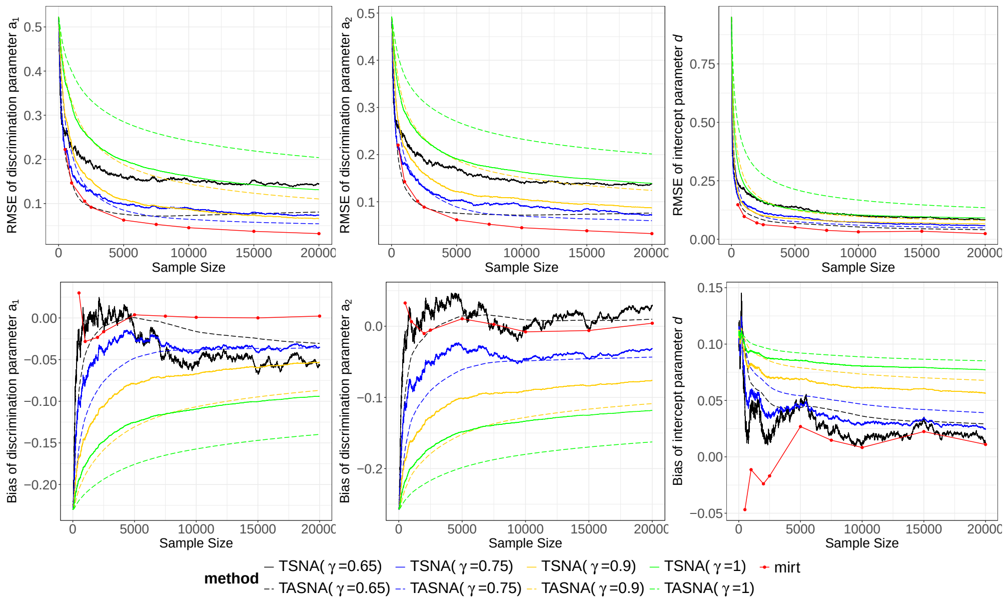

Figure A5: Bias and RMSE of real-time estimates of item parameters in the M2PL model with $Q = 2$ across TSNA, TASNA, and EM algorithm for different step sizes with N=20,000, J=40, and K=10.

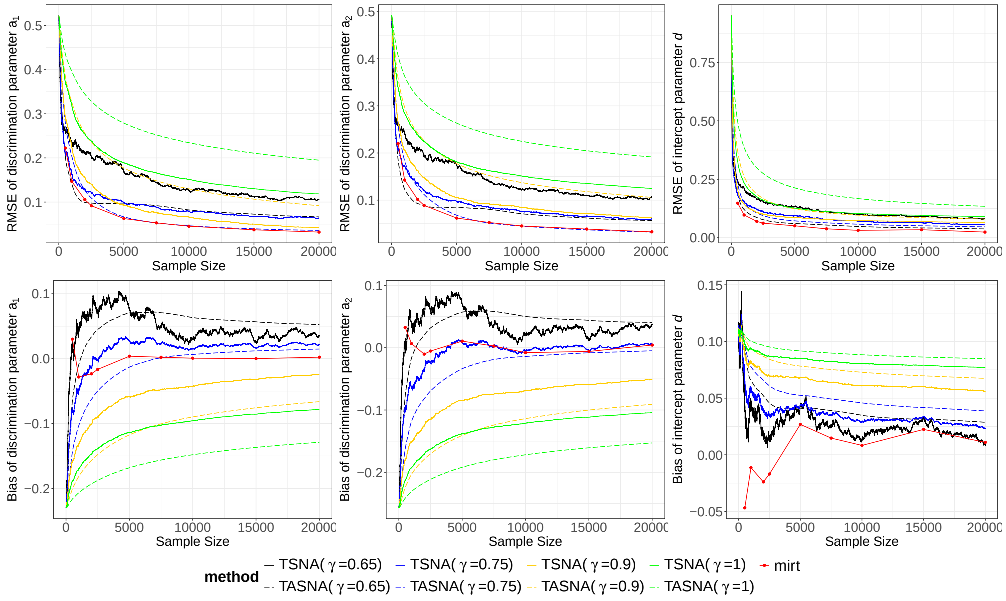

Figure A6: Bias and RMSE of real-time estimates of item parameters in the M2PL model with $Q = 2$ across TSNA, TASNA, and EM algorithm for different step sizes with N=20,000, J=40, and K=20.

A.2. Selection of Tuning Parameters

Regarding the tuning parameters (c_η, c_β, β), we conducted a series of preliminary numerical experiments under a fixed setting: sample size $N = 20,000$, number of items $J = 20$, latent dimensions $Q = 2$, and step-size parameter $\gamma = 0.75$. These experiments focused on different configurations of (c_η, c_β, β).

Specifically, we tested four values of the parameter c_η (1, 50, 100, and 500) to systematically assess its impact on model parameter estimation accuracy, while keeping other tuning parameters consistent with the main simulation study. Table A10 reports the RMSEs of parameter estimates under different c_η values. Although a slight decrease in RMSE is observed as c_η increases, the differences are minimal and can be considered negligible. This suggests that the choice of c_η has little to no effect on the overall algorithm performance.

In addition, we observed an interaction between the tuning parameters c_β and β. To evaluate their combined effect, we ran simulation experiments with four values of c_β (1, 10^{-2}, 10^{-5}, and 10^{-10}), each paired with three different β values. Since β must satisfy $0 < \beta < \gamma - \frac{1}{2}$, we defined $\beta = \gamma - \frac{1}{2} - \beta_0$, and varied β_0 across 0.01, 0.1, and 0.2 to examine the effect of β on estimation accuracy. Table A11 reports RMSEs of model parameter estimates under different combinations of c_β and β_0. The results show that when c_β is set to 1 or 10^{-2}, the large adjustment term leads to noticeably higher RMSEs, and estimation accuracy declines as β_0 increases–$\beta_0 = 0.01$ yields the best results. In contrast, when c_β is sufficiently small (e.g., 10^{-5} or 10^{-10}), estimation accuracy improves significantly and becomes largely insensitive to changes in β_0.

Overall, the tuning parameter c_η has minimal impact on algorithm performance. Its main role is to reduce the influence of poor initial estimates during early iterations, especially when the sample size n is small. To ensure stability when initial values are suboptimal, we recommend setting $c_\eta = 100$. In contrast, c_β and β_0 have a more substantial impact, with c_β being particularly critical. We recommend setting c_β as small as possible–ideally no greater than 10^{-5}–to ensure high estimation accuracy and to minimize the influence of β_0. Although the effect of β_0 is relatively limited, it should still be set to a small value to ensure that β remains sufficiently large, which facilitates the convergence of Hessian matrix estimation. In our

Table A10: Average RMSE of item parameter estimates obtained by the TSNA and TASNA under varying numbers of c_η when $N = 20,000$, $J = 20$, $Q = 2$, and $\gamma = 0.75$.

K	c_η	TSNA			TSNA		
		$\boldsymbol{a}_1$	$\boldsymbol{a}_2$	$\boldsymbol{d}$	$\boldsymbol{a}_1$	$\boldsymbol{a}_2$	$\boldsymbol{d}$
$K = 10$	1	0.062	0.060	0.047	0.043	0.041	0.024
	50	0.060	0.057	0.047	0.038	0.037	0.025
	100	0.060	0.057	0.047	0.037	0.036	0.027
	500	0.058	0.055	0.046	0.038	0.039	0.039
$K = 20$	1	0.062	0.060	0.047	0.045	0.041	0.024
	50	0.061	0.058	0.047	0.040	0.036	0.025
	100	0.061	0.057	0.047	0.038	0.035	0.027
	500	0.059	0.056	0.046	0.037	0.038	0.039

Table A11: Average RMSE of item parameter estimates obtained by the TSNA and TASNA under varying numbers of c_β and β_0 when $N = 20,000$, $J = 20$, $Q = 2$, and $\gamma = 0.75$.

K	c_β	β_0	TSNA			TASNA		
			$\boldsymbol{a}_1$	$\boldsymbol{a}_2$	$\boldsymbol{d}$	$\boldsymbol{a}_1$	$\boldsymbol{a}_2$	$\boldsymbol{d}$
$K = 10$	1	0.01	0.499	0.486	0.807	0.501	0.488	0.879
		0.10	0.501	0.489	0.899	0.502	0.490	0.939
		0.20	0.503	0.490	0.959	0.503	0.491	0.978
	10^{-2}	0.01	0.198	0.209	0.061	0.279	0.283	0.094
		0.10	0.291	0.296	0.085	0.352	0.349	0.127
		0.20	0.381	0.375	0.128	0.415	0.405	0.180
	10^{-5}	0.01	0.059	0.057	0.047	0.037	0.036	0.027
		0.10	0.059	0.056	0.047	0.037	0.036	0.027
		0.20	0.058	0.055	0.046	0.037	0.036	0.027
	10^{-10}	0.01	0.060	0.057	0.047	0.037	0.036	0.027
		0.10	0.060	0.057	0.047	0.037	0.036	0.027
		0.20	0.060	0.057	0.047	0.037	0.036	0.027
$K = 20$	1	0.01	0.502	0.490	0.964	0.503	0.491	0.986
		0.10	0.503	0.491	0.992	0.503	0.491	1.003
		0.20	0.503	0.491	1.009	0.504	0.491	1.014
	10^{-2}	0.01	0.379	0.372	0.135	0.422	0.410	0.210
		0.10	0.434	0.420	0.201	0.456	0.441	0.293
		0.20	0.468	0.452	0.321	0.478	0.463	0.425
	10^{-5}	0.01	0.060	0.056	0.046	0.038	0.036	0.027
		0.10	0.058	0.055	0.046	0.038	0.036	0.027
		0.20	0.056	0.053	0.046	0.037	0.036	0.027
	10^{-10}	0.01	0.061	0.057	0.047	0.038	0.035	0.027
		0.10	0.061	0.057	0.047	0.038	0.035	0.027
		0.20	0.061	0.057	0.047	0.038	0.035	0.027

simulation study, the configuration $c_\beta = 10^{-10}$ and $\beta = \gamma - \frac{1}{2} - 0.01$ satisfies these criteria and yields satisfactory estimation results.

B. Proof of Lemmas

Lemma 1. Assume that the latent abilities follow a multivariate normal distribution $\mathcal{N}(\mathbf{0}, \boldsymbol{I}_Q)$ and the item parameters are bounded. Then, for all $0 < \beta < \gamma - \frac{1}{2}$, we have

$$\lambda_{\max}(\overline{\boldsymbol{S}}_n^{-1}) = O(n^\beta) \quad a.s \quad and \quad \lambda_{\max}(\overline{\boldsymbol{S}}_n) = O(1) \quad a.s, \tag{B.1}$$

where $\overline{\boldsymbol{S}}_{n,j} = \frac{1}{n+1}\boldsymbol{S}_{n,j}$, $\boldsymbol{S}_{n,j} = \boldsymbol{S}_{n-1,j} + \sum_{k=1}^{K^Q} f_k(\widehat{\boldsymbol{\eta}}_{n-1})\alpha_{n,k}\boldsymbol{X}_k\boldsymbol{X}_k^\top$ with $\alpha_{n,k} = \max\left\{\frac{1}{4(\cosh(\boldsymbol{X}_k^\top\widehat{\boldsymbol{\eta}}_{n-1,j}/2))^2}, \frac{c_\beta}{n^\beta}\right\}$. Here, K and Q denote the number of Gauss-Hermite quadrature nodes and the dimension of latent traits, respectively.

Proof. We obtain from $\alpha_{n,k} = \max\left\{\frac{1}{4(\cosh(\boldsymbol{X}_k^\top\widehat{\boldsymbol{\eta}}_{n-1,j}/2))^2}, \frac{c_\beta}{n^\beta}\right\} < \frac{1}{4}$ with $0 < c_\beta < \frac{1}{4}$ that when n is sufficiently large, we have

$$\begin{aligned}\overline{\boldsymbol{S}}_{n,j} &= \frac{1}{n+1}\boldsymbol{S}_{n,j}\\ &= \frac{1}{n+1}\left(\boldsymbol{S}_{0,j} + \sum_{i=1}^{n}\sum_{k=1}^{K^Q} f_k(\widehat{\boldsymbol{\eta}}_{i-1})\alpha_{i,k}\boldsymbol{X}_k\boldsymbol{X}_k^\top\right)\\ &\geq \frac{1}{n+1}\sum_{i=1}^{n}\sum_{k=1}^{K^Q}\frac{c_\beta}{i^\beta} f_k(\widehat{\boldsymbol{\eta}}_{i-1})\boldsymbol{X}_k\boldsymbol{X}_k^\top,\end{aligned} \tag{B.2}$$

where $\boldsymbol{S}_{0,j}$ is the initial matrix and $\boldsymbol{X}_k = (1, x_{k_1}, \ldots, x_{k_Q})$ represents the vector of quadrature nodes. Hence, we have

$$\lambda_{\min}(\overline{\boldsymbol{S}}_{n,j}) \geq \frac{1}{n+1}\sum_i \frac{c_\beta}{i^\beta}\lambda_{\min}\left(\sum_k f_k(\widehat{\boldsymbol{\eta}}_{i-1})\boldsymbol{X}_k\boldsymbol{X}_k^\top\right).$$

According to Assumption (A3), the matrix $\sum_k f_k(\widehat{\boldsymbol{\eta}}_{i-1})\boldsymbol{X}_k\boldsymbol{X}_k^\top$ is positive definite, and its

smallest eigenvalue is uniformly bounded below by a positive constant $c_f > 0$, i.e.,

$$\lambda_{\min}(\sum_k f_k(\widehat{\boldsymbol{\eta}}_{i-1})\boldsymbol{X}_k\boldsymbol{X}_k^\top) \geq c_f > 0.$$

Furthermore,

$$\begin{aligned}\sum_{i=1}^{n}\frac{1}{i^\beta} &= 1 + \frac{1}{2^\beta} + \cdots + \frac{1}{n^\beta} = \frac{1}{n^\beta}\left(n^\beta + (\frac{n}{2})^\beta + \cdots + 1\right) \\ &= n^{1-\beta}\cdot\frac{1}{n}\left(\frac{1}{(\frac{1}{n})^\beta} + \frac{1}{(\frac{2}{n})^\beta} + \cdots + \frac{1}{(\frac{n}{n})^\beta}\right) \\ &= n^{1-\beta}\int_0^1 \frac{1}{x^\beta}dx = \frac{n^{1-\beta}}{1-\beta}. \end{aligned} \tag{B.3}$$

We can deduce that $\lambda_{\min}(\overline{\boldsymbol{S}}_{n,j}) = O(n^{-\beta})$. Hence, we have

$$\lambda_{\max}(\overline{\boldsymbol{S}}_{n,j}^{-1}) = \frac{1}{\lambda_{\min}(\overline{\boldsymbol{S}}_{n,j})} = O(n^\beta) \qquad a.s. \tag{B.4}$$

Next, consider $\lambda_{\max}(\overline{\boldsymbol{S}}_{n,j})$, the maximum eigenvalue of $\overline{\boldsymbol{S}}_{n,j}$. For sufficiently large n, we have

$$\overline{\boldsymbol{S}}_{n,j} = \frac{1}{n+1}(\boldsymbol{S}_{0,j} + \sum_i\sum_k f_k(\widehat{\boldsymbol{\eta}}_{i-1})\alpha_{i,k}\boldsymbol{X}_k\boldsymbol{X}_k^\top) < \frac{1}{n+1}(\boldsymbol{S}_{0,j} + \frac{1}{4}\sum_i\sum_k f_k(\widehat{\boldsymbol{\eta}}_{i-1})\boldsymbol{X}_k\boldsymbol{X}_k^\top). \tag{B.5}$$

Since

$$f_k(\widehat{\boldsymbol{\eta}}_{n-1}) = \frac{\prod_{j=1}^{J}\pi(\boldsymbol{X}_k^\top\widehat{\boldsymbol{\eta}}_{n-1,j})^{y_{nj}}\left(1-\pi(\boldsymbol{X}_k^\top\widehat{\boldsymbol{\eta}}_{n-1,j})\right)^{1-y_{nj}}\boldsymbol{w}(\boldsymbol{x}_k)}{\sum_{k=1}^{KQ}\prod_{j=1}^{J}\pi(\boldsymbol{X}_k^\top\widehat{\boldsymbol{\eta}}_{n-1,j})^{y_{nj}}\left(1-\pi(\boldsymbol{X}_k^\top\widehat{\boldsymbol{\eta}}_{n-1,j})\right)^{1-y_{nj}}\boldsymbol{w}(\boldsymbol{x}_k)}, \tag{B.6}$$

and since $f_k(\widehat{\boldsymbol{\eta}}_{n-1})$ satisfies $\sum_{k=1}^{KQ} f_k(\widehat{\boldsymbol{\eta}}_{n-1}) = 1$, and the quadrature nodes satisfy $\|\boldsymbol{X}_k\| = O(1)$ under Assumptions (A1) and (A2), it follows that $\|\sum_k f_k(\widehat{\boldsymbol{\eta}}_{i-1})\boldsymbol{X}_k\boldsymbol{X}_k^\top\| = O(1)$. Hence, we deduce that

$$\lambda_{\max}(\overline{\boldsymbol{S}}_{n,j}) = O(1) \qquad a.s. \tag{B.7}$$

Lemma 2. For all $\boldsymbol{h}, \boldsymbol{\ell}, \boldsymbol{\ell}_1 \in \mathbb{R}^{Q+1}$, the following inequality holds

$$|\alpha(\boldsymbol{h}^\top \boldsymbol{\ell}) - \alpha(\boldsymbol{h}^\top \boldsymbol{\ell}_1)| \leq \frac{1}{12\sqrt{3}} \|\boldsymbol{h}\| \|\boldsymbol{\ell} - \boldsymbol{\ell}_1\| \qquad \text{a.s,} \tag{B.8}$$

where $\alpha(\boldsymbol{h}^\top \boldsymbol{\ell}) = \pi(\boldsymbol{h}^\top \boldsymbol{\ell})(1 - \pi(\boldsymbol{h}^\top \boldsymbol{\ell}))$, and $\pi(x) = \frac{\exp(x)}{1+\exp(x)}$ represents the logistic sigmoid function.

Proof. The proof adapts the arguments presented in the proof of Lemma 6.2 in Bercu et al. (2020).

Lemma 3. Assume that the latent abilities follow a multivariate normal distribution $\mathcal{N}(\boldsymbol{0}, \boldsymbol{I}_Q)$ and the item parameters are bounded. Then, for test length $J = O(1)$, we have

$$|f_k(\widehat{\boldsymbol{\eta}}) - f_k(\boldsymbol{\eta})| = O(\|\widehat{\boldsymbol{\eta}} - \boldsymbol{\eta}\|) \qquad \text{a.s,} \tag{B.9}$$

where $f_k(\boldsymbol{\eta}) = \frac{L_k(\boldsymbol{\eta}) \boldsymbol{w}(\boldsymbol{x}_k)}{\sum_k L_k(\boldsymbol{\eta}) \boldsymbol{w}(\boldsymbol{x}_k)}$ and $L_k(\boldsymbol{\eta}) = \prod_{j=1}^{J} \pi(\boldsymbol{X}_k^\top \boldsymbol{\eta}_j)^{y_j} \left(1 - \pi(\boldsymbol{X}_k^\top \boldsymbol{\eta}_j)\right)^{1-y_j}$.

Proof. By the Lagrange mean value theorem, there exists at least one point ξ between $\log\{f_k(\widehat{\boldsymbol{\eta}})\}$ and $\log\{f_k(\boldsymbol{\eta})\}$ such that the following inequality holds,

$$\begin{aligned} |f_k(\widehat{\boldsymbol{\eta}}) - f_k(\boldsymbol{\eta})| &= \left| e^{\log\{f_k(\widehat{\boldsymbol{\eta}})\}} - e^{\log\{f_k(\boldsymbol{\eta})\}} \right| \\ &\leq \left| e^{\xi} \right| \cdot \left| \log\{f_k(\widehat{\boldsymbol{\eta}})\} - \log\{f_k(\boldsymbol{\eta})\} \right|, \end{aligned} \tag{B.10}$$

and since $f_k(\boldsymbol{\eta}) \in (0, 1)$, we have $\log\{f_k(\boldsymbol{\eta})\} \in (-\infty, 0)$ for any $\boldsymbol{\eta}$. This implies that $\left| e^{\xi} \right| \leq 1$.

Hence,

$$
\begin{aligned}
&|f_k(\widehat{\boldsymbol{\eta}}) - f_k(\boldsymbol{\eta})| \le |\log\{f_k(\widehat{\boldsymbol{\eta}})\} - \log\{f_k(\boldsymbol{\eta})\}| \\
&= |\log L_k(\widehat{\boldsymbol{\eta}}) + \log \boldsymbol{w}(\boldsymbol{x}_k) - \log \sum_k L_k(\widehat{\boldsymbol{\eta}}) \boldsymbol{w}(\boldsymbol{x}_k) - \log L_k(\boldsymbol{\eta}) - \log \boldsymbol{w}(\boldsymbol{x}_k) + \log \sum_k L_k(\boldsymbol{\eta}) \boldsymbol{w}(\boldsymbol{x}_k)| \\
&\le |\log L_k(\widehat{\boldsymbol{\eta}}) - \log L_k(\boldsymbol{\eta})| + |\log \sum_k L_k(\widehat{\boldsymbol{\eta}}) \boldsymbol{w}(\boldsymbol{x}_k) - \log \sum_k L_k(\boldsymbol{\eta}) \boldsymbol{w}(\boldsymbol{x}_k)| \\
&\triangleq A_1 + A_2. \qquad \text{(B.11)}
\end{aligned}
$$

Consider A_1.

$$
\begin{aligned}
A_1 &= |\log L_k(\widehat{\boldsymbol{\eta}}) - \log L_k(\boldsymbol{\eta})| \\
&\le \sum_{j=1}^{J} \Big| y_j \log \pi(\boldsymbol{X}_k^\top \widehat{\boldsymbol{\eta}}_j) + (1 - y_j) \log \Big(1 - \pi(\boldsymbol{X}_k^\top \widehat{\boldsymbol{\eta}}_j)\Big) \\
&\qquad - y_j \log \pi(\boldsymbol{X}_k^\top \boldsymbol{\eta}_j) - (1 - y_j) \log \Big(1 - \pi(\boldsymbol{X}_k^\top \boldsymbol{\eta}_j)\Big) \Big| \\
&\le \sum_{j=1}^{J} \Big[\Big| y_j \log \frac{\pi(\boldsymbol{X}_k^\top \widehat{\boldsymbol{\eta}}_j)}{1 - \pi(\boldsymbol{X}_k^\top \widehat{\boldsymbol{\eta}}_j)} - y_j \log \frac{\pi(\boldsymbol{X}_k^\top \boldsymbol{\eta}_j)}{1 - \pi(\boldsymbol{X}_k^\top \boldsymbol{\eta}_j)} \Big| + \Big| \log \frac{1 - \pi(\boldsymbol{X}_k^\top \widehat{\boldsymbol{\eta}}_j)}{1 - \pi(\boldsymbol{X}_k^\top \boldsymbol{\eta}_j)} \Big| \Big] \\
&= \sum_{j=1}^{J} \Big[\Big| y_j \boldsymbol{X}_k^\top \widehat{\boldsymbol{\eta}}_j - y_j \boldsymbol{X}_k^\top \boldsymbol{\eta}_j \Big| + \Big| \log \Big(1 + \frac{\pi(\boldsymbol{X}_k^\top \boldsymbol{\eta}_j) - \pi(\boldsymbol{X}_k^\top \widehat{\boldsymbol{\eta}}_j)}{1 - \pi(\boldsymbol{X}_k^\top \boldsymbol{\eta}_j)} \Big) \Big| \Big]. \qquad \text{(B.12)}
\end{aligned}
$$

Using the inequalities $\log(1 + x) \le \log(1 + |x|)$ and $\log(1 + x) \le x$ for all $x > -1$, and noting that $y_j \in \{0, 1\}$, we obtain

$$
\begin{aligned}
A_1 &\le \sum_{j=1}^{J} \Big[\Big| y_j \boldsymbol{X}_k^\top \widehat{\boldsymbol{\eta}}_j - y_j \boldsymbol{X}_k^\top \boldsymbol{\eta}_j \Big| + \Big| \frac{\pi(\boldsymbol{X}_k^\top \boldsymbol{\eta}_j) - \pi(\boldsymbol{X}_k^\top \widehat{\boldsymbol{\eta}}_j)}{1 - \pi(\boldsymbol{X}_k^\top \boldsymbol{\eta}_j)} \Big| \Big] \\
&\le \sum_{j=1}^{J} \Big[\|\widehat{\boldsymbol{\eta}}_j - \boldsymbol{\eta}_j\| \|\boldsymbol{X}_k\| + \frac{1}{1 - \pi(\boldsymbol{X}_k^\top \boldsymbol{\eta}_j)} \Big| \pi(\boldsymbol{X}_k^\top \widehat{\boldsymbol{\eta}}_j) - \pi(\boldsymbol{X}_k^\top \boldsymbol{\eta}_j) \Big| \Big]. \qquad \text{(B.13)}
\end{aligned}
$$

By the mean value theorem and noting that $\pi'(x) = \pi(x)(1 - \pi(x)) \le \frac{1}{4}$, we have

$$
\Big| \pi(\boldsymbol{X}_k^\top \widehat{\boldsymbol{\eta}}_j) - \pi(\boldsymbol{X}_k^\top \boldsymbol{\eta}_j) \Big| = \big| \pi'(\epsilon) \boldsymbol{X}_k^\top (\widehat{\boldsymbol{\eta}}_j - \boldsymbol{\eta}_j) \big| \le \frac{1}{4} \|\boldsymbol{X}_k\| \|\widehat{\boldsymbol{\eta}}_j - \boldsymbol{\eta}_j\|, \qquad \text{(B.14)}
$$

where ϵ is an intermediate point between $\boldsymbol{X}_k^\top \widehat{\boldsymbol{\eta}}_j$ and $\boldsymbol{X}_k^\top \boldsymbol{\eta}_j$. Hence, we further obtain

$$A_1 \leq \sum_{j=1}^{J} \Big(1 + \frac{1}{4(1 - \pi(\boldsymbol{X}_k^\top \boldsymbol{\eta}_j))}\Big) \|\widehat{\boldsymbol{\eta}}_j - \boldsymbol{\eta}_j\| \|\boldsymbol{X}_k\|. \tag{B.15}$$

Consider A_2.

$$\begin{aligned}
A_2 &= \left| \log \sum_k L_k(\widehat{\boldsymbol{\eta}}) \boldsymbol{w}(\boldsymbol{x}_k) - \log \sum_k L_k(\boldsymbol{\eta}) \boldsymbol{w}(\boldsymbol{x}_k) \right| \\
&= \left| \log \left(\frac{\sum_k L_k(\widehat{\boldsymbol{\eta}}) \boldsymbol{w}(\boldsymbol{x}_k) - \sum_k L_k(\boldsymbol{\eta}) \boldsymbol{w}(\boldsymbol{x}_k)}{\sum_k L_k(\boldsymbol{\eta}) \boldsymbol{w}(\boldsymbol{x}_k)} + 1 \right) \right| \\
&\leq \left| \frac{\sum_k L_k(\widehat{\boldsymbol{\eta}}) \boldsymbol{w}(\boldsymbol{x}_k) - \sum_k L_k(\boldsymbol{\eta}) \boldsymbol{w}(\boldsymbol{x}_k)}{\sum_k L_k(\boldsymbol{\eta}) \boldsymbol{w}(\boldsymbol{x}_k)} \right| \\
&\leq \frac{1}{\sum_k L_k(\boldsymbol{\eta}) \boldsymbol{w}(\boldsymbol{x}_k)} \sum_k \left| L_k(\widehat{\boldsymbol{\eta}}) - L_k(\boldsymbol{\eta}) \right| \boldsymbol{w}(\boldsymbol{x}_k) \\
&= \frac{1}{\sum_k L_k(\boldsymbol{\eta}) \boldsymbol{w}(\boldsymbol{x}_k)} \sum_k \left| e^{\log L_k(\widehat{\boldsymbol{\eta}})} - e^{\log L_k(\boldsymbol{\eta})} \right| \boldsymbol{w}(\boldsymbol{x}_k).
\end{aligned} \tag{B.16}$$

Using the Lagrange mean value theorem, there exists at least one point ξ_0 between $\log L_k(\widehat{\boldsymbol{\eta}})$ and $\log L_k(\boldsymbol{\eta})$ such that

$$\begin{aligned}
A_2 &\leq \frac{1}{\sum_k L_k(\boldsymbol{\eta}) \boldsymbol{w}(\boldsymbol{x}_k)} \sum_k \left| e^{\xi_0} \right| \cdot \left| \log L_k(\widehat{\boldsymbol{\eta}}) - \log L_k(\boldsymbol{\eta}) \right| \boldsymbol{w}(\boldsymbol{x}_k) \\
&\leq \frac{1}{\sum_k L_k(\boldsymbol{\eta}) \boldsymbol{w}(\boldsymbol{x}_k)} \sum_k \left| \log L_k(\widehat{\boldsymbol{\eta}}) - \log L_k(\boldsymbol{\eta}) \right| \boldsymbol{w}(\boldsymbol{x}_k) \\
&= \frac{1}{\sum_k L_k(\boldsymbol{\eta}) \boldsymbol{w}(\boldsymbol{x}_k)} \sum_k A_1 \boldsymbol{w}(\boldsymbol{x}_k).
\end{aligned} \tag{B.17}$$

Then, we have

$$
\begin{aligned}
|f_k(\widehat{\boldsymbol{\eta}}) - f_k(\boldsymbol{\eta})| &\le A_1 + A_2 \\
&\le \sum_{j=1}^{J} \left(1 + \frac{1}{4(1-\pi(\boldsymbol{X}_k^\top \boldsymbol{\eta}_j))}\right) \|\widehat{\boldsymbol{\eta}}_j - \boldsymbol{\eta}_j\| \|\boldsymbol{X}_k\| \\
&\quad + \frac{1}{\sum_k L_k(\boldsymbol{\eta}) \boldsymbol{w}(\boldsymbol{x}_k)} \sum_k \sum_{j=1}^{J} \left(1 + \frac{1}{4(1-\pi(\boldsymbol{X}_k^\top \boldsymbol{\eta}_j))}\right) \|\widehat{\boldsymbol{\eta}}_j - \boldsymbol{\eta}_j\| \|\boldsymbol{X}_k\| \boldsymbol{w}(\boldsymbol{x}_k).
\end{aligned}
\tag{B.18}
$$

From inequality (B.18), and noting that under Assumptions (A1)–(A2) the item parameters are bounded and the Gauss-Hermite nodes are also bounded, it follows that

$$
\frac{1}{1 + 4(1-\pi(\boldsymbol{X}_k^\top \boldsymbol{\eta}_j))} = O(1),
$$

We can then bound $|f_k(\widehat{\boldsymbol{\eta}}) - f_k(\boldsymbol{\eta})|$ as follows

$$
\begin{aligned}
|f_k(\widehat{\boldsymbol{\eta}}) - f_k(\boldsymbol{\eta})| &\le \sum_{j=1}^{J} C_j \|\widehat{\boldsymbol{\eta}}_j - \boldsymbol{\eta}_j\| \|\boldsymbol{X}_k\| + \frac{1}{\sum_k L_k(\boldsymbol{\eta}) w(\boldsymbol{x}_k)} \sum_k \sum_{j=1}^{J} C_j \|\widehat{\boldsymbol{\eta}}_j - \boldsymbol{\eta}_j\| \|\boldsymbol{X}_k\| \, w(\boldsymbol{x}_k) \\
&\le C \|\widehat{\boldsymbol{\eta}} - \boldsymbol{\eta}\|,
\end{aligned}
$$

where C_j and C are constants independent of n. Consequently, we obtain

$$
|f_k(\widehat{\boldsymbol{\eta}}) - f_k(\boldsymbol{\eta})| = O(\|\widehat{\boldsymbol{\eta}} - \boldsymbol{\eta}\|) \quad a.s. \tag{B.19}
$$

C. Proof of Theorems

C.1. Proof of Theorem 1

Theorem 1. (**Consistency**). Under Assumptions (A1)–(A3), the following result holds:

$$\lim_{n\to\infty} \widetilde{\boldsymbol{\eta}}_n = \boldsymbol{\eta} \qquad a.s, \tag{C.1}$$

$$\lim_{n\to\infty} \widehat{\boldsymbol{\eta}}_n = \boldsymbol{\eta} \qquad a.s, \tag{C.2}$$

and for $j = 1, \cdots, J$

$$\lim_{n\to\infty} \overline{\boldsymbol{S}}_{n,j} = \boldsymbol{S}_j \qquad a.s, \tag{C.3}$$

where $\overline{\boldsymbol{S}}_{n,j} = \frac{1}{n+1}\boldsymbol{S}_{n,j}$, $\boldsymbol{S}_j = \nabla^2 G(\boldsymbol{\eta}_j)$, $\widetilde{\boldsymbol{\eta}}_n = (\widetilde{\boldsymbol{\eta}}_{n,1}^\top, \cdots, \widetilde{\boldsymbol{\eta}}_{n,J}^\top)^\top$, $\widehat{\boldsymbol{\eta}}_n = (\widehat{\boldsymbol{\eta}}_{n,1}^\top, \cdots, \widehat{\boldsymbol{\eta}}_{n,J}^\top)^\top$ and $\boldsymbol{\eta} = (\boldsymbol{\eta}_1^\top, \cdots, \boldsymbol{\eta}_J^\top)^\top$.

Proof. Using a Taylor decomposition of G, there exists $\boldsymbol{\varepsilon}_n$ such that

$$G(\widetilde{\boldsymbol{\eta}}_{n+1}) = G(\widetilde{\boldsymbol{\eta}}_n) + \nabla G(\widetilde{\boldsymbol{\eta}}_n)^\top(\widetilde{\boldsymbol{\eta}}_{n+1} - \widetilde{\boldsymbol{\eta}}_n) + \frac{1}{2}(\widetilde{\boldsymbol{\eta}}_{n+1} - \widetilde{\boldsymbol{\eta}}_n)^\top \nabla^2 G(\boldsymbol{\varepsilon}_n)(\widetilde{\boldsymbol{\eta}}_{n+1} - \widetilde{\boldsymbol{\eta}}_n), \tag{C.4}$$

where $\nabla G(\widetilde{\boldsymbol{\eta}}_n) = (\nabla G(\widetilde{\boldsymbol{\eta}}_{n,1})^\top, \cdots, \nabla G(\widetilde{\boldsymbol{\eta}}_{n,J})^\top)^\top$. Since $\|\boldsymbol{X}_k\| = O(1)$, there exists a constant $C' > 0$ such that $\|\boldsymbol{X}_k\| \leq C'$ for all k. Consequently, it follows that $\|\nabla^2 G(\boldsymbol{\varepsilon}_n)\| \leq C$ for some constant $C > 0$. Therefore, we have

$$\begin{aligned} G(\widetilde{\boldsymbol{\eta}}_{n+1}) &\leq G(\widetilde{\boldsymbol{\eta}}_n) + \nabla G(\widetilde{\boldsymbol{\eta}}_n)^\top(\widetilde{\boldsymbol{\eta}}_{n+1} - \widetilde{\boldsymbol{\eta}}_n) + \frac{C}{2}\|\widetilde{\boldsymbol{\eta}}_{n+1} - \widetilde{\boldsymbol{\eta}}_n\|^2 \\ &= G(\widetilde{\boldsymbol{\eta}}_n) + \sum_{j=1}^{J} \nabla G(\widetilde{\boldsymbol{\eta}}_{n,j})^\top(\widetilde{\boldsymbol{\eta}}_{n+1,j} - \widetilde{\boldsymbol{\eta}}_{n,j}) + \frac{C}{2}\sum_{j=1}^{J}\|\widetilde{\boldsymbol{\eta}}_{n+1,j} - \widetilde{\boldsymbol{\eta}}_{n,j}\|^2, \end{aligned} \tag{C.5}$$

Then, since $\widetilde{\boldsymbol{\eta}}_{n+1,j} = \widetilde{\boldsymbol{\eta}}_{n,j} - \gamma_{n+1}\overline{\boldsymbol{S}}_{n,j}^{-1}\boldsymbol{Z}_{n+1,j}$, we have

$$
\begin{aligned}
G(\widetilde{\boldsymbol{\eta}}_{n+1}) &\le G(\widetilde{\boldsymbol{\eta}}_n) - \gamma_{n+1}\sum_{j=1}^{J}\nabla G(\widetilde{\boldsymbol{\eta}}_{n,j})^\top \overline{\boldsymbol{S}}_{n,j}^{-1}\boldsymbol{Z}_{n+1,j} + \frac{C}{2}\gamma_{n+1}^2\sum_{j=1}^{J}\|\overline{\boldsymbol{S}}_{n,j}^{-1}\boldsymbol{Z}_{n+1,j}\|^2 \\
&\le G(\widetilde{\boldsymbol{\eta}}_n) - \gamma_{n+1}\sum_{j=1}^{J}\nabla G(\widetilde{\boldsymbol{\eta}}_{n,j})^\top \overline{\boldsymbol{S}}_{n,j}^{-1}\boldsymbol{Z}_{n+1,j} + \frac{C}{2}\gamma_{n+1}^2\sum_{j=1}^{J}\|\overline{\boldsymbol{S}}_{n,j}^{-1}\|^2\|\boldsymbol{Z}_{n+1,j}\|^2. \qquad \text{(C.6)}
\end{aligned}
$$

Since

$$
\|\boldsymbol{Z}_{n+1,j}\| = \left\|\sum_k f_k(\widetilde{\boldsymbol{\eta}}_n)\left[\pi(\boldsymbol{X}_k^\top\widetilde{\boldsymbol{\eta}}_{n,j}) - y_{n+1,j}\right]\boldsymbol{X}_k\right\| \le \|\sum_k f_k(\widetilde{\boldsymbol{\eta}}_n)\boldsymbol{X}_k\|,
$$

where

$$
f_k(\widetilde{\boldsymbol{\eta}}_n) = \frac{\prod_{j=1}^{J}\pi(\boldsymbol{X}_k^\top\widetilde{\boldsymbol{\eta}}_{n,j})^{y_{n+1,j}}\left(1-\pi(\boldsymbol{X}_k^\top\widetilde{\boldsymbol{\eta}}_{n,j})\right)^{1-y_{n+1,j}}\boldsymbol{w}(\boldsymbol{x}_k)}{\sum_{k=1}^{KQ}\prod_{j=1}^{J}\pi(\boldsymbol{X}_k^\top\widetilde{\boldsymbol{\eta}}_{n,j})^{y_{n+1,j}}\left(1-\pi(\boldsymbol{X}_k^\top\widetilde{\boldsymbol{\eta}}_{n,j})\right)^{1-y_{n+1,j}}\boldsymbol{w}(\boldsymbol{x}_k)},
$$

which satisfies $\sum_{k=1}^{KQ} f_k(\widetilde{\boldsymbol{\eta}}_n) = 1$. Hence, we obtain the desired bound $\|\boldsymbol{Z}_{n+1,j}\| \le C$, which in turn implies that

$$
G(\widetilde{\boldsymbol{\eta}}_{n+1}) \le G(\widetilde{\boldsymbol{\eta}}_n) - \gamma_{n+1}\sum_{j=1}^{J}\nabla G(\widetilde{\boldsymbol{\eta}}_{n,j})^\top \overline{\boldsymbol{S}}_{n,j}^{-1}\boldsymbol{Z}_{n+1,j} + \frac{CC'}{2}\gamma_{n+1}^2\sum_{j=1}^{J}\|\overline{\boldsymbol{S}}_{n,j}^{-1}\|^2. \qquad \text{(C.7)}
$$

Let $\mathbb{F} = (\mathcal{F}_n)$ be the filtration defined for all $n \ge 1$ by $\mathcal{F}_n = \sigma\left((\boldsymbol{y}_1, \boldsymbol{X}), \cdots, (\boldsymbol{y}_n, \boldsymbol{X})\right)$, where the quadrature node matrix $\boldsymbol{X} = (\boldsymbol{X}_1, \cdots, \boldsymbol{X}_K)$ is shared across observations. Consequently, we have

$$
\begin{aligned}
\mathbb{E}[G(\widetilde{\boldsymbol{\eta}}_{n+1})|\mathcal{F}_n] &\le G(\widetilde{\boldsymbol{\eta}}_n) - \gamma_{n+1}\sum_{j=1}^{J}\nabla G(\widetilde{\boldsymbol{\eta}}_{n,j})^\top \overline{\boldsymbol{S}}_{n,j}^{-1}\nabla G(\widetilde{\boldsymbol{\eta}}_{n,j}) + \frac{CC'}{2}\gamma_{n+1}^2\sum_{j=1}^{J}\|\overline{\boldsymbol{S}}_{n,j}^{-1}\|^2 \\
&= G(\widetilde{\boldsymbol{\eta}}_n) - \gamma_{n+1}\sum_{j=1}^{J}\|\overline{\boldsymbol{S}}_{n,j}^{-\frac{1}{2}}\nabla G(\widetilde{\boldsymbol{\eta}}_{n,j})\|^2 + \frac{CC'}{2}\gamma_{n+1}^2\sum_{j=1}^{J}\|\overline{\boldsymbol{S}}_{n,j}^{-1}\|^2. \qquad \text{(C.8)}
\end{aligned}
$$

Using the Robbins-Siegmund theorem (see Duflo, 2013) with three positive sequences (V_n), (A_n), and (B_n), where $V_n = G(\widetilde{\boldsymbol{\eta}}_n)$, $A_n = \frac{CC'}{2}\gamma_{n+1}^2\sum_{j=1}^{J}\|\overline{\boldsymbol{S}}_{n,j}^{-1}\|^2$, and

$B_n = \gamma_{n+1} \sum_{j=1}^{J} \|\overline{\boldsymbol{S}}_{n,j}^{-\frac{1}{2}} \nabla G(\widetilde{\boldsymbol{\eta}}_{n,j})\|^2$, we have

$$\mathbb{E}\left[V_{n+1}|\mathcal{F}_n\right] \leq V_n + A_n - B_n. \tag{C.9}$$

By Lemma 1 and the condition $\beta < \gamma - \frac{1}{2}$, it follows that

$$\sum_{n=1}^{\infty} \gamma_{n+1}^2 \|\overline{\boldsymbol{S}}_{n,j}^{-1}\|^2 = \sum_{n=1}^{\infty} \gamma_{n+1}^2 \left(\lambda_{\max}(\overline{\boldsymbol{S}}_{n,j}^{-1})\right)^2 < +\infty, \tag{C.10}$$

which further implies that $\sum_{n=1}^{\infty} A_n < +\infty$ for a finite number of items J. Therefore, by applying the Robbins-Siegmund Theorem, $G(\widetilde{\boldsymbol{\eta}}_n)$ converges almost surely to a finite random variable, and

$$\sum_{n=1}^{\infty} \gamma_{n+1} \|\overline{\boldsymbol{S}}_{n,j}^{-\frac{1}{2}} \nabla G(\widetilde{\boldsymbol{\eta}}_{n,j})\|^2 < +\infty. \tag{C.11}$$

Furthermore, since

$$\gamma_{n+1} \|\overline{\boldsymbol{S}}_{n,j}^{-\frac{1}{2}} \nabla G(\widetilde{\boldsymbol{\eta}}_{n,j})\|^2 \geq \gamma_{n+1} \left(\lambda_{\max}(\overline{\boldsymbol{S}}_{n,j})\right)^{-1} \|\nabla G(\widetilde{\boldsymbol{\eta}}_{n,j})\|^2,$$

and by Lemma 1,

$$\sum_{n=1}^{\infty} \gamma_{n+1} \left(\lambda_{\max}(\overline{\boldsymbol{S}}_{n,j})\right)^{-1} = +\infty \quad a.s. \tag{C.12}$$

Consequently, we conclude that $\|\nabla G(\widetilde{\boldsymbol{\eta}}_{n,j})\| \to 0$ almost surely as $n \to \infty$, for all $j = 1, \cdots, J$. Then, by the mean value theorem, we have

$$\nabla G(\widetilde{\boldsymbol{\eta}}_{n,j}) - \nabla G(\boldsymbol{\eta}_j) = \nabla^2 G(\boldsymbol{\xi}_j)(\widetilde{\boldsymbol{\eta}}_{n,j} - \boldsymbol{\eta}_j), \tag{C.13}$$

which impiles

$$\|\widetilde{\boldsymbol{\eta}}_{n,j} - \boldsymbol{\eta}_j\| \leq \| \left(\nabla^2 G(\boldsymbol{\xi}_j)\right)^{-1} \| \|\nabla^2 G(\boldsymbol{\xi}_j)(\widetilde{\boldsymbol{\eta}}_{n,j} - \boldsymbol{\eta}_j)\| = \| \left(\nabla^2 G(\boldsymbol{\xi}_j)\right)^{-1} \| \|\nabla G(\widetilde{\boldsymbol{\eta}}_{n,j}) - \nabla G(\boldsymbol{\eta}_j)\|. \tag{C.14}$$

Here $\boldsymbol{\xi}_j$ is a point between $\widetilde{\boldsymbol{\eta}}_{n,j}$ and $\boldsymbol{\eta}_j$ in the parameter space. By Assumption (A3) and the fact that $\frac{1}{4\cosh^2(\epsilon/2)} > 0$ for any ϵ, the matrix $\nabla^2 G(\boldsymbol{\xi}_j)$ is positive definite, and its smallest eigenvalue is uniformly bounded below by $c > 0$, i.e.,

$$\|\widetilde{\boldsymbol{\eta}}_{n,j} - \boldsymbol{\eta}_j\| \leq c^{-1}(\|\nabla G(\widetilde{\boldsymbol{\eta}}_{n,j})\| + \|\nabla G(\boldsymbol{\eta}_j)\|). \tag{C.15}$$

Taking the limit as $n \to \infty$ and noting that $\|\nabla G(\widetilde{\boldsymbol{\eta}}_{n,j})\| \to 0$ and $\|\nabla G(\boldsymbol{\eta}_j)\| = \varepsilon_K$, where ε_K denotes the numerical integration error introduced by the Gauss-Hermite quadrature. Since the proposed algorithm optimizes an approximated objective function $G(\boldsymbol{\eta})$ rather than the exact marginal log-likelihood, this error is treated as an asymptotically negligible term when the number of quadrature nodes K is sufficiently large, i.e., $\varepsilon_K = o(1)$. A detailed discussion of this approximation and its implications is provided in the Discussion section of the main text. Therefore, we obtain

$$\lim_{n\to\infty} \|\widetilde{\boldsymbol{\eta}}_{n,j} - \boldsymbol{\eta}_j\| = 0 \quad a.s,$$

which implies that $\widetilde{\boldsymbol{\eta}}_{n,j} \to \boldsymbol{\eta}_j$ almost surely as $n \to \infty$ for $j = 1, \cdots, J$.

Furthermore, we have

$$\widehat{\boldsymbol{\eta}}_{n,j} = \frac{n-1}{n}\widehat{\boldsymbol{\eta}}_{n-1,j} + \frac{1}{n}\widetilde{\boldsymbol{\eta}}_{n,j} = \frac{1}{n}\sum_{i=1}^{n}\widetilde{\boldsymbol{\eta}}_{i,j}. \tag{C.16}$$

Since $\widetilde{\boldsymbol{\eta}}_{n,j} \to \boldsymbol{\eta}_j$ almost surely as $n \to \infty$ and the sequence $\{\widetilde{\boldsymbol{\eta}}_{i,j}\}$ is bounded, it follows from the Cesro mean convergence theorem that

$$\lim_{n\to\infty} \widehat{\boldsymbol{\eta}}_{n,j} = \lim_{n\to\infty} \frac{1}{n}\sum_{i=1}^{n}\widetilde{\boldsymbol{\eta}}_{i,j} = \boldsymbol{\eta}_j \qquad a.s. \tag{C.17}$$

Next, we verify the strong consistency of $\overline{\boldsymbol{S}}_{n,j}$

$$\begin{aligned}\overline{\boldsymbol{S}}_{n,j} &= \frac{1}{n+1}\boldsymbol{S}_{n,j}\\ &= \frac{1}{n+1}\left(\boldsymbol{S}_{0,j} + \sum_{i=1}^{n}\sum_{k=1}^{K^Q} f_k(\widehat{\boldsymbol{\eta}}_{i-1})\alpha_{i,k}\boldsymbol{X}_k\boldsymbol{X}_k^\top\right)\\ &= \frac{1}{n+1}\boldsymbol{S}_{0,j} + \frac{1}{n+1}\sum_{i,k}\Big(f_k(\widehat{\boldsymbol{\eta}}_{i-1})\alpha_{i,k} - f_k(\widehat{\boldsymbol{\eta}}_{i-1})\alpha(\boldsymbol{X}_k^\top\widehat{\boldsymbol{\eta}}_{i-1,j})\Big)\boldsymbol{X}_k\boldsymbol{X}_k^\top\\ &\quad + \frac{1}{n+1}\sum_{i,k} f_k(\widehat{\boldsymbol{\eta}}_{i-1})\alpha(\boldsymbol{X}_k^\top\widehat{\boldsymbol{\eta}}_{i-1,j})\boldsymbol{X}_k\boldsymbol{X}_k^\top\\ &\triangleq A_1 + A_2 + A_3,\end{aligned} \tag{C.18}$$

where $\alpha_{i,k} = \max\{\alpha(\boldsymbol{X}_k^\top\widehat{\boldsymbol{\eta}}_{i-1,j}), \frac{c_\beta}{i^\beta}\}$ with $\alpha(\boldsymbol{X}_k^\top\widehat{\boldsymbol{\eta}}_{i-1,j}) = \pi(\boldsymbol{X}_k^\top\widehat{\boldsymbol{\eta}}_{i-1,j})(1-\pi(\boldsymbol{X}_k^\top\widehat{\boldsymbol{\eta}}_{i-1,j}))$. For A_2, observe that $\alpha_{n,k} = \alpha(\boldsymbol{X}_k^\top\widehat{\boldsymbol{\eta}}_{n-1,j})$ whenever $\alpha(\boldsymbol{X}_k^\top\widehat{\boldsymbol{\eta}}_{n-1,j}) \geq \frac{c_\beta}{n^\beta}$. Consequently,

$$\begin{aligned}A_2 &= \frac{1}{n+1}\sum_i\sum_k\Big(f_k(\widehat{\boldsymbol{\eta}}_{i-1})\alpha_{i,k} - f_k(\widehat{\boldsymbol{\eta}}_{i-1})\alpha(\boldsymbol{X}_k^\top\widehat{\boldsymbol{\eta}}_{i-1,j})\Big)\boldsymbol{X}_k\boldsymbol{X}_k^\top\\ &= \frac{1}{n+1}\sum_i\sum_k\Big(f_k(\widehat{\boldsymbol{\eta}}_{i-1})\alpha_{i,k} - f_k(\widehat{\boldsymbol{\eta}}_{i-1})\alpha(\boldsymbol{X}_k^\top\widehat{\boldsymbol{\eta}}_{i-1,j})\Big)\boldsymbol{X}_k\boldsymbol{X}_k^\top I_{\left\{\alpha(\boldsymbol{X}_k^\top\widehat{\boldsymbol{\eta}}_{i-1,j})\leq\frac{c_\beta}{i^\beta}\right\}}.\end{aligned} \tag{C.19}$$

This implies

$$\left\|\frac{1}{n+1}\sum_i\sum_k\Big(f_k(\widehat{\boldsymbol{\eta}}_{i-1})\alpha_{i,k} - f_k(\widehat{\boldsymbol{\eta}}_{i-1})\alpha(\boldsymbol{X}_k^\top\widehat{\boldsymbol{\eta}}_{i-1,j})\Big)\boldsymbol{X}_k\boldsymbol{X}_k^\top\right\| \leq \frac{1}{n+1}\sum_{i=1}^{n}\frac{c_\beta}{i^\beta}\sum_k f_k(\widehat{\boldsymbol{\eta}}_{i-1})\|\boldsymbol{X}_k\|^2. \tag{C.20}$$

Since $\sum_{k=1}^{K^Q} f_k(\widehat{\boldsymbol{\eta}}_{i-1}) = 1$ and $\|\boldsymbol{X}_k\|$ are bounded, and

$$\begin{aligned}\sum_{i=1}^{n}\frac{1}{i^\beta} &= 1 + \frac{1}{2^\beta} + \cdots + \frac{1}{n^\beta} = \frac{1}{n^\beta}\left(n^\beta + (\frac{n}{2})^\beta + \cdots + 1\right)\\ &= n^{1-\beta}\cdot\frac{1}{n}\left(\frac{1}{(\frac{1}{n})^\beta} + \frac{1}{(\frac{2}{n})^\beta} + \cdots + \frac{1}{(\frac{n}{n})^\beta}\right)\\ &= n^{1-\beta}\int_0^1\frac{1}{x^\beta}dx = \frac{n^{1-\beta}}{1-\beta}.\end{aligned} \tag{C.21}$$

Therefore, we have

$$\lim_{n\to\infty} \frac{1}{n+1} \sum_{i=1}^{n} \frac{c_\beta}{i^\beta} \sum_k f_k(\widehat{\boldsymbol{\eta}}_{i-1}) \|\boldsymbol{X}_k\|^2 = 0 \qquad a.s. \tag{C.22}$$

This implies that A_2 converges to 0 almost surely.

Next, we analyze the convergence of A_3, which can be expressed as

$$\begin{aligned} A_3 &= \frac{1}{n+1} \sum_i \sum_k f_k(\widehat{\boldsymbol{\eta}}_{i-1}) \alpha(\boldsymbol{X}_k^\top \widehat{\boldsymbol{\eta}}_{i-1,j}) \boldsymbol{X}_k \boldsymbol{X}_k^\top \\ &= \frac{1}{n+1} \sum_i \sum_k \left(f_k(\widehat{\boldsymbol{\eta}}_{i-1}) - f_k(\boldsymbol{\eta})\right) \left(\alpha(\boldsymbol{X}_k^\top \widehat{\boldsymbol{\eta}}_{i-1,j}) - \alpha(\boldsymbol{X}_k^\top \boldsymbol{\eta}_j)\right) \boldsymbol{X}_k \boldsymbol{X}_k^\top \\ &\quad + \frac{1}{n+1} \sum_i \sum_k \left(f_k(\widehat{\boldsymbol{\eta}}_{i-1}) - f_k(\boldsymbol{\eta})\right) \alpha(\boldsymbol{X}_k^\top \boldsymbol{\eta}_j) \boldsymbol{X}_k \boldsymbol{X}_k^\top \\ &\quad + \frac{1}{n+1} \sum_i \sum_k f_k(\boldsymbol{\eta}) \left(\alpha(\boldsymbol{X}_k^\top \widehat{\boldsymbol{\eta}}_{i-1,j}) - \alpha(\boldsymbol{X}_k^\top \boldsymbol{\eta}_j)\right) \boldsymbol{X}_k \boldsymbol{X}_k^\top \\ &\quad + \frac{1}{n+1} \sum_i \sum_k f_k(\boldsymbol{\eta}) \alpha(\boldsymbol{X}_k^\top \boldsymbol{\eta}_j) \boldsymbol{X}_k \boldsymbol{X}_k^\top \\ &\triangleq A_{31} + A_{32} + A_{33} + A_{34}, \end{aligned} \tag{C.23}$$

where $\alpha(\boldsymbol{X}_k^\top \boldsymbol{\eta}_j) = \pi(\boldsymbol{X}_k^\top \boldsymbol{\eta}_j)(1 - \pi(\boldsymbol{X}_k^\top \boldsymbol{\eta}_j))$. On the one hand, by the standard strong law of large numbers, it follows that

$$\lim_{n\to\infty} A_{34} = \lim_{n\to\infty} \frac{1}{n+1} \sum_i \sum_k f_k(\boldsymbol{\eta}) \alpha(\boldsymbol{X}_k^\top \boldsymbol{\eta}_j) \boldsymbol{X}_k \boldsymbol{X}_k^\top = \boldsymbol{S}_j \qquad a.s. \tag{C.24}$$

On the other hand, using Lemma 2 and Lemma 3, we obtain

$$\begin{aligned} \|A_{31}\| &= \|\frac{1}{n+1} \sum_i \sum_k \left(f_k(\widehat{\boldsymbol{\eta}}_{i-1}) - f_k(\boldsymbol{\eta})\right) \left(\alpha(\boldsymbol{X}_k^\top \widehat{\boldsymbol{\eta}}_{i-1,j}) - \alpha(\boldsymbol{X}_k^\top \boldsymbol{\eta}_j)\right) \boldsymbol{X}_k \boldsymbol{X}_k^\top \| \\ &\le \frac{1}{n+1} \sum_i \sum_k \|f_k(\widehat{\boldsymbol{\eta}}_{i-1}) - f_k(\boldsymbol{\eta})\| \|\alpha(\boldsymbol{X}_k^\top \widehat{\boldsymbol{\eta}}_{i-1,j}) - \alpha(\boldsymbol{X}_k^\top \boldsymbol{\eta}_j)\| \|\boldsymbol{X}_k\|^2 \\ &= O(\frac{1}{n+1} \sum_i \|\widehat{\boldsymbol{\eta}}_{i-1,j} - \boldsymbol{\eta}_j\|^2). \end{aligned} \tag{C.25}$$

Hence, from (C.2), we deduce that

$$\lim_{n\to\infty} A_{31} = 0 \qquad a.s. \tag{C.26}$$

Similarly, we also have

$$\lim_{n\to\infty} A_{32} = 0 \quad a.s, \quad and \quad \lim_{n\to\infty} A_{33} = 0 \quad a.s. \tag{C.27}$$

Consequently, it follows that

$$\lim_{n\to\infty} A_3 = \boldsymbol{S}_j \qquad a.s. \tag{C.28}$$

Finally, we complete the proof that $\lim_{n\to\infty} \overline{\boldsymbol{S}}_{n,j} = \lim_{n\to\infty} A_1 + A_2 + A_3 = \boldsymbol{S}_j$ almost surely.

C.2. Proof of Theorem 2

Theorem 2. (**Convergence Rate**). Under Assumptions (A1)–(A3), for all $\gamma \in (\frac{1}{2}, 1)$ and $0 < \beta < \gamma - \frac{1}{2}$, we have the following results:

$$\|\widetilde{\boldsymbol{\eta}}_n - \boldsymbol{\eta}\|^2 = O(\frac{\log n}{n^\gamma}) \qquad a.s, \tag{C.29}$$

$$\|\widehat{\boldsymbol{\eta}}_n - \boldsymbol{\eta}\|^2 = O(\frac{\log n}{n}) \qquad a.s, \tag{C.30}$$

Furthermore, for $j = 1, \cdots, J$, we have:

$$\|\overline{\boldsymbol{S}}_{n,j} - \boldsymbol{S}_j\|^2 = O\left(\frac{1}{n^{2\beta}}\right) \qquad a.s \qquad \text{and} \qquad \|\overline{\boldsymbol{S}}_{n,j}^{-1} - \boldsymbol{S}_j^{-1}\|^2 = O\left(\frac{1}{n^{2\beta}}\right) \qquad a.s, \tag{C.31}$$

where $\overline{\boldsymbol{S}}_{n,j} = \frac{1}{n+1}\boldsymbol{S}_{n,j}$, $\boldsymbol{S}_j = \nabla^2 G(\boldsymbol{\eta}_j)$, $\widetilde{\boldsymbol{\eta}}_n = (\widetilde{\boldsymbol{\eta}}_{n,1}^\top, \cdots, \widetilde{\boldsymbol{\eta}}_{n,J}^\top)^\top$, $\widehat{\boldsymbol{\eta}}_n = (\widehat{\boldsymbol{\eta}}_{n,1}^\top, \cdots, \widehat{\boldsymbol{\eta}}_{n,J}^\top)^\top$, and $\boldsymbol{\eta} = (\boldsymbol{\eta}_1^\top, \cdots, \boldsymbol{\eta}_J^\top)^\top$.

Proof. (1) Consider $\widetilde{\boldsymbol{\eta}}_{n,j} - \boldsymbol{\eta}_j$ for $j = 1, \cdots, J$.

The proof of $\|\widetilde{\boldsymbol{\eta}}_{n,j} - \boldsymbol{\eta}_j\|$ for $j = 1, \cdots, J$ follows the argument in the proof of Theorem 4.2 in

Boyer and Godichon–Baggioni (2023). In brief, we have

$$
\begin{aligned}
\widetilde{\boldsymbol{\eta}}_{n+1,j} - \boldsymbol{\eta}_j &= \widetilde{\boldsymbol{\eta}}_{n,j} - \boldsymbol{\eta}_j - \gamma_{n+1}\overline{\boldsymbol{S}}_{n,j}^{-1}\boldsymbol{Z}_{n+1,j} \\
&= \widetilde{\boldsymbol{\eta}}_{n,j} - \boldsymbol{\eta}_j - \gamma_{n+1}\overline{\boldsymbol{S}}_{n,j}^{-1}\nabla G(\widetilde{\boldsymbol{\eta}}_{n,j}) + \gamma_{n+1}\overline{\boldsymbol{S}}_{n,j}^{-1}(\nabla G(\widetilde{\boldsymbol{\eta}}_{n,j}) - \boldsymbol{Z}_{n+1,j}) \\
&= \widetilde{\boldsymbol{\eta}}_{n,j} - \boldsymbol{\eta}_j - \gamma_{n+1}\overline{\boldsymbol{S}}_{n,j}^{-1}(\boldsymbol{D}_{n,j} + \boldsymbol{C}_{n+1,j}) + \gamma_{n+1}\overline{\boldsymbol{S}}_{n,j}^{-1}(\nabla G(\widetilde{\boldsymbol{\eta}}_{n,j}) - \boldsymbol{Z}_{n+1,j}) \\
&= \widetilde{\boldsymbol{\eta}}_{n,j} - \boldsymbol{\eta}_j - \gamma_{n+1}\overline{\boldsymbol{S}}_{n,j}^{-1}\boldsymbol{D}_{n,j} + \gamma_{n+1}\overline{\boldsymbol{S}}_{n,j}^{-1}\boldsymbol{\delta}_{n+1,j},
\end{aligned}
\tag{C.32}
$$

where $\nabla G(\widetilde{\boldsymbol{\eta}}_{n,j}) = \mathbb{E}(\boldsymbol{Z}_{n+1,j}) = \boldsymbol{D}_{n,j} + \boldsymbol{C}_{n+1,j}$ and

$$
\boldsymbol{\delta}_{n+1,j} = \nabla^2 G(\widetilde{\boldsymbol{\eta}}_{n,j}) - \boldsymbol{Z}_{n+1,j} - \boldsymbol{C}_{n+1,j} = \boldsymbol{D}_{n,j} - \boldsymbol{Z}_{n+1,j}. \tag{C.33}
$$

Here, $\boldsymbol{D}_{n,j}$ and $\boldsymbol{C}_{n+1,j}$ are defined as follows:

$$
\boldsymbol{D}_{n,j} = \mathbb{E}\left[\sum_k f_k(\widetilde{\boldsymbol{\eta}}_n)\big(\pi(\boldsymbol{X}_k^\top \widetilde{\boldsymbol{\eta}}_{n,j}) - \pi(\boldsymbol{X}_k^\top \boldsymbol{\eta}_j)\big)\boldsymbol{X}_k\right], \tag{C.34}
$$

$$
\boldsymbol{C}_{n+1,j} = \mathbb{E}\left[\sum_k f_k(\widetilde{\boldsymbol{\eta}}_n)\big(\pi(\boldsymbol{X}_k^\top \boldsymbol{\eta}_j) - y_{n+1,j}\big)\boldsymbol{X}_k\right]. \tag{C.35}
$$

By applying a Taylor expansion to the term $\pi(\boldsymbol{X}_k^\top \widetilde{\boldsymbol{\eta}}_{n,j})$ in $\boldsymbol{D}_{n,j}$, we obtain

$$
\begin{aligned}
\boldsymbol{D}_{n,j} &= \boldsymbol{D}_j + \mathbb{E}\left[\sum_k f_k(\widetilde{\boldsymbol{\eta}}_n)\pi(\boldsymbol{X}_k^\top \boldsymbol{\eta}_j)(1 - \pi(\boldsymbol{X}_k^\top \boldsymbol{\eta}_j))\boldsymbol{X}_k\boldsymbol{X}_k^\top\right](\widetilde{\boldsymbol{\eta}}_{n,j} - \boldsymbol{\eta}_j) + \boldsymbol{\varepsilon}_{n,j} \\
&= \boldsymbol{D}_j + \boldsymbol{S}_j(\widetilde{\boldsymbol{\eta}}_{n,j} - \boldsymbol{\eta}_j) \\
&\quad + \mathbb{E}\left[\sum_k (f_k(\widetilde{\boldsymbol{\eta}}_n) - f_k(\boldsymbol{\eta}))\pi(\boldsymbol{X}_k^\top \boldsymbol{\eta}_j)(1 - \pi(\boldsymbol{X}_k^\top \boldsymbol{\eta}_j))\boldsymbol{X}_k\boldsymbol{X}_k^\top\right](\widetilde{\boldsymbol{\eta}}_{n,j} - \boldsymbol{\eta}_j) + \boldsymbol{\varepsilon}_{n,j} \\
&\triangleq \boldsymbol{S}_j(\widetilde{\boldsymbol{\eta}}_{n,j} - \boldsymbol{\eta}_j) + \boldsymbol{\xi}_{n,j}.
\end{aligned}
\tag{C.36}
$$

Here, $\boldsymbol{D}_j = \mathbb{E}\left[\sum_k f_k(\widetilde{\boldsymbol{\eta}}_n)(\pi(\boldsymbol{X}_k^\top \boldsymbol{\eta}_j) - \pi(\boldsymbol{X}_k^\top \boldsymbol{\eta}_j))\boldsymbol{X}_k\right] = 0$,

$$
\boldsymbol{S}_j = \mathbb{E}\left[\sum_k f_k(\boldsymbol{\eta})\pi(\boldsymbol{X}_k^\top \boldsymbol{\eta}_j)(1 - \pi(\boldsymbol{X}_k^\top \boldsymbol{\eta}_j))\boldsymbol{X}_k\boldsymbol{X}_k^\top\right] = \nabla^2 G(\boldsymbol{\eta}_j)
$$

and

$$\boldsymbol{\xi}_{n,j} = \mathbb{E}\left[\sum_k (f_k(\widetilde{\boldsymbol{\eta}}_n) - f_k(\boldsymbol{\eta}))\pi(\boldsymbol{X}_k^\top \boldsymbol{\eta}_j)(1-\pi(\boldsymbol{X}_k^\top \boldsymbol{\eta}_j))\boldsymbol{X}_k\boldsymbol{X}_k^\top\right](\widetilde{\boldsymbol{\eta}}_{n,j} - \boldsymbol{\eta}_j) + \boldsymbol{\varepsilon}_{n,j}, \tag{C.37}$$

where $\boldsymbol{\varepsilon}_{n,j}$ is the remainder term from the Taylor expansion of $\boldsymbol{D}_{n,j}$. Hence, from (C.36), it follows that

$$\begin{aligned}
\widetilde{\boldsymbol{\eta}}_{n+1,j} - \boldsymbol{\eta}_j &= \widetilde{\boldsymbol{\eta}}_{n,j} - \boldsymbol{\eta}_j - \gamma_{n+1}\overline{\boldsymbol{S}}_{n,j}^{-1}\boldsymbol{S}_j(\widetilde{\boldsymbol{\eta}}_{n,j} - \boldsymbol{\eta}_j) - \gamma_{n+1}\overline{\boldsymbol{S}}_{n,j}^{-1}\boldsymbol{\xi}_{n,j} + \gamma_{n+1}\overline{\boldsymbol{S}}_{n,j}^{-1}\boldsymbol{\delta}_{n+1,j} \qquad \text{(C.38)}\\
&= \widetilde{\boldsymbol{\eta}}_{n,j} - \boldsymbol{\eta}_j - \gamma_{n+1}(\overline{\boldsymbol{S}}_{n,j}^{-1} - \boldsymbol{S}_j^{-1})\boldsymbol{S}_j(\widetilde{\boldsymbol{\eta}}_{n,j} - \boldsymbol{\eta}_j) - \gamma_{n+1}\boldsymbol{S}_j^{-1}\boldsymbol{S}_j(\widetilde{\boldsymbol{\eta}}_{n,j} - \boldsymbol{\eta}_j)\\
&\quad - \gamma_{n+1}(\overline{\boldsymbol{S}}_{n,j}^{-1} - \boldsymbol{S}_j^{-1})\boldsymbol{\xi}_{n,j} - \gamma_{n+1}\boldsymbol{S}_j^{-1}\boldsymbol{\xi}_{n,j} + \gamma_{n+1}\overline{\boldsymbol{S}}_{n,j}^{-1}\boldsymbol{\delta}_{n+1,j}\\
&= (1-\gamma_{n+1})(\widetilde{\boldsymbol{\eta}}_{n,j} - \boldsymbol{\eta}_j) - \gamma_{n+1}\boldsymbol{S}_j^{-1}\boldsymbol{\xi}_{n,j} - \gamma_{n+1}(\overline{\boldsymbol{S}}_{n,j}^{-1} - \boldsymbol{S}_j^{-1})\boldsymbol{D}_{n,j} + \gamma_{n+1}\overline{\boldsymbol{S}}_{n,j}^{-1}\boldsymbol{\delta}_{n+1,j},
\end{aligned} \tag{C.39}$$

Furthermore, by induction, it follows that

$$\begin{aligned}
\widetilde{\boldsymbol{\eta}}_{n+1,j} - \boldsymbol{\eta}_j &= \prod_{i=1}^{n+1}(1-\gamma_i)(\widetilde{\boldsymbol{\eta}}_{0,j} - \boldsymbol{\eta}_j) + \sum_{m=0}^{n}\prod_{i=m+2}^{n+1}(1-\gamma_i)\gamma_{m+1}\overline{\boldsymbol{S}}_{m,j}^{-1}\boldsymbol{\delta}_{m+1,j}\\
&\quad - \sum_{m=0}^{n}\prod_{i=m+2}^{n+1}(1-\gamma_i)\gamma_{m+1}\boldsymbol{S}_j^{-1}\boldsymbol{\xi}_{m,j} - \sum_{m=0}^{n}\prod_{i=m+2}^{n+1}(1-\gamma_i)\gamma_{m+1}(\overline{\boldsymbol{S}}_{m,j}^{-1} - \boldsymbol{S}_j^{-1})\boldsymbol{D}_{m,j}\\
&= \beta_{n+1,0}(\widetilde{\boldsymbol{\eta}}_{0,j} - \boldsymbol{\eta}_j) + \sum_{m=0}^{n}\beta_{n+1,m+1}\gamma_{m+1}\overline{\boldsymbol{S}}_{m,j}^{-1}\boldsymbol{\delta}_{m+1,j}\\
&\quad - \sum_{m=0}^{n}\beta_{n+1,m+1}\gamma_{m+1}\boldsymbol{S}_j^{-1}\boldsymbol{\xi}_{m,j} - \sum_{m=0}^{n}\beta_{n+1,m+1}\gamma_{m+1}(\overline{\boldsymbol{S}}_{m,j}^{-1} - \boldsymbol{S}_j^{-1})\boldsymbol{D}_{m,j}\\
&= \beta_{n+1,0}(\widetilde{\boldsymbol{\eta}}_{0,j} - \boldsymbol{\eta}_j) + M_n - \Delta_n.
\end{aligned} \tag{C.40}$$

Here, $\beta_{n+1,m} = \prod_{i=m+1}^{n+1}(1-\gamma_i)$, $\beta_{n+1,n+1} = 1$, $M_n = \sum_{m=0}^{n}\beta_{n+1,m+1}\gamma_{m+1}\overline{\boldsymbol{S}}_{m,j}^{-1}\boldsymbol{\delta}_{m+1,j}$, and

$$\begin{aligned}
\Delta_n &= \sum_{m=0}^{n}\beta_{n+1,m+1}\gamma_{m+1}\boldsymbol{S}_j^{-1}\boldsymbol{\xi}_{m,j} + \sum_{m=0}^{n}\beta_{n+1,m+1}\gamma_{m+1}(\overline{\boldsymbol{S}}_{m,j}^{-1} - \boldsymbol{S}_j^{-1})\boldsymbol{D}_{m,j}\\
&= \sum_{m=0}^{n}\beta_{n+1,m+1}\gamma_{m+1}\overline{\boldsymbol{S}}_{m,j}^{-1}\boldsymbol{\xi}_{m,j} + \sum_{m=0}^{n}\beta_{n+1,m+1}\gamma_{m+1}(\overline{\boldsymbol{S}}_{m,j}^{-1} - \boldsymbol{S}_j^{-1})\boldsymbol{S}_j(\widetilde{\boldsymbol{\eta}}_{m,j} - \boldsymbol{\eta}_j).
\end{aligned} \tag{C.41}$$

By employing the elementary inequality $1-x \leq \exp(-x)$ and using Equation (C.21), it can be shown that

$$|\beta_{n+1,0}| = |\prod_{i=1}^{n+1}(1-\gamma_i)| = O\left(\exp\big(-\frac{(n+1)^{1-\gamma}}{1-\gamma}\big)\right). \tag{C.42}$$

Furthermore, by combining Lemma 3 and Theorem 1, and following the proofs of M_n and Δ_n in Theorems 3.2 and 4.2 of Boyer and Godichon-Baggioni (2023), we obtain

$$\|M_n\|^2 = O(\frac{\ln n}{n^\gamma}) \qquad a.s \qquad \text{and} \qquad \|\Delta_n\|^2 = O(\frac{\ln n}{n^\gamma}) \qquad a.s. \tag{C.43}$$

This ensures that for $j = 1, \cdots, J$,

$$\|\widetilde{\boldsymbol{\eta}}_{n,j} - \boldsymbol{\eta}_j\|^2 = O(\frac{\ln n}{n^\gamma}) \qquad a.s. \tag{C.44}$$

(2) Consider $\|\widehat{\boldsymbol{\eta}}_{n,j} - \boldsymbol{\eta}_j\|$ for $j = 1, \cdots, J$. Combining Equations (C.38), we have

$$\begin{aligned}\widehat{\boldsymbol{\eta}}_{n,j} - \boldsymbol{\eta}_j &= \frac{1}{n}\sum_{i=1}^{n}(\widetilde{\boldsymbol{\eta}}_{i,j} - \boldsymbol{\eta}_j) \\ &= \frac{1}{n}\sum_{i=1}^{n}\boldsymbol{S}_j^{-1}\overline{\boldsymbol{S}}_{i,j}\left[\frac{(\widetilde{\boldsymbol{\eta}}_{i,j} - \boldsymbol{\eta}_j) - (\widetilde{\boldsymbol{\eta}}_{i+1,j} - \boldsymbol{\eta}_j)}{\gamma_{i+1}}\right] - \frac{1}{n}\sum_{i=1}^{n}\boldsymbol{S}_j^{-1}\boldsymbol{\xi}_{i,j} + \frac{1}{n}\sum_{i=1}^{n}\boldsymbol{S}_j^{-1}\boldsymbol{\delta}_{i+1,j}.\end{aligned} \tag{C.45}$$

Considering the convergence rate for the first term $\frac{1}{n}\sum_{i=1}^{n}\boldsymbol{S}_j^{-1}\overline{\boldsymbol{S}}_{i,j}\left[\frac{(\widetilde{\boldsymbol{\eta}}_{i,j} - \boldsymbol{\eta}_j) - (\widetilde{\boldsymbol{\eta}}_{i+1,j} - \boldsymbol{\eta}_j)}{\gamma_{i+1}}\right]$.

Applying Abel's transform, we obtain

$$\begin{aligned}\frac{1}{n}\sum_{i=1}^{n}\overline{\boldsymbol{S}}_{i,j}\left[\frac{(\widetilde{\boldsymbol{\eta}}_{i,j}-\boldsymbol{\eta}_j)-(\widetilde{\boldsymbol{\eta}}_{i+1,j}-\boldsymbol{\eta}_j)}{\gamma_{i+1}}\right] &= \frac{1}{n}\frac{\overline{\boldsymbol{S}}_{1,j}(\widetilde{\boldsymbol{\eta}}_{1,j}-\boldsymbol{\eta}_j)}{\gamma_2}-\frac{1}{n}\frac{\overline{\boldsymbol{S}}_{1,j}(\widetilde{\boldsymbol{\eta}}_{2,j}-\boldsymbol{\eta}_j)}{\gamma_2}+\frac{1}{n}\frac{\overline{\boldsymbol{S}}_{2,j}(\widetilde{\boldsymbol{\eta}}_{2,j}-\boldsymbol{\eta}_j)}{\gamma_3}\\ &\quad+\cdots+\frac{1}{n}\frac{\overline{\boldsymbol{S}}_{n,j}(\widetilde{\boldsymbol{\eta}}_{n,j}-\boldsymbol{\eta}_j)}{\gamma_{n+1}}-\frac{1}{n}\frac{\overline{\boldsymbol{S}}_{n,j}(\widetilde{\boldsymbol{\eta}}_{n+1,j}-\boldsymbol{\eta}_j)}{\gamma_{n+1}}\\ &= \frac{1}{n}\frac{\overline{\boldsymbol{S}}_{1,j}(\widetilde{\boldsymbol{\eta}}_{1,j}-\boldsymbol{\eta}_j)}{\gamma_2}-\frac{1}{n}\frac{\overline{\boldsymbol{S}}_{n,j}(\widetilde{\boldsymbol{\eta}}_{n+1,j}-\boldsymbol{\eta}_j)}{\gamma_{n+1}}\\ &\quad-\frac{1}{n}\sum_{i=2}^{n}(\gamma_i^{-1}\overline{\boldsymbol{S}}_{i-1,j}-\gamma_{i+1}^{-1}\overline{\boldsymbol{S}}_{i,j})(\widetilde{\boldsymbol{\eta}}_{i,j}-\boldsymbol{\eta}_j). \qquad \text{(C.46)}\end{aligned}$$

First, under the assumption of bounded parameters, it is clear that

$$\frac{1}{n}\left\|\frac{\overline{\boldsymbol{S}}_{1,j}(\widetilde{\boldsymbol{\eta}}_{1,j}-\boldsymbol{\eta}_j)}{\gamma_2}\right\| = O\left(\frac{1}{n}\right) \qquad a.s. \qquad \text{(C.47)}$$

Furthermore, using Theorem 1 and since $\overline{\boldsymbol{S}}_{n,j}$ converges almost surely to $\boldsymbol{S}_j$, we have the following result:

$$\frac{1}{n}\frac{1}{\gamma_{n+1}}\|\overline{\boldsymbol{S}}_{n,j}(\widetilde{\boldsymbol{\eta}}_{n+1,j}-\boldsymbol{\eta}_j)\| = O\left(\frac{\sqrt{\ln n}}{n^{1-\frac{\gamma}{2}}}\right) \qquad a.s. \qquad \text{(C.48)}$$

Finally, since

$$\begin{aligned}\overline{\boldsymbol{S}}_{n,j} = \frac{1}{n+1}\boldsymbol{S}_{n,j} &= \frac{1}{n+1}(\boldsymbol{S}_{n-1,j}+\sum_{k=1}^{K^Q}f_k(\widehat{\boldsymbol{\eta}}_{n-1})\alpha_{n,k}\boldsymbol{X}_k\boldsymbol{X}_k^\top)\\ &= \frac{n}{n+1}\overline{\boldsymbol{S}}_{n-1,j}+\frac{1}{n+1}\sum_{k=1}^{K^Q}f_k(\widehat{\boldsymbol{\eta}}_{n-1})\alpha_{n,k}\boldsymbol{X}_k\boldsymbol{X}_k^\top\\ &= \overline{\boldsymbol{S}}_{n-1,j}-\frac{1}{n+1}\overline{\boldsymbol{S}}_{n-1,j}+\frac{1}{n+1}\sum_{k=1}^{K^Q}f_k(\widehat{\boldsymbol{\eta}}_{n-1})\alpha_{n,k}\boldsymbol{X}_k\boldsymbol{X}_k^\top, \qquad \text{(C.49)}\end{aligned}$$

we have

$$\frac{1}{n}\sum_{i=2}^{n}(\gamma_i^{-1}\overline{\boldsymbol{S}}_{i-1,j}-\gamma_{i+1}^{-1}\overline{\boldsymbol{S}}_{i,j})(\widetilde{\boldsymbol{\eta}}_{i,j}-\boldsymbol{\eta}_j) = \frac{1}{n}\sum_{i=2}^{n}(\gamma_i^{-1}-\gamma_{i+1}^{-1})\overline{\boldsymbol{S}}_{i-1,j}(\widetilde{\boldsymbol{\eta}}_{i,j}-\boldsymbol{\eta}_j) + \frac{1}{n}\sum_{i=2}^{n}\gamma_{i+1}^{-1}\frac{1}{i+1}\overline{\boldsymbol{S}}_{i-1,j}(\widetilde{\boldsymbol{\eta}}_{i,j}-\boldsymbol{\eta}_j) - \frac{1}{n}\sum_{i=2}^{n}\gamma_{i+1}^{-1}\frac{1}{i+1}\sum_{k=1}^{K^Q}f_k(\widehat{\boldsymbol{\eta}}_{i-1})\alpha_{i,k}\boldsymbol{X}_k\boldsymbol{X}_k^{\top}(\widetilde{\boldsymbol{\eta}}_{i,j}-\boldsymbol{\eta}_j). \quad \text{(C.50)}$$

Since $\frac{\gamma_n^{-1}}{\gamma_{n+1}^{-1}}=\left(1-\frac{1}{n+1}\right)^{\gamma}=1-\frac{\gamma}{n+1}+o\left(\frac{1}{n+1}\right)$, and since $\overline{\boldsymbol{S}}_{n,j}$ converges almost surely to $\boldsymbol{S}_j$, it follows that

$$\|\frac{1}{n}\sum_{i=2}^{n}(\gamma_i^{-1}-\gamma_{i+1}^{-1})\overline{\boldsymbol{S}}_{i-1,j}(\widetilde{\boldsymbol{\eta}}_{i,j}-\boldsymbol{\eta}_j)\| \leq \frac{1}{n}\sum_{i=2}^{n}\|\gamma_{i+1}^{-1}\left(1-\frac{\gamma_i^{-1}}{\gamma_{i+1}^{-1}}\right)\|\|\overline{\boldsymbol{S}}_{i-1,j}\|\|\widetilde{\boldsymbol{\eta}}_{i,j}-\boldsymbol{\eta}_j\| = O\left(\frac{1}{n}\sum_{i=2}^{n}\frac{1}{i^{1-\gamma}}\frac{\sqrt{\ln i}}{i^{\frac{\gamma}{2}}}\right) = O\left(\frac{\sqrt{\ln n}}{n^{1-\frac{\gamma}{2}}}\right) \quad a.s, \quad \text{(C.51)}$$

and

$$\|\frac{1}{n}\sum_{i=2}^{n}\gamma_{i+1}^{-1}\frac{1}{i+1}\overline{\boldsymbol{S}}_{i-1,j}(\widetilde{\boldsymbol{\eta}}_{i,j}-\boldsymbol{\eta}_j)\| = O\left(\frac{\sqrt{\ln n}}{n^{1-\frac{\gamma}{2}}}\right) \quad a.s. \quad \text{(C.52)}$$

Under Assumptions (A1)–(A2), noting that $\sum_{k=1}^{K^Q}f_k(\widehat{\boldsymbol{\eta}}_{i-1})=1$ and $\alpha_{i,k}\leq\frac{1}{4}$, we obtain the following:

$$\|\frac{1}{n}\sum_{i=2}^{n}\gamma_{i+1}^{-1}\frac{1}{i+1}\sum_{k=1}^{K^Q}f_k(\widehat{\boldsymbol{\eta}}_{i-1})\alpha_{i,k}\boldsymbol{X}_k\boldsymbol{X}_k^{\top}(\widetilde{\boldsymbol{\eta}}_{i,j}-\boldsymbol{\eta}_j)\| = O\left(\frac{\sqrt{\ln n}}{n^{1-\frac{\gamma}{2}}}\right) \quad a.s. \quad \text{(C.53)}$$

Finally, we obtain

$$\|\frac{1}{n}\sum_{i=2}^{n}(\gamma_i^{-1}\overline{\boldsymbol{S}}_{i-1,j}-\gamma_{i+1}^{-1}\overline{\boldsymbol{S}}_{i,j})(\widetilde{\boldsymbol{\eta}}_{i,j}-\boldsymbol{\eta}_j)\| = O\left(\frac{\sqrt{\ln n}}{n^{1-\frac{\gamma}{2}}}\right) \quad a.s. \quad \text{(C.54)}$$

Hence, it follows that

$$\left\| \frac{1}{n} \sum_{i=1}^{n} \boldsymbol{S}_j^{-1} \overline{\boldsymbol{S}}_{i,j} \left[\frac{(\widetilde{\boldsymbol{\eta}}_{i,j} - \boldsymbol{\eta}_j) - (\widetilde{\boldsymbol{\eta}}_{i+1,j} - \boldsymbol{\eta}_j)}{\gamma_{i+1}} \right] \right\| = O\left(\frac{\sqrt{\ln n}}{n^{1-\frac{\gamma}{2}}} \right) \quad a.s. \tag{C.55}$$

Considering the convergence rate for the second term $\frac{1}{n}\sum_{i=1}^{n} \boldsymbol{S}_j^{-1}\boldsymbol{\xi}_{i,j}$.

From Equation (C.37) and Lemma 3, we have

$$\begin{aligned} \|\boldsymbol{\xi}_{n,j}\| &\leq \left\| \mathbb{E}\left[\sum_k \big(f_k(\widetilde{\boldsymbol{\eta}}_n) - f_k(\boldsymbol{\eta})\big) \pi(\boldsymbol{X}_k^\top \boldsymbol{\eta}_j)(1 - \pi(\boldsymbol{X}_k^\top \boldsymbol{\eta}_j)) \boldsymbol{X}_k \boldsymbol{X}_k^\top \right] (\widetilde{\boldsymbol{\eta}}_{n,j} - \boldsymbol{\eta}_j) \right\| + \|\boldsymbol{\varepsilon}_{n,j}\| \\ &= O(\|\widetilde{\boldsymbol{\eta}}_{n,j} - \boldsymbol{\eta}_j\|^2), \end{aligned} \tag{C.56}$$

and with Theorem 1, we obtain

$$\left\| \frac{1}{n} \sum_{i=1}^{n} \boldsymbol{S}_j^{-1} \boldsymbol{\xi}_{i,j} \right\| = O\left(\frac{1}{n} \sum_{i=1}^{n} \frac{\ln i}{i^\gamma} \right) = O\left(\frac{\ln n}{n^\gamma} \right) \qquad a.s. \tag{C.57}$$

Considering the convergence rate for $\frac{1}{n}\sum_{i=1}^{n} \boldsymbol{S}_j^{-1}\boldsymbol{\delta}_{i+1,j}$.

First, in order to apply the Law of Large Numbers and the Central Limit Theorem for martingales, let us calculate

$$\lim_{n\to\infty} \frac{1}{n} \sum_{i=1}^{n} \mathbb{E}\left[\boldsymbol{\delta}_{i+1,j} \boldsymbol{\delta}_{i+1,j}^\top | \mathcal{F}_i \right]. \tag{C.58}$$

From equations (C.33), (C.34) and (C.35), we have

$$\begin{aligned} &\mathbb{E}\left[\boldsymbol{\delta}_{n+1,j} \boldsymbol{\delta}_{n+1,j}^\top | \mathcal{F}_n \right] = \mathbb{E}\left[\boldsymbol{D}_{n,j}\boldsymbol{D}_{n,j}^\top - \boldsymbol{Z}_{n+1,j}\boldsymbol{D}_{n,j}^\top - \boldsymbol{D}_{n,j}\boldsymbol{Z}_{n+1,j}^\top + \boldsymbol{Z}_{n+1,j}\boldsymbol{Z}_{n+1,j}^\top | \mathcal{F}_n \right] \\ &= \boldsymbol{D}_{n,j}\boldsymbol{D}_{n,j}^\top - \nabla G(\widetilde{\boldsymbol{\eta}}_{n,j})\boldsymbol{D}_{n,j}^\top - \boldsymbol{D}_{n,j}\nabla G(\widetilde{\boldsymbol{\eta}}_{n,j})^\top + \mathbb{E}\left[\boldsymbol{Z}_{n+1,j}\boldsymbol{Z}_{n+1,j}^\top | \mathcal{F}_n \right] \\ &= \boldsymbol{D}_{n,j}\boldsymbol{D}_{n,j}^\top - \boldsymbol{D}_{n,j}\boldsymbol{D}_{n,j}^\top - \boldsymbol{D}_{n,j}\boldsymbol{D}_{n,j}^\top + \mathbb{E}\left[\boldsymbol{Z}_{n+1,j}\boldsymbol{Z}_{n+1,j}^\top | \mathcal{F}_n \right] - \boldsymbol{C}_{n+1,j}\boldsymbol{D}_{n,j}^\top - \boldsymbol{D}_{n,j}\boldsymbol{C}_{n+1,j}^\top \\ &= \mathbb{E}\left[\boldsymbol{Z}_{n+1,j}\boldsymbol{Z}_{n+1,j}^\top | \mathcal{F}_n \right] - \boldsymbol{D}_{n,j}\boldsymbol{D}_{n,j}^\top - \boldsymbol{C}_{n+1,j}\boldsymbol{D}_{n,j}^\top - \boldsymbol{D}_{n,j}\boldsymbol{C}_{n+1,j}^\top. \end{aligned} \tag{C.59}$$

By Lemma 3, and since $\widetilde{\boldsymbol{\eta}}_n$ converges almost surely to $\boldsymbol{\eta}$, the Toeplitz lemma implies

$$\lim_{n\to\infty}\frac{1}{n}\sum_{i=1}^{n}\mathbb{E}\left[\boldsymbol{Z}_{i+1,j}\boldsymbol{Z}_{i+1,j}^{\top}|\mathcal{F}_i\right]=\mathbb{E}\left[\nabla_j\ell(\boldsymbol{y},\boldsymbol{\eta})\nabla_j\ell(\boldsymbol{y},\boldsymbol{\eta})^{\top}\right]=\boldsymbol{S}_j \quad a.s, \tag{C.60}$$

$$\lim_{n\to\infty}\frac{1}{n}\sum_{i=1}^{n}\boldsymbol{D}_{i,j}\boldsymbol{D}_{i,j}^{\top}=0 \quad a.s, \tag{C.61}$$

$$\lim_{n\to\infty}\frac{1}{n}\sum_{i=1}^{n}\boldsymbol{D}_{i,j}\boldsymbol{C}_{i+1,j}^{\top}=0 \quad a.s, \tag{C.62}$$

$$\lim_{n\to\infty}\frac{1}{n}\sum_{i=1}^{n}\boldsymbol{C}_{i+1,j}\boldsymbol{D}_{i,j}^{\top}=0 \quad a.s. \tag{C.63}$$

Therefore, it follows that

$$\lim_{n\to\infty}\frac{1}{n}\sum_{i=1}^{n}\boldsymbol{S}_j^{-1}\mathbb{E}\left[\boldsymbol{\delta}_{i+1,j}\boldsymbol{\delta}_{i+1,j}^{\top}|\mathcal{F}_i\right]\boldsymbol{S}_j^{-1}=\boldsymbol{S}_j^{-1} \qquad a.s. \tag{C.64}$$

Applying the law of large numbers for multidimensional martingales given by Theorem 4.3.16 in Duflo (2013), and noting that $\boldsymbol{X}_k$ has finite fourth moments, we obtain the following almost sure convergence rate

$$\frac{1}{n^2}\|\sum_{i=1}^{n}\boldsymbol{S}_j^{-1}\boldsymbol{\delta}_{i+1,j}\|^2=O\left(\frac{\ln n}{n}\right) \qquad a.s. \tag{C.65}$$

Finally, using Equations (C.55), (C.57), and (C.65), we conclude

$$\|\widehat{\boldsymbol{\eta}}_{n,j}-\boldsymbol{\eta}_j\|^2=O\left(\frac{\ln n}{n}\right) \qquad a.s. \tag{C.66}$$

This is precisely the desired result.

(3) Updating the convergence rate of $\overline{\boldsymbol{S}}_{n,j}-\boldsymbol{S}_j$.

Decompose $\overline{\boldsymbol{S}}_{n,j}$ into the following form

$$\begin{aligned}\overline{\boldsymbol{S}}_{n,j} &= \frac{1}{n+1}\sum_{i=1}^{n}\sum_{k=1}^{KQ} f_k(\widehat{\boldsymbol{\eta}}_{i-1})\alpha_{i,k}\boldsymbol{X}_k\boldsymbol{X}_k^\top + \frac{1}{n+1}\boldsymbol{S}_{0,j}\\ &= \frac{1}{n+1}\sum_{i=1}^{n}\left[\sum_{k=1}^{KQ} f_k(\widehat{\boldsymbol{\eta}}_{i-1})\alpha_{i,k}\boldsymbol{X}_k\boldsymbol{X}_k^\top - \mathbb{E}\big(\sum_{k=1}^{KQ} f_k(\widehat{\boldsymbol{\eta}}_{i-1})\alpha_{i,k}\boldsymbol{X}_k\boldsymbol{X}_k^\top|\mathcal{F}_{i-1}\big)\right]\\ &\quad + \frac{1}{n+1}\sum_{i=1}^{n}\mathbb{E}\big(\sum_{k=1}^{KQ} f_k(\widehat{\boldsymbol{\eta}}_{i-1})\alpha_{i,k}\boldsymbol{X}_k\boldsymbol{X}_k^\top|\mathcal{F}_{i-1}\big) + \frac{1}{n+1}\boldsymbol{S}_{0,j}\\ &\triangleq \frac{1}{n+1}R_n + \frac{1}{n+1}P_n + \frac{1}{n+1}\boldsymbol{S}_{0,j}. \end{aligned}\tag{C.67}$$

Following Bercu et al. (2020), it is known that the sequence (R_n) is a locally square-integrable multidimensional martingale. Under Assumptions (A1)(A2), $\boldsymbol{X}_k$ has finite fourth-order moments. Additionally, since $\sum_{k=1}^{KQ} f_k(\widehat{\boldsymbol{\eta}}_{n-1}) = 1$ and $\alpha_{n,k} < \frac{1}{4}$ for all $n \geq 1$, we can apply the strong law of large numbers for multidimensional martingales, as stated in Theorem 4.3.16 of Duflo (2013). This leads to the conclusion that, for any $\epsilon > 0$,

$$\frac{1}{(n+1)^2}\|R_n\|^2 = o\left(\frac{(\ln n)^{1+\epsilon}}{n}\right) \qquad a.s. \tag{C.68}$$

Next, consider the convergence rate for P_n. On the one hand, we have the decomposition

$$\begin{aligned}P_n &= \sum_i \mathbb{E}\left[\sum_k f_k(\widehat{\boldsymbol{\eta}}_{i-1})\alpha_{i,k}\boldsymbol{X}_k\boldsymbol{X}_k^\top|\mathcal{F}_{i-1}\right]\\ &= \sum_i \mathbb{E}\left[\sum_k (f_k(\widehat{\boldsymbol{\eta}}_{i-1})\alpha_{i,k} - f_k(\widehat{\boldsymbol{\eta}}_{i-1})\alpha(\boldsymbol{X}_k^\top\widehat{\boldsymbol{\eta}}_{i-1,j}))\boldsymbol{X}_k\boldsymbol{X}_k^\top|\mathcal{F}_{i-1}\right]\\ &\quad + \sum_i \mathbb{E}\left[\sum_k f_k(\widehat{\boldsymbol{\eta}}_{i-1})\alpha(\boldsymbol{X}_k^\top\widehat{\boldsymbol{\eta}}_{i-1,j})\boldsymbol{X}_k\boldsymbol{X}_k^\top|\mathcal{F}_{i-1}\right].\end{aligned}\tag{C.69}$$

From Equation (C.20), we have

$$\left\|\sum_i \mathbb{E}\left[\sum_k (f_k(\widehat{\boldsymbol{\eta}}_{i-1})\alpha_{i,k} - f_k(\widehat{\boldsymbol{\eta}}_{i-1})\alpha(\boldsymbol{X}_k^\top\widehat{\boldsymbol{\eta}}_{i-1,j}))\boldsymbol{X}_k\boldsymbol{X}_k^\top|\mathcal{F}_{i-1}\right]\right\| = O(n^{1-\beta}) \qquad a.s. \tag{C.70}$$

Furthermore,

$$
\begin{aligned}
&\sum_i \mathbb{E}\big[\sum_k f_k(\widehat{\boldsymbol{\eta}}_{i-1})\alpha(\boldsymbol{X}_k^\top \widehat{\boldsymbol{\eta}}_{i-1,j})\boldsymbol{X}_k\boldsymbol{X}_k^\top|\mathcal{F}_{i-1}\big] \\
&= \sum_i \mathbb{E}\big[\sum_k (f_k(\widehat{\boldsymbol{\eta}}_{i-1}) - f_k(\boldsymbol{\eta}))(\alpha(\boldsymbol{X}_k^\top \widehat{\boldsymbol{\eta}}_{i-1,j}) - \alpha(\boldsymbol{X}_k^\top \boldsymbol{\eta}_j))\boldsymbol{X}_k\boldsymbol{X}_k^\top|\mathcal{F}_{i-1}\big] \\
&\quad + \sum_i \mathbb{E}\big[\sum_k (f_k(\widehat{\boldsymbol{\eta}}_{i-1}) - f_k(\boldsymbol{\eta}))\alpha(\boldsymbol{X}_k^\top \boldsymbol{\eta}_j)\boldsymbol{X}_k\boldsymbol{X}_k^\top|\mathcal{F}_{i-1}\big] \\
&\quad + \sum_i \mathbb{E}\big[\sum_k f_k(\boldsymbol{\eta})(\alpha(\boldsymbol{X}_k^\top \widehat{\boldsymbol{\eta}}_{i-1,j}) - \alpha(\boldsymbol{X}_k^\top \boldsymbol{\eta}_j))\boldsymbol{X}_k\boldsymbol{X}_k^\top|\mathcal{F}_{i-1}\big] \\
&\quad + \sum_i \mathbb{E}\big[\sum_k f_k(\boldsymbol{\eta})\alpha(\boldsymbol{X}_k^\top \boldsymbol{\eta}_j)\boldsymbol{X}_k\boldsymbol{X}_k^\top|\mathcal{F}_{i-1}\big] \\
&= I_1 + I_2 + I_3 + (n+1)\boldsymbol{S}_j - \boldsymbol{S}_j. \qquad \text{(C.71)}
\end{aligned}
$$

By Lemma 2 and Lemma 3, we can verify that

$$
\|I_1 + I_2 + I_3\| = O\left(\sum_{i=1}^n \|\widehat{\boldsymbol{\eta}}_{i,j} - \boldsymbol{\eta}_j\|\right). \qquad \text{(C.72)}
$$

From the result $\|\widehat{\boldsymbol{\eta}}_{n,j} - \boldsymbol{\eta}_j\|^2 = O\left(\frac{\ln n}{n}\right)$, we can deduce the convergence rate of $I_1 + I_2 + I_3$ in Equation (C.72) as follows:

$$
\frac{1}{n+1}\|I_1 + I_2 + I_3\| = O\left(\frac{1}{n+1}\sum_{i=1}^n \|\widehat{\boldsymbol{\eta}}_{i,j} - \boldsymbol{\eta}_j\|\right) = O\left(\frac{\sqrt{\ln n}}{n^{\frac{1}{2}}}\right) \qquad a.s. \qquad \text{(C.73)}
$$

As a result, we have

$$
\begin{aligned}
\left\|\frac{1}{n+1}P_n - \boldsymbol{S}_j\right\| &\leq \frac{1}{n+1}\left\|\sum_i \mathbb{E}\left[\sum_k (f_k(\widehat{\boldsymbol{\eta}}_{i-1})\alpha_{i,k} - f_k(\widehat{\boldsymbol{\eta}}_{i-1})\alpha(\boldsymbol{X}_k^\top \widehat{\boldsymbol{\eta}}_{i-1,j}))\boldsymbol{X}_k\boldsymbol{X}_k^\top|\mathcal{F}_{i-1}\right]\right\| \\
&\quad + \frac{1}{n+1}\|I_1 + I_2 + I_3 - \boldsymbol{S}_j\| \\
&= O\left(\frac{1}{n^\beta} + \frac{\sqrt{\ln n}}{n^{\frac{1}{2}}}\right) \qquad a.s. \qquad \text{(C.74)}
\end{aligned}
$$

Thus, for all $0 < \beta < \gamma - \frac{1}{2}$ and $\gamma \in \left(\frac{1}{2}, 1\right)$, the convergence rate of $\overline{\boldsymbol{S}}_{n,j} - \boldsymbol{S}_j$ is

$$\|\overline{\boldsymbol{S}}_{n,j} - \boldsymbol{S}_j\|^2 = O\left(\frac{1}{n^{2\beta}}\right) \qquad a.s, \tag{C.75}$$

and since $\overline{\boldsymbol{S}}_{n,j}^{-1} - \boldsymbol{S}_j^{-1} = \overline{\boldsymbol{S}}_{n,j}^{-1}(\boldsymbol{S}_j - \overline{\boldsymbol{S}}_{n,j})\boldsymbol{S}_j^{-1}$, we have

$$\|\overline{\boldsymbol{S}}_{n,j}^{-1} - \boldsymbol{S}_j^{-1}\| = O\left(\frac{1}{n^{2\beta}}\right) \qquad a.s. \tag{C.76}$$

C.3. Proof of Theorem 3

Theorem 3. (**Asymptotic Normality**). Under Assumptions (A1)–(A3), for $j = 1, \cdots, J$, the following asymptotic normality holds:

$$\sqrt{n}(\widehat{\boldsymbol{\eta}}_{n,j} - \boldsymbol{\eta}_j) \xrightarrow[n\to\infty]{\mathcal{L}} N(0, \boldsymbol{S}_j^{-1}), \tag{C.77}$$

where $\boldsymbol{S}_j = \nabla^2 G(\boldsymbol{\eta}_j)$.

Proof. From Equation (C.45), we have

$$\begin{aligned}
\sqrt{n}(\widehat{\boldsymbol{\eta}}_{n,j} - \boldsymbol{\eta}_j) &= \frac{1}{\sqrt{n}}\sum_{i=1}^{n}(\widetilde{\boldsymbol{\eta}}_{i,j} - \boldsymbol{\eta}_j) \\
&= \frac{1}{\sqrt{n}}\sum_{i=1}^{n}\boldsymbol{S}_j^{-1}\overline{\boldsymbol{S}}_{i,j}\left[\frac{(\widetilde{\boldsymbol{\eta}}_{i,j} - \boldsymbol{\eta}_j) - (\widetilde{\boldsymbol{\eta}}_{i+1,j} - \boldsymbol{\eta}_j)}{\gamma_{i+1}}\right] - \frac{1}{\sqrt{n}}\sum_{i=1}^{n}\boldsymbol{S}_j^{-1}\boldsymbol{\xi}_{i,j} \\
&\quad + \frac{1}{\sqrt{n}}\sum_{i=1}^{n}\boldsymbol{S}_j^{-1}\boldsymbol{\delta}_{i+1,j} \\
&\triangleq I_{n+1} - R_n + \frac{1}{\sqrt{n}}\overline{M}_{n+1}.
\end{aligned} \tag{C.78}$$

Step 1: Convergence of I_{n+1}. Using Equations (C.47), (C.48), and (C.54), we deduce

$$\begin{aligned}\|I_{n+1}\| &= \left\| \frac{1}{\sqrt{n}} \sum_{i=1}^{n} \boldsymbol{S}_j^{-1} \overline{\boldsymbol{S}}_{i,j} \left[\frac{(\widetilde{\boldsymbol{\eta}}_{i,j} - \boldsymbol{\eta}_j) - (\widetilde{\boldsymbol{\eta}}_{i+1,j} - \boldsymbol{\eta}_j)}{\gamma_{i+1}} \right] \right\| \\ &\leq \left\| \frac{\boldsymbol{S}_j^{-1} \overline{\boldsymbol{S}}_{1,j}}{\sqrt{n}} \frac{\widetilde{\boldsymbol{\eta}}_{1,j} - \boldsymbol{\eta}_j}{\gamma_2} \right\| + \left\| \frac{\boldsymbol{S}_j^{-1} \overline{\boldsymbol{S}}_{n,j}}{\sqrt{n}} \frac{\widetilde{\boldsymbol{\eta}}_{n+1,j} - \boldsymbol{\eta}_j}{\gamma_{n+1}} \right\| \\ &\quad + \left\| \frac{1}{\sqrt{n}} \sum_{i=2}^{n} \boldsymbol{S}_j^{-1} (\gamma_i^{-1} \overline{\boldsymbol{S}}_{i-1,j} - \gamma_{i+1}^{-1} \overline{\boldsymbol{S}}_{i,j}) (\widetilde{\boldsymbol{\eta}}_{i,j} - \boldsymbol{\eta}_j) \right\| \\ &= O\left(\frac{\sqrt{\ln n}}{n^{\frac{1-\gamma}{2}}} \right), \end{aligned} \tag{C.79}$$

which implies:

$$\lim_{n\to\infty} I_{n+1} = 0 \qquad a.s. \tag{C.80}$$

Step 2: Convergence of R_n. From Equations (C.56) and (C.57), we have

$$\|R_n\| = O\left(\frac{1}{\sqrt{n}} \sum_{i=1}^{n} \|\widetilde{\boldsymbol{\eta}}_{i,j} - \boldsymbol{\eta}_j\|^2 \right) = O\left(\frac{1}{\sqrt{n}} \sum_{i=1}^{n} \frac{\ln i}{i^{\gamma}} \right) = O\left(\frac{\ln n}{n^{\gamma - \frac{1}{2}}} \right). \tag{C.81}$$

Hence, we can get

$$\lim_{n\to\infty} R_n = 0 \qquad a.s. \tag{C.82}$$

Step 3: Asymptotic behavior of the martingale term $\overline{M}_n$. Following Bercu et al. (2020), from Equation (C.64), the predictable quadratic variation of $\overline{M}_n$ satisfies

$$\lim_{n\to\infty} \frac{1}{n} \sum_{i=1}^{n} \boldsymbol{S}_j^{-1} \mathbb{E}\left[\boldsymbol{\delta}_{i+1,j} \boldsymbol{\delta}_{i+1,j}^{\top} | \mathcal{F}_i \right] \boldsymbol{S}_j^{-1} = \boldsymbol{S}_j^{-1} \qquad a.s. \tag{C.83}$$

Furthermore, since $\boldsymbol{\delta}_{n+1,j} = \boldsymbol{D}_{n,j} - \boldsymbol{Z}_{n+1,j}$, we have

$$\begin{aligned}
\|\boldsymbol{\delta}_{n+1,j}\| &\leq \|\boldsymbol{Z}_{n+1,j}\| + \|\boldsymbol{D}_{n,j}\| \\
&= \left\| \sum_k f_k(\widetilde{\boldsymbol{\eta}}_n) \left[\pi(\boldsymbol{X}_k^\top \widetilde{\boldsymbol{\eta}}_{n,j}) - y_{n+1,j} \right] \boldsymbol{X}_k \right\| + \left\| \mathbb{E} \left[\sum_k f_k(\widetilde{\boldsymbol{\eta}}_n) \big(\pi(\boldsymbol{X}_k^\top \widetilde{\boldsymbol{\eta}}_{n,j}) - \pi(\boldsymbol{X}_k^\top \boldsymbol{\eta}_j) \big) \boldsymbol{X}_k \right] \right\| \\
&\leq \left\| \sum_k f_k(\widetilde{\boldsymbol{\eta}}_n) \boldsymbol{X}_k \right\| + \left\| \mathbb{E} \left[\sum_k f_k(\widetilde{\boldsymbol{\eta}}_n) \boldsymbol{X}_k \right] \right\|. \qquad (C.84)
\end{aligned}$$

And since $\boldsymbol{X}_k$ has finite moments of order 4, and $\sum_k f_k(\widetilde{\boldsymbol{\eta}}_n) = 1$, we conclude

$$\sup_{n\geq 1} \mathbb{E}\big[\|\boldsymbol{\delta}_{n+1,j}\|^4\big] < \infty. \qquad (C.85)$$

Following the proof of Theorem 4.4 in Bercu et al. (2020), it directly follows that $(\overline{M}_n)$ satisfies Lindeberg's condition. Consequently, applying the central limit theorem for martingales (Corollary 2.1.10, Duflo, 2013), we deduce

$$\frac{1}{\sqrt{n}} \overline{M}_n \xrightarrow[n\to\infty]{\mathcal{L}} N(0, \boldsymbol{S}_j^{-1}). \qquad (C.86)$$

Therefore, we obtain

$$\sqrt{n}(\widehat{\boldsymbol{\eta}}_{n,j} - \boldsymbol{\eta}_j) \xrightarrow[n\to\infty]{\mathcal{L}} N(0, \boldsymbol{S}_j^{-1}), \qquad (C.87)$$

which completes the proof of Theorem 3.

D. Additional Figures and Analysis in Empirical Example

D.1. Additional Figures from Analysis of the Unidimensional 2PL Model

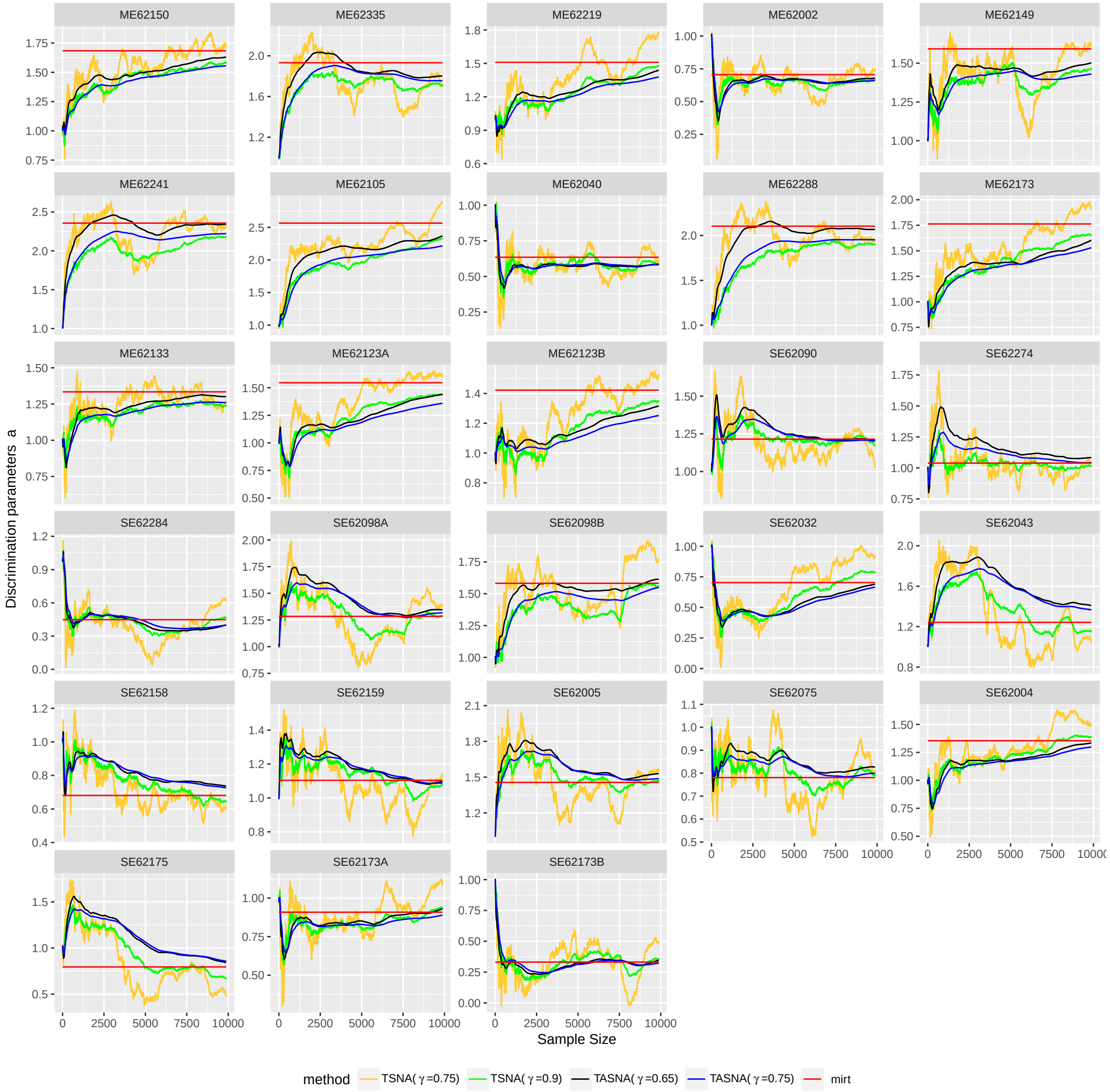

Figure D1: Real-time trajectory plots of item discrimination parameters $\boldsymbol{a}$.

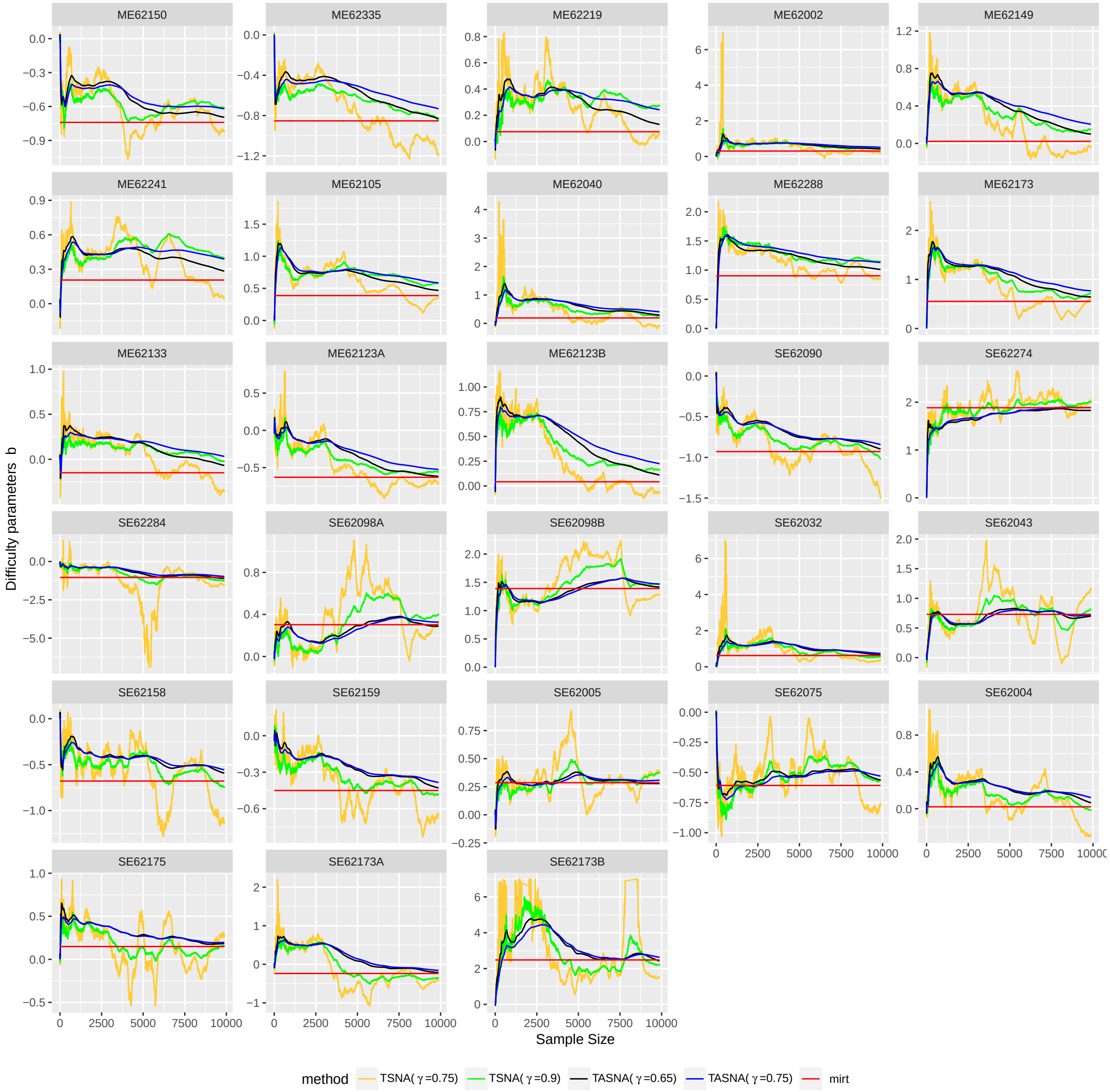

Figure D2: Real-time trajectory plots of item difficulty parameters $\boldsymbol{b}$.

References

Bercu, B., Godichon, A., & Portier, B. (2020). An efficient stochastic newton algorithm for parameter estimation in logistic regressions. *SIAM Journal on Control and Optimization, 58*(1), 348–367.

Boyer, C., & Godichon-Baggioni, A. (2023). On the asymptotic rate of convergence of stochastic newton algorithms and their weighted averaged versions. *Computational Optimization and Applications, 84*(3), 921–972.

Cui, C., Wang, C., & Xu, G. (2024). Variational Estimation for Multidimensional Generalized Partial Credit Model. *Psychometrika*, 1–29.

Duflo, M. (2013). *Random iterative models* (Vol. 34). Springer Science & Business Media.

Goda, T., Kazashi, Y., & Tanaka, K. I. (2024). How Sharp Are Error Bounds?–Lower Bounds on Quadrature Worst-Case Errors for Analytic Functions. *SIAM Journal on Numerical Analysis, 62*(5), 2370–2392.
